# Diastereoselective synthesis of highly substituted cyclohexanones and tetrahydrochromene-4-ones via conjugate addition of curcumins to arylidenemalonates

**DOI:** 10.3762/bjoc.20.177

**Published:** 2024-08-15

**Authors:** Deepa Nair, Abhishek Tiwari, Banamali Laha, Irishi N N Namboothiri

**Affiliations:** 1 Department of Chemistry, Indian Institute of Technology Bombay, Mumbai, 400 076, India

**Keywords:** arylidenemalonates, curcumins, cyclohexanones, diastereoselective synthesis, Michael reaction, tetrahydrochromenones

## Abstract

A cascade inter–intramolecular double Michael strategy for the synthesis of highly functionalized cyclohexanones from curcumins and arylidenemalonates is reported. This strategy works in the presence of aqueous KOH using TBAB as a suitable phase transfer catalyst at room temperature. The functionalized cyclohexanones are formed as major products in moderate to excellent yields with complete diastereoselectivity in most cases. A triple Michael adduct, tetrahydrochromen-4-one, is also formed as a side product in a few cases with excellent diastereoselectivity.

## Introduction

**Figure 1:**
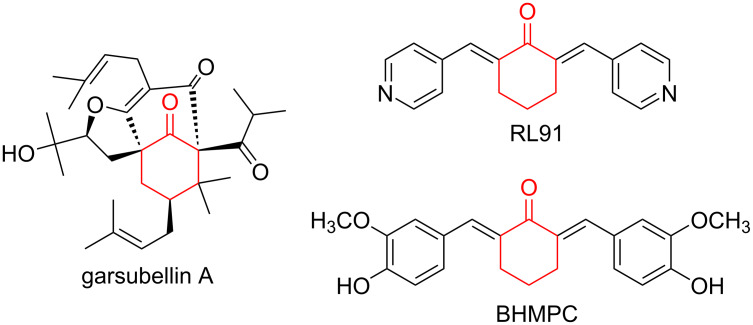
Biologically active derivatives of cyclohexanones.

There is considerable interest in the stereoselective synthesis of the cyclohexanone skeleton as it constitutes the core structure in many natural products and pharmaceutical drugs [1–2]. Garsubellin A with a cyclohexanone skeleton is a potent inducer of choline acetyltransferase (ChAT) and could be used for the treatment of Alzheimer's disease [3–4]. Likewise, RL91 and BHMPC are active for selective cell growth inhibition of the resistant lines (Figure 1) [5–6]. Their synthesis mainly involves a cascade Michael–aldol reaction between enones and suitable Michael donors such as β-ketosulfones, β-diketones, or double Michael addition of γ,δ-unsaturated-β-keto esters or Nazarov reagents with suitable acceptors such as nitroalkenes or alkylideneazalactones [7–10].

**Scheme 1:**
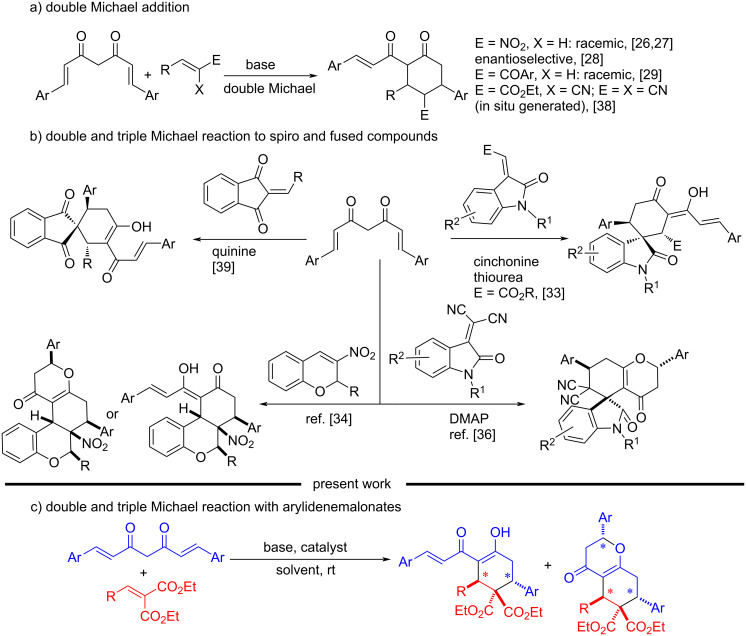
The Michael donor–acceptor reactivity of curcumin: previous vs present work.

From the perspective of an active methylene containing organic moiety, curcumin and its analogs serve as inexpensive and readily available starting materials for cascade/domino reactions. Curcumin is the key component present in turmeric and is responsible for its various biological activities. Turmeric has been used in many forms for potential health benefits and as a traditional spice for centuries [11]. Curcumin exhibits diverse biological properties such as anticancer, anti-HIV protease, anti-ageing, anti-inflammatory and anti-oxidant, to name a few [12–23]. Considering the fact that curcumin’s uniqueness is associated with its ability to function as a multifunctional substrate in various organic reactions, especially as a Michael donor at the central methylene carbon, and Michael acceptor at the enone vinyl carbon. Therefore, it would be interesting to develop novel methodologies using curcumin and its non-natural analogs as key starting materials [24]. Because of its multifaceted reactive site, curcumin showcases its Michael donor–acceptor ability in different ways, such as simple Michael addition, [4 + 2] annulation, Michael addition followed by cyclization or one-pot multicomponent reactions (MCR), etc. (Scheme 1) [25]. In 2011, our group reported the reactivity of curcumin as a Michael donor–acceptor with nitroalkenes, resulting in multi-substituted cyclohexanones through a cascade inter–intramolecular double Michael addition process with high diastereoselectivity [26–27]. Subsequently, the enantioselective versions of the above reaction and a similar diastereoselective cascade Michael addition–cyclization of curcumins with chalcones to synthesize functionalized cyclohexanones have been reported [28–29]. On the other hand, diastereoselective cascade Michael addition–cyclization of curcumins with α-bromonitroalkenes and α-halodicyclopentadienones afforded functionalized dihyrofurans [26,30]. The reaction of curcumins with azodicarboxylates, Morita–Baylis–Hillman derived nitroallylic acetates and α-hydrazinonitroalkenes were also investigated [29,31–32]. Very recently, our group has exploited 3-olefinic oxindoles and nitrochromenes to unfold the reactivities of curcumins [33–34].

Other groups have also investigated the Michael donor–acceptor reactivity of curcumins [25]. For instance, a quinine-thiourea catalyzed Michael addition of curcumins to nitroalkenes reported by Ye et al. stopped at the single Michael addition stage [35]. In the subsequent year, Yan et al. demonstrated a cascade triple-Michael (Michael/Michael/oxa-Michael) reaction between curcumins and isatylidene malononitriles, giving spiro-oxindoles in excellent yields and diastereoselectivities [36]. Sahu et al. introduced a one-pot multicomponent reaction of curcumin, arylaldehyde and 2-aminobenzothiazole to synthesize functionalized pyrimidobenzothiazoles [37]. An in situ generated conjugated α-cyanoester/malononitrile has been successfully employed as substrate in the double Michael reaction with curcumins by Lalitha et al. [38]. An organocatalytic cascade double Michael reaction between curcumins and 2-arylidene-1,3-indandiones was reported by Zhang and co-workers using quinine as a catalyst, giving multicyclic spiro-1,3-indandiones in moderate yields with enantioselectivities as well as diastereoselectivities [39]. However, to the best of our knowledge, arylidenemalonates have not been employed as substrates in the cascade reaction of curcumins employing a biphasic solvent system for the synthesis of highly functionalized cyclohexanones and tetrahydrochromenes.

In view of the above, we report herein cascade double and triple Michael reactions of curcumins with arylidenemalonates in the presence of a base (KOH) and a phase-transfer catalyst (PTC) in a biphasic medium (toluene–H_2_O) at room temperature, leading to highly functionalized cyclohexanones and tetrahydrochromenones as major and minor products, respectively, in moderate to high yield and excellent diastereoselectivity.

## Results and Discussion

Initially, the reaction of **1a** with **2a** was carried out with 3.0 equiv of aq KOH as base and toluene as a solvent in the presence of 20 mol % tetrabutylammonium bromide (TBAB) as a phase-transfer catalyst at room temperature for 24 h (Table 1, entry 1). To our delight, the formation of double Michael addition product **3a** was observed in 31% yield. The reaction with 6.0 equiv of KOH resulted in an increase in the yield of **3a** (72%) and also the formation of **4a** in low yield (13%, Table 1, entry 2). Next, the THF–toluene solvent system was employed in the absence and presence of TBAB, affording **3a** in 56%, 49% and 60% yields, respectively (Table 1, entries 3–5). As expected, no product formation was noted when the reaction was investigated without TBAB (Table 1, entry 6) and the same observation was made in the absence of base (Table 1, entry 7). Other bases such as Cs_2_CO_3_, NaOH and tetramethylguanidine (TMG) were screened in toluene as the solvent of choice (Table 1, entries 8–10), but only TMG provided the desired product **3a** though in lower yield (63%, Table 1, entry 10). Interestingly, TMG furnished 55% yield of **3a** in acetonitrile as solvent (Table 1, entry 11). Changing the PTC to TBAI did not have a significant effect on the yield or formation of respective products (Table 1, entry 12). Based on the optimization studies, a combination of KOH in toluene and TBAB (Table 1, entry 2) as phase-transfer catalyst was chosen as the best conditions. Although KOH could saponify the ester group, our methodology involves a biphasic reaction medium where the starting material, viz. arylidenemalonate, remains in the organic layer and prevents itself from undergoing saponification because it requires either aqueous or alcoholic (MeOH or EtOH) medium. Furthermore, deprotonation of curcumin predominates over saponification under our mild conditions (room temperature). Overall, the reaction proceeds smoothly without any unwanted side reactions such as saponification of the ester groups.

**Table 1:** Reaction optimisation using phase-transfer catalysts.^a^

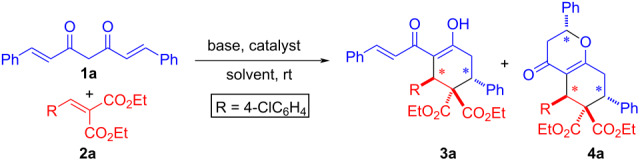						

Entry	Base [equiv]	Catalyst [mol %]	Solvent	Time [h]	**3a** Yield [%]^b^	**4a** Yield [%]^b^

1	KOH (3.0)	TBAB (20)	toluene	24	31	nd
**2**	**KOH (6.0)**	**TBAB (20)**	**toluene**	**16**	**72**	**13**
3	KOH (6.0)	TBAB (20)	THF–toluene (2:8)	48	56	traces
4	KOH (6.0)	–	THF–toluene (2:8)	48	49	traces
5	KOH (6.0)	TBAB (20)	THF–toluene (8:2)	48	60	traces
6^c^	KOH (6.0)	–	toluene	72	nd	nd
7^c^	–	TBAB (20)	toluene	72	nd	nd
8^d^	Cs_2_CO_3_ (6.0)	TBAB (20)	toluene	24	nd	nd
9^d^	NaOH (6.0)	TBAB (20)	toluene	48	nd	nd
10	TMG (6.0)	TBAB (20)	toluene	72	63	nd
11	TMG (6.0)	TBAB (20)	acetonitrile	16	55	nd
12	KOH (6.0)	TBAI (20)	toluene	72	69`	11

^a^Reaction scale: **1a** (0.1 mmol, 1 equiv), **2a** (0.12 mmol, 1.2 equiv), solvent (1.5 mL); ^b^after silica-gel column chromatography; ^c^no reaction; ^d^complex mixture; nd = not detected.

Having optimized the reaction conditions, we proceeded to examine the substrate scope of the developed methodology (Table 2). At first, various arylidenemalonates **2** were tested with a phenyl analog of curcumin **1**. The reaction of **1a** with arylidene malonates **2a–c**, bearing weakly electron-withdrawing *para*-substituents, produced the corresponding double Michael adducts **3a–c**, respectively, in good to high yields (55–75%) with excellent diastereoselectivity (Table 2, entries 1–3). At the same time, the corresponding triple Michael adducts **4a–c** were also formed as minor products (12–36%). There was no reaction with *ortho*-nitro-substituted arylidenemalonate **2d** under the optimized conditions which is attributable to the increase in electron density at the carbon β to the ester group thus inhibiting the Michael addition of curcumin (Table 2, entry 4). Arylidenemalonate **2e**, bearing a weakly electron-donating substituent, reacted with **1a** to afford the double Michael adduct **3e** in moderate yield (46%)**,** and the triple Michael adduct **4e** was not detected (Table 2, entry 5). Bulky 1-naphthyl analog **2f** delivered the double Michael adduct **3f** as the only product in a very good yield (73%, Table 2, entry 6). Heteroarylidenemalonate **2g** also reacted well with **1a**, affording **3g** in 54% yield (Table 2, entry 7). Unfortunately, no reaction occurred with aliphatic valeryl derivate **2h**, presumably due to the competitive deprotonation of the active methylene group of curcumin and the allylic γ-position of the diester and/or the +I effect of the alkyl group which deactivates the β-position of the diester (Table 2, entry 8).

**Table 2:** Scope of alkylidenemalonates and curcumins.^a^

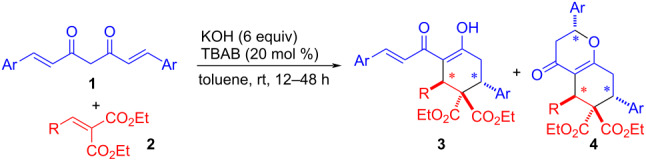									

Entry	**1**, Ar	**2**, R	Time [h]	**3**	Yield [%]^b^	dr	**4**	Yield [%]^b^	dr

1	**1a**, Ph	**2a**, 4-ClC_6_H_4_	16	**3a**	72	>95:5	**4a**	13	>95:5
2	**1a**, Ph	**2b**, 4-FC_6_H_4_	18	**3b**	55	>95:5	**4b**	36	>95:5
3	**1a**, Ph	**2c**, 3-BrC_6_H_4_	24	**3c**	75	>99:5	**4c**	12	>95:5
4^c^	**1a**, Ph	**2d**, 2-NO_2_C_6_H_4_	36	**3d**	nd	–	**4d**	nd	–
5	**1a**, Ph	**2e**, 4-MeC_6_H_4_	22	**3e**	46	>95:5	**4e**	nd	–
6	**1a**, Ph	**2f**, 1-naphthyl	12	**3f**	73	>95:5	**4f**	traces	–
7	**1a**, Ph	**2g**, 2-furyl	16	**3g**	54	>95:5	**4g**	traces	–
8^c^	**1a**, Ph	**2h**, valeryl	24	**3h**	nd	–	**4h**	nd	–
9	**1b**, 4-MeC_6_H_4_	**2a**, 4-ClC_6_H_4_	36	**3i**	43	>95:5	**4i**	traces	–
10	**1c**, 4-BrC_6_H_4_	**2a**, 4-ClC_6_H_4_	48	**3j**	35	>95:5	**4j**	nd	–
11	**1d**, 1-naphthyl	**2a**, 4-ClC_6_H_4_	36	**3k**	44	>95:5	**4k**	traces	–
12	**1e**, 4-MeOC_6_H_4_	**2b**, 4-FC_6_H_4_	18	**3l**	54	79:21	**4l**	nd	–
13	**1e**, 4-MeOC_6_H_4_	**2e**, 4-MeC_6_H_4_	48	**3m**	42	>95:5	**4m**	16	>95:5
14	**1f**, 2-Furyl	**2i**, Ph	18	**3n**	56	>95:5	**4n**	nd	–

^a^Reaction scale: **1** (0.1 mmol, 1 equiv), **2** (0.12 mmol, 1.2 equiv), TBAB (0.02 mmol, 20 mol %), toluene (1.5 mL); ^b^after silica-gel column chromatography; ^c^No reaction.; nd = not detected.

In the next set of experiments, different curcumins were examined while keeping **2a** as the model arylidenemalonate (Table 2, entries 9–11). The reaction of *p*-tolyl and *p*-bromophenyl analogs of curcumin **1b**,**c** led to the formation of double Michael adducts **3i**–**j** in moderate yields (35–43%) over prolonged reaction time (36–48 h, Table 2, entries 9 and 10). The 1-naphthyl analog of curcumin **1d** gave a moderate yield (44%) of the double Michael adduct **3k** (Table 2, entry 11). The *p*-anisylcurcumin **1e** reacted with **2b** and furnished the double Michael adduct **3l** in good yield (54%) but moderate diastereomeric ratio (79:21, Table 2, entry 12). Next, *p*-anisylcurcumin **1e** when treated with *p*-tolylidenemalonate **2e** furnished the double Michael adduct **3m** in decent yield (42%) with excellent diastereoselectivity (Table 2, entry 13). Similarly, the reaction of benzylidenemalonate **2i** with furylcurcumin **1f** resulted in double Michael adduct **3n** in 56% yield (Table 2, entry 14).

**Scheme 2:**
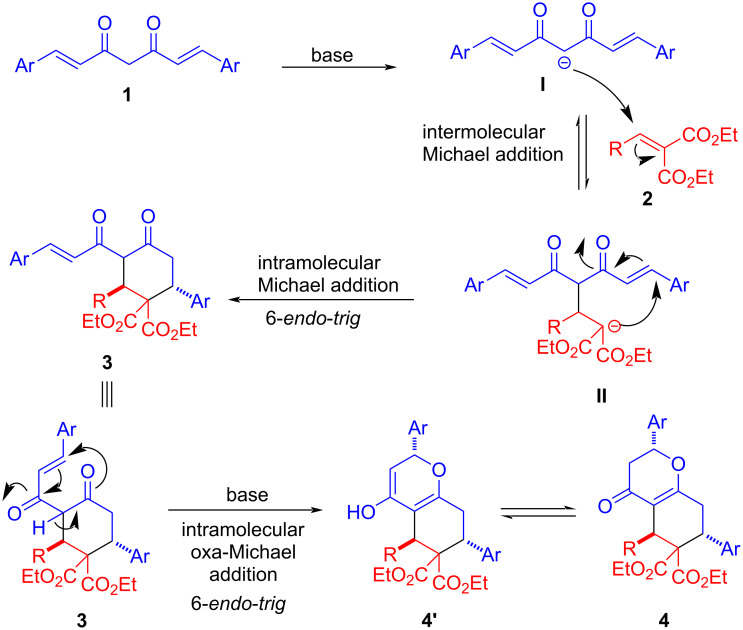
A plausible reaction mechanism.

A plausible mechanism for the cascade double and triple Michael reactions is shown in Scheme 2. At first, the enolate **I** of curcumin **1** adds to arylidenemalonate **2** in a Michael fashion resulting enolate **II**. The ester enolate **II** might remain in equilibrium with 1,3-dicarbonyl enolate **I**, but the former would be trapped via cyclization involving a diastereoselective *6-endo-trig* intramolecular Michael addition to the enone moiety leading to highly substituted cyclohexanone **3**. The formation of triple Michael adduct **4** can be attributed to the enolate **3** undergoing yet another diastereoselective *6-endo-trig* intramolecular oxa-Michael addition.

**Figure 2:**
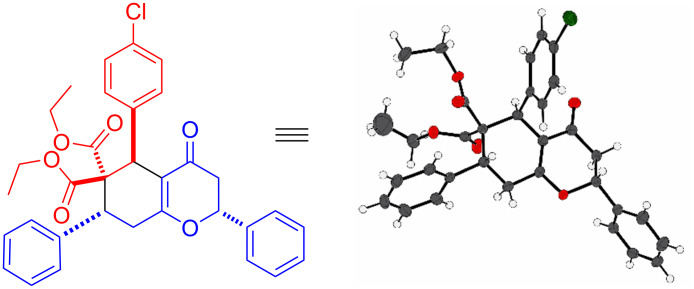
X-ray structure of **4a** (CCDC 2351387).

The structure and stereochemistry of compounds **3** and **4** were confirmed by ^1^H-^1^H-COSY and ^1^H-^1^H-NOESY NMR experiments. This was further unambiguously established by single crystal X-ray analysis of a representative compound **4a** (Figure 2). These studies confirmed that R and Ar in the cyclohexane ring of compounds **3** and **4** are *trans* to each other and the Ar in the dihydropyran ring of **4** is *trans* to R and *cis* to Ar in the cyclohexane ring.

**Figure 3:**
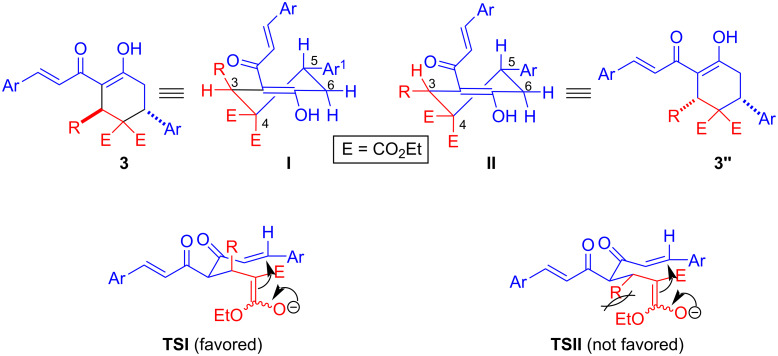
Origin of stereoselectivity in the double Michael addition.

The diastereoselectivity observed in the above cascade double Michael reaction of curcumins **1** with arylidenemalonate **2** can be explained in terms of the relative stereochemistry of the substituents in the enolate arising from the first Michael addition (Figure 3). Comparison of the two possible transition states **TSI** and **TSII** for second Michael addition suggests that a severe 1,3-allylic strain destabilizes **TSII** where R is equatorial. Such a strain is avoided by R adopting axial orientation as in **TSI**, which is favoured, leading to the observed product **3**. This has also been seen in previous cases where nitroalkenes and chalcones have been employed as acceptors in the reaction of curcumins [26,29].

**Scheme 3:**
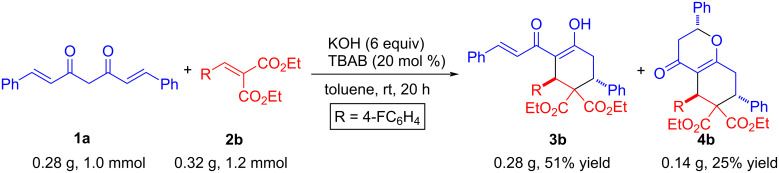
Scale-up reaction.

In order to demonstrate the synthetic utility of our methodology, we performed a scale-up reaction with representative starting materials, viz*.* **1a** and **2b** on a 1.0 mmol scale (Scheme 3). The reaction required slightly more time and resulted in the corresponding double and triple Michael adducts **3b** and **4b**, respectively, in marginally lower yields (see also Table 2, entry 2).

## Conclusion

A hitherto unexplored cascade double and triple Michael reactions of curcumins with arylidenemalonates is reported here. The double Michael reactions lead to highly functionalized cyclohexanones as exclusive or predominant products with excellent diastereoselectivity. On the other hand, triple Michael reactions took place in a few cases, affording highly functionalized tetrahydrochromen-4-ones as minor products with complete diastereoselectivity. The reactions were carried out in the presence of aq KOH using TBAB as a suitable phase transfer catalyst in a biphasic medium at room temperature. The scalability of the reaction has also been demonstrated. Our future efforts will involve performing an asymmetric version of this reaction using chiral phase-transfer catalysts and the results will be reported in due course.

## Supporting Information

File 1 Experimental procedures and characterization data.

File 2 Copies of NMR spectra of all new compounds.

File 3 Crystallographic information file of compound **4a**.

## Data Availability

All data that supports the findings of this study is available in the published article and/or the supporting information to this article.
