# Correction: Diastereoselective synthesis of highly substituted cyclohexanones and tetrahydrochromene-4-ones via conjugate addition of curcumins to arylidenemalonates

**DOI:** 10.3762/bjoc.21.93

**Published:** 2025-06-16

**Authors:** Deepa Nair, Abhishek Tiwari, Banamali Laha, Lakshminarayana Satham, Irishi N N Namboothiri

**Affiliations:** 1 Department of Chemistry, Indian Institute of Technology Bombay, Mumbai, 400 076, India

**Keywords:** arylidenemalonates, curcumins, cyclohexanones, diastereoselective synthesis, Michael reaction, tetrahydrochromenones

Lakshminarayana Satham is acknowledged as additional co-author of our original publication. It was subsequently determined that Lakshminarayana Satham made significant contributions to the published research.

The Funding section is updated as follows:

DN thanks UGC India for a Senior Research Fellowship and ART thanks IIT Bombay for an Institute Postdoctoral Fellowship (IPDF). BL thanks the Ministry of Education, Government of India, for the Prime Minister’s Research Fellowship (PMRF) and LS thanks CSIR India for a Senior Research Fellowship. INN thanks SERB India for financial support.

