# Supplementary material for: Diastereoselective synthesis of highly substituted cyclohexanones and tetrahydrochromene-4-ones via conjugate addition of curcumins to arylidenemalonates
Source: Beilstein J Org Chem. 2024 Aug 15;20:2016–23. doi: 10.3762/bjoc.20.177 (PMC11331540; doi:10.3762/bjoc.20.177)
Supplement: File 1 — Experimental procedures and characterization data. [file Beilstein_J_Org_Chem-20-2016-s001.pdf]

BEILSTEIN JOURNAL OF ORGANIC CHEMISTRY

Supporting Information

for

Diastereoselective synthesis of highly substituted cyclohexanones and tetrahydrochromene-4-ones via conjugate addition of curcumins to arylidenemalonates

Deepa Nair, Abhishek Tiwari, Banamali Laha and Irishi N. N. Namboothiri

Beilstein J. Org. Chem. **2024,** *20,* 2016–2023. doi:10.3762/bjoc.20.177

Experimental procedures and characterization data

License and Terms: This is a supporting information file under the terms of the Creative Commons Attribution License (https://creativecommons.org/licenses/by/4.0). Please note that the reuse, redistribution and reproduction in particular requires that the author(s) and source are credited and that individual graphics may be subject to special legal provisions.
The license is subject to the *Beilstein Journal of Organic Chemistry* terms and conditions: (https://www.beilstein-journals.org/bjoc/terms)

Entry	Table of contents	Page
I	Table S1. Selected X-ray data and structure refinement for **4a**	S1
II	Experimental section	S2
1	General information	S2
2	General procedure for cascade Michael addition-cyclization of curcumins **1** with arylidenemalonates **2**	S2
3	Characterization data	S3–S8
4	References	S8

Table S1. Selected X-ray data and structure refinement for 4a (CCDC 2351387)

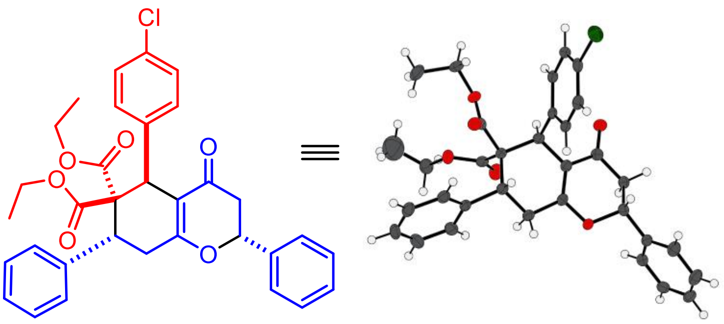

Table 1. Crystal data and structure refinement for 4a.

Identification code	INN-DN-349-B	
Empirical formula	C33 H31 Cl O6	
Formula weight	559.03	
Temperature	100(2) K	
Wavelength	0.71075 Å	
Crystal system	Triclinic	
Space group	P -1	
Unit cell dimensions	a = 13.149(3) Å	α= 69.600(11)°.
	b = 13.243(3) Å	β= 74.779(12)°.
	c = 18.317(4) Å	γ = 78.876(12)°.
Volume	2866.5(11) Å^3	
Z	4	

Density (calculated)	1.295 Mg/m^3
Absorption coefficient	0.177 mm^{-1}
F(000)	1176
Crystal size	0.20 x 0.14 x 0.08 mm^3
Theta range for data collection	3.14 to 25.00°.
Index ranges	-15<=h<=15, -15<=k<=15, -21<=l<=21
Reflections collected	43799
Independent reflections	9938 [R(int) = 0.0602]
Completeness to theta = 25.00°	98.4 %
Absorption correction	Numerical
Max. and min. transmission	0.9859 and 0.9654
Refinement method	Full-matrix least-squares on F^2
Data / restraints / parameters	9938 / 0 / 721
Goodness-of-fit on F^2	1.232
Final R indices [I>2sigma(I)]	R1 = 0.0648, wR2 = 0.1788
R indices (all data)	R1 = 0.0903, wR2 = 0.2277
Largest diff. peak and hole	0.845 and -1.117 e.$Å^{-3}$

Experimental section

General information

The reagents and solvents were purchased from commercial sources and were used as received unless mentioned otherwise. The reactions were monitored by thin-layer chromatography (TLC). The melting points recorded are uncorrected. NMR spectra (^1H, ^1H decoupled ^{13}C, ^1H-^1H COSY, ^1H-^1H NOESY) were recorded with TMS as the internal standard. The coupling constants (*J* values) are given in Hz. High-resolution mass spectra (HRMS) were recorded under ESI Q-TOF conditions. X-ray data were collected on a diffractometer equipped with graphite monochromated Mo Kα radiation. The structure was solved by direct methods shelxs97 and refined by full-matrix least squares against F^2 using shelxl97 software. Curcumins[1] and arylidenemalonates[2] were prepared using the literature method.

General procedure for cascade Michael addition–cyclization of curcumins 1 with arylidenemalonates 2

To a mixture of arylidenemalonate **2** (0.12 mmol, 1.2 equiv), TBAB (7.2 mg, 20 mol %) in toluene (1.5 mL) was added curcumin **1** (0.1 mmol, 1.0 equiv) and aqueous KOH solution (33.6 mg in 0.125 mL water) at room temperature. The resulting reaction mixture was stirred until the reaction was completed (monitored by TLC, see also Table 2 and Scheme 3, main text). After completion of the reaction, the reaction mixture was concentrated in vacuo. The crude product was purified by silica gel column chromatography by eluting with ethyl acetate–pet ether (for ratio, see experimental data for individual compounds).

Diethyl 2-(4-chlorophenyl)-3-cinnamoyl-4-oxo-6-phenylcyclohexane-1,1-dicarboxylate (3a)

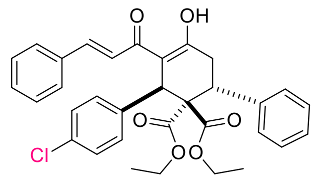

3a

Yellow oil; 72% (40 mg), dr > 95:5; R_f: 0.7 (15% ethyl acetate in pet ether); IR (KBr, cm^{-1}) 3397 (br s), 2937 (w), 1725 (s), 1626 (m), 1594 (m), 1512 (s), 1264 (vs), 1244 (s), 1141 (m), 1026 (m); ^{1}H NMR (400 MHz, $CDCl_3$) δ 0.92 (dd collapsed to t, *J* = 7.2 Hz, 3H), 1.17 (dd collapsed to t, *J* = 7.2 Hz, 3H), 2.80, 3.40 (ABq, *J* = 19.5 Hz, the upper half further split into d, *J* = 7.5 Hz and the lower half further split into d, *J* = 10.7 Hz, 2H), 3.72, 3.77 (ABqq, *J* = 10.7, 7.2 Hz, 2H), 3.83 (dd, *J* = 10.7, 7.5 Hz, 1H), 4.13, 4.29 (ABqq, *J* = 10.7, 7.2 Hz, 2H), 4.92 (s, 1H), 6.72 (d, *J* = 15.5 Hz, 1H), 7.20-7.29 (m, 7H), 7.34 (d, *J* = 8.8 Hz, 2H), 7.35-7.37 (unresolved m, 5H), 7.64 (d, *J* = 15.5 Hz, 1H), 17.04 (s, 1H); ^{13}C NMR (100 MHz, $CDCl_3$) δ 13.6, 14.0, 37.5, 39.5, 46.4, 61.4, 61.8, 63.0, 109.0, 120.2, 127.4, 127.7, 128.4, 128.8, 129.1, 130.5, 130.6, 131.2, 133.9, 135.1, 139.1, 139.9, 143.3, 168.8, 169.5, 185.2, 186.8; HRMS (ES+) calcd for $C_{33}H_{32}{}^{35}ClO_6$ (MNa^+) 559.1887, found 581.1882.

Diethyl 5-(4-chlorophenyl)-4-oxo-2,7-diphenyl-3,4,7,8-tetrahydro-2*H*-chromene-6,6(5*H*)-dicarboxylate (4a)

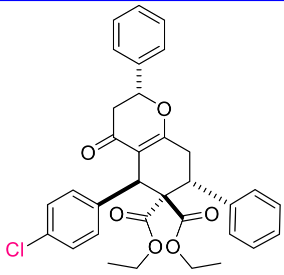

4a

White solid; yield 13% (8 mg), dr > 95:5; R_f: 0.3 (15% ethyl acetate in pet ether); m.p. 148 °C; IR (KBr, cm^{-1}) 3012 (m), 2983 (s), 2936 (m), 1725 (vs), 1668 (m), 1628 (s), 1490 (m), 1414 (s), 1403 (s), 1242 (vs), 1218 (s), 1201 (s), 1156 (s), 1092 (m), 1014 (m), 759 (vs); ^{1}H NMR (400 MHz, $CDCl_3$) δ 0.95 (dd collapsed to t, *J* = 7.1 Hz, 3H), 1.11 (dd collapsed to t, *J* = 7.1 Hz, 3H), 2.65 (dd, *J* = 17.1, 3.2 Hz, 1H), 2.91 (dd, *J* = 17.1, 14.4 Hz, 1H), 2.93, 3.04 (ABq, *J* = 19.1 Hz, the upper half further split into d, *J* = 9.8 Hz and the lower half further split into d, *J* = 6.4 Hz, 2H), 3.60 (dq, *J* = 10.8, 7.1 Hz, 1H), 3.78 (dq, *J* = 10.8, 7.1 Hz, 1H), 3.92 (dd, *J* = 9.8, 6.4 Hz, 1H), 3.99 (dq, *J* = 10.8, 7.1 Hz, 1H), 4.20 (dq, *J* = 10.8, 7.1 Hz, 1H), 4.87 (s, 1H), 5.42 (dd, *J* = 14.4, 3.2 Hz, 1H), 7.22-7.26 (m, 5H), 7.28-7.30 (unresolved m, 4H), 7.36-7.42 (m, 5H); ^{13}C NMR (100 MHz, $CDCl_3$) δ 13.7, 13.9, 33.8, 40.8, 43.1, 43.2, 61.2, 61.4, 61.9, 80.1, 113.8, 126.3, 127.5, 127.8, 128.3, 129.0, 129.0, 130.2, 131.0, 133.3, 138.2, 139.3, 139.5, 169.0, 169.6, 169.9, 189.7; HRMS (ES+) calcd for $C_{33}H_{32}{}^{35}ClO_6$ (MH^+) 559.1882, found 559.1887.

Diethyl 3-cinnamoyl-2-(4-fluorophenyl)-4-oxo-6-phenylcyclohexane-1,1-dicarboxylate (3b)

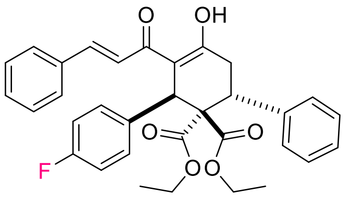

3b

Yellow solid; yield 55% (30 mg), dr > 95:5; R_f: 0.4 (12% ethyl acetate in pet ether); mp 164 °C; IR (KBr, cm^{-1}) 3435 (br w), 2983 (m), 2925 (m), 1728 (s), 1628 (m), 1603 (m), 1239 (s), 1205 (m), 702 (m); ^{1}H NMR (500 MHz, $CDCl_3$) δ 0.96 (dd collapsed to t, *J* = 7.2 Hz, 3H), 1.21 (dd collapsed to t, *J* = 7.0 Hz, 3H), 3.04 (Unresolved 2×d, = 6.4 Hz, 2.9 Hz, 2H), 3.74 (ABq collapsed to t, *J* = 7.2 Hz) 3.88 (Unresolved dd, *J* = 10.6, 7.4 Hz, 1H), 4.16, 4.33 (ABdq, *J* = 14.21, 10.8, 7.1, 10.8 Hz, 2H), 4.97 (s, 1H), 6.79 (d, *J* = 15.5 Hz, 1H), 7.09 (t, *J* = 8.7 Hz, 2H), 7.27-7.23 (m, 3H), 7.33-7.30 (m, 4H), 7.38 (Unresolved m, 5H), 7.64 (d, *J* = 15.5 Hz, 1H); Confirmed by ^{1}H-^{1}H COSY and NOESY experiments; ^{13}C NMR (125 MHz, $CDCl_3$) δ 13.7, 14.0, 37.5, 39.4, 46.3, 61.3, 61.8, 63.1, 109.2, 115.6 (d, $J_{C\text{-}F}$ = 21.3 Hz), 120.4, 127.4, 127.6, 128.4, 129.1, 130.6, 131.5 (d, $J_{C\text{-}F}$ = 7.5 Hz),

135.1, 137.2, 139.2, 143.1, 162.4 (d, $J_{C\text{-}F}$ = 247.5 Hz), 168.9, 169.6, 185.3, 186.6; ^{19}F NMR (470 MHz, $CDCl_3$) δ -114.24; HRMS (ES+) calcd for $C_{33}H_{31}FO_6Na$ (MNa^+) 565.1997, found 565.2012.

Diethyl 5-(4-fluorophenyl)-4-oxo-2,7-diphenyl-3,4,7,8-tetrahydro-2*H*-chromene-6,6(5*H*)-dicarboxylate (4b)

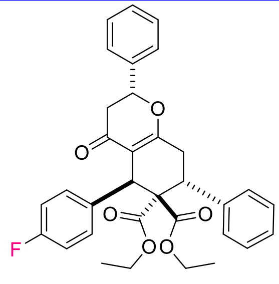

4b

Yellow solid; yield 36% (20 mg), dr > 95:5; R_f: 0.2 (15% ethyl acetate in pet ether); mp 158 °C; IR (KBr, cm^{-1}) 3062 (w), 2983 (m), 2960 (m), 1726 (vs), 1668 (m), 1630 (s), 1604 (m), 1507 (s), 1406 (s), 1243 (vs), 1201 (s), 1159 (s), 759 (s), 700 (s); ^{1}H NMR (500 MHz, $CDCl_3$) δ 0.96 (dd collapsed to t, *J* = 7.2 Hz, 3H), 1.12 (dd collapsed to t, *J* = 7.1 Hz, 3H), 2.63 (dd, *J* = 17.2, 3.2 Hz, 1H), 2.90 (overlapped dd, *J* = 17.1, 3.1 Hz, 1H), 2.95, 3.02 (overlapped ABq, *J* = 19.1 Hz, 6.43 Hz, 2H), 3.60 (dd, *J* = 10.6, Hz, 7.1 Hz, 1H), 3.60 (dd, *J* = 10.6, 7.1 Hz, 1H), 3.93 (dd, *J* = 10 Hz, 6.5 Hz, 1H), 4.01 (dd, *J* = 10.8 Hz, 7.2 Hz, 1H), 4.21 (dd, *J* = 10.2 Hz, 7.1 Hz, 1H), 4.88 (s, 1H), 5.42 (dd, *J* = 14.3 Hz, 3.2 Hz, 1H), 7.00 (t, *J* = 8.6 Hz, 2H), 7.21-7.26 (m, 6H), 7.30-7.33 (dd, *J* = 8.46 Hz, 5.5 Hz, 2H), 7.37-42 (m, 5H); ^{13}C NMR (100 MHz, $CDCl_3$) δ 13.5, 13.7, 33.6, 40.5, 42.9, 43.0, 60.9, 61.2, 61.8, 79.9, 113.8, 114.9 (d, $J_{C\text{-}F}$ = 21.0 Hz), 126.1, 127.2, 127.6, 128.8 (×2), 130.0, 131.0 (d, $J_{C\text{-}F}$ = 8.4 Hz), 136.2 (×2), 138.0, 139.3, 162.0 (d, $J_{C\text{-}F}$ = 246.7 Hz), 168.8, 169.5, 189.5; ^{19}F NMR (470 MHz, $CDCl_3$) δ -115.40; HRMS (ES+) calcd for $C_{33}H_{32}FO_6$ (MH^+) 543.2177, found 543.2174.

Diethyl 2-(3-bromophenyl)-3-cinnamoyl-4-oxo-6-phenylcyclohexane-1,1-dicarboxylate (3c)

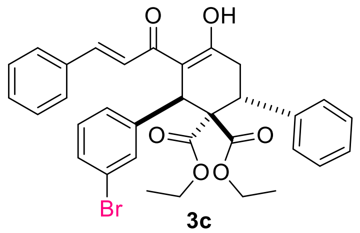

3c

Yellow solid; yield 75% (45 mg), dr > 95:5; R_f: 0.5 (10% ethyl acetate in pet ether); mp 175 °C; IR (KBr, cm^{-1}) 3458 (br w), 2983 (m), 2929 (m), 1726 (s), 1629 (m), 1604 (m), 1507 (m), 1297 (m), 1275 (m), 1242 (s), 1206 (m), 912 (s), 760 vs), 736 (vs); ^{1}H NMR (400 MHz, $CDCl_3$) δ 0.93 (dd collapsed to t, *J* = 7.2 Hz, 3H), 1.18 (dd collapsed to t, *J* = 7.2 Hz, 3H), 3.04 (ABqd collapsed to d, *J* = 9.4 Hz, 2H), 3.79, 3.84 (ABqq, *J* = 10.7, 7.2 Hz, 2H), 3.89 (dd collapsed to t, *J* = 9.4 Hz, 1H), 4.15 (dq, *J* = 10.7, 7.2 Hz, 1H), 4.33 (dq, *J* = 10.7, 7.2 Hz, 1H), 4.94 (s, 1H), 6.76 (d, *J* = 15.5 Hz, 1H), 7.24-7.29 (m, 5H), 7.30-7.33 (m, 2H), 7.38-7.41 (unresolved m, 5H), 7.43-7.46 (m, 1H), 7.54 (s, 1H), 7.64 (d, *J* = 15.5 Hz, 1H), 17.03 (s, 1H); ^{13}C NMR (125 MHz, $CDCl_3$) δ 13.6, 14.0, 37.3, 39.4, 46.7, 61.4, 61.8, 63.0, 108.7, 120.4, 122.7, 127.4, 127.6, 128.4, 128.5, 129.1, 130.2, 130.6 (× 2), 131.0, 133.0, 135.0, 139.0, 143.2, 143.9, 168.7, 169.4, 185.4, 186.6; HRMS (ES+) calcd for $C_{33}H_{31}{}^{79}BrO_6Na$ (MNa^+) 625.1196, found 625.1190.

Diethyl 5-(3-bromophenyl)-4-oxo-2,7-diphenyl-3,4,7,8-tetrahydro-2*H*-chromene-6,6(5*H*)-dicarboxylate (4c)

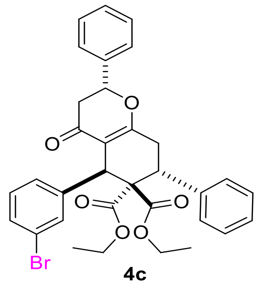

4c

Yellow oil; yield 12% (8 mg), >95:5; R_f: 0.2 (12% ethyl acetate in pet ether); IR (KBr, cm^{-1}) 2981 (w), 2962 (m), 2935 (m), 1725 (s), 1670 (w), 1629 (m), 1611 (m), 1513 (vs), 1405 (m), 1252 (vs), 1159 (s), 1034 (m), 830 (m); ^{1}H NMR (400 MHz, $CDCl_3$) δ 0.98 (dd collapsed to t, *J* = 7.2 Hz, 3H), 1.12 (dd collapsed to t, *J* = 7.2 Hz, 3H), 2.65, 2.90 (overlapped ABq, *J* = 17.1 Hz, the upper half further split into d, *J* = 2.8 Hz and the lower half further split into d, *J* = 14.3 Hz, 2H), 2.94, 3.04 (ABq, *J* = 19.1 Hz, the upper half further split into d, *J* = 9.9 Hz, and the lower half further split into d, *J* = 6.4 Hz, 2H), 3.65, 3.80 (ABqq, *J* =

10.7, 7.2 Hz, 2H), 3.90 (dd, *J* = 9.9, 6.4 Hz, 1H), 4.00 (dq, *J* = 10.7, 7.2 Hz, 1H), 4.20 (dq, *J* = 10.7, 7.2 Hz, 1H), 4.84 (s, 1H), 5.44 (dd, *J* = 14.3, 2.8 Hz, 1H), 7.17-7.25 (m, 6H), 7.25-7.30 (m, 1H), 7.35-7.43 (m, 6H), 7.46 (s, 1H); ^{13}C NMR (100 MHz, $CDCl_3$) δ 13.7, 13.9, 33.8, 40.8, 43.2, 43.5, 61.3, 61.5, 62.1, 80.1, 113.5, 122.3, 126.4, 127.5, 127.8, 128.3, 129.0 (× 2), 129.7, 130.2, 130.6, 132.8, 138.2, 139.5, 143.1, 168.9, 169.5, 170.1, 189.6; HRMS (ES+) calcd for $C_{33}H_{31}{}^{79}BrO_6Na$ (MNa^+) 625.1196, found 625.1200.

Diethyl 3-cinnamoyl-4-oxo-6-phenyl-2-*p*-tolylcyclohexane-1,1-dicarboxylate (3e)

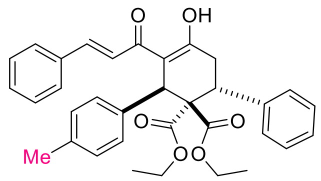

3e

Yellow oil; 46% (25 mg), >95:5; R_f: 0.5 (15% ethyl acetate in pet ether; IR (KBr, cm^{-1}) 2982 (m), 2934 (w), 1753 (s), 1732 (s), 1629 (m), 1449 (w), 1368 (w), 1244 (s), 1038 (w), 756 (w); ^{1}H NMR (400 MHz, $CDCl_3$) δ 0.89 (dd collapsed to t, *J* = 7.2 Hz, 3H), 1.15 (dd collapsed to t, *J* = 7.2 Hz, 3H), 2.29 (s, 3H), 2.91, 3.00 (ABq, *J* = 19.4 Hz, upper half further split into d, *J* = 6.6 Hz and lower half further split into d, 11.9 Hz, 2H), 3.70, 3.75 (ABqq, *J* = 10.8, 7.2 Hz, 2H), 3.90 (dd, *J* = 11.8, 6.6 Hz, 1H), 4.10 (dq, *J* = 10.8, 7.2 Hz, 1H), 4.28 (dq, *J* = 10.8, 7.2 Hz, 1H), 4.85 (s, 1H), 6.80 (d, *J* = 15.5 Hz, 1H), 7.13, 7.15 (ABq, *J* = 8.6 Hz, 4H), 7.16-7.21 (m, 3H), 7.24-7.27 (m, 2H), 7.30-7.36 (m, 5H), 7.60 (d, *J* = 15.5 Hz, 1H), 16.97 (s, 1H); ^{13}C NMR (100 MHz, $CDCl_3$) δ 13.6, 14.0, 21.3, 37.6, 39.2, 46.9, 61.2, 61.6, 63.2, 109.6, 120.7, 127.2, 127.5, 128.4, 129.0, 129.4, 129.8, 130.4, 130.7, 135.3, 137.6, 138.1, 139.4, 142.7, 168.8, 169.8, 185.1, 186.8; HRMS (ES+) calcd for $C_{34}H_{34}O_6Na$ (MNa^+) 561.2248, found 561.2249.

Diethyl 3-cinnamoyl-2-(naphthalen-1-yl)-4-oxo-6-phenylcyclohexane-1,1-dicarboxylate (3f)

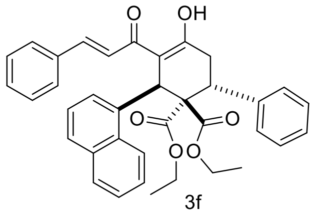

3f

Yellow solid; yield 73% (42 mg), dr > 95:5; R_f: 0.4 (15% ethyl acetate in pet ether; mp 146 °C; IR (KBr, cm^{-1}) 3461 (br w), 3028 (w), 2924 (s), 2852 (w), 1727 (vs), 1629 (m), 1597 (m), 1576 (m), 1265 (m), 1240 (s), 1205 (m), 776 (s); ^{1}H NMR (500 MHz, $CDCl_3$) δ 0.35 (dd collapsed to t, *J* = 7.1 Hz, 1H), 1.27 (dd collapsed to t, J = 7.1 Hz, 1H), 2.91, 3.12 (ABdq, J = 10.7 Hz, 7.20 Hz, 2H), 2.99 (dd, 19. 3, 6.0 Hz, 1H), 3.29 (dd, J = 19.4, 13.7 Hz, 1H), 4.06 (dd, *J* = 12.6, 6.0 Hz, 1H), 4.28, 4.39 (ABdq, *J* = 14.2, 10.8 Hz, 7.0 Hz, 2H), 6.1 (s, 1H), 6.58 (d, *J* = 15.5 Hz, 1H), 6.8 (d, *J* = 7.5 Hz, 2H), 7.08 (t, J = 7.8 Hz, 2H), 7.21-7.15 (m, 4H), 7.37 (d, *J* = 6.1 Hz, 2H), 7.36 (d, *J* = 6.5 Hz, 2H), 7.54 – 7.45 (m, 3H), 7.57 (t, J = 7.5 Hz, 1H), 7.67 (t, *J* = 7.2 Hz, 1H), 7.79 (d, *J* = 8.0 Hz, 1H), 7.92 (d, *J* = 8.1 Hz, 1H), 8.39 (d, *J* = 8.6 Hz, 1H), 16.85 (s, 1H); Confirmed by ^{1}H-^{1}H COSY experiment; ^{13}C NMR (125 MHz, $CDCl_3$) δ 12.8, 14.2, 36.7, 40.1, 41.0, 61.1, 62.0, 63.0, 110.2, 120.9, 122.4, 125.7, 125.8, 127.1, 127.3, 127.7, 128.4, 128.5, 128.8, 129.2, 129.7, 130.3, 130.8, 132.7, 133.9, 134.9, 138.0, 139.0, 143.2, 168.8, 169.8, 184.5, 188.0; HRMS (ES+) calcd for $C_{37}H_{34}O_6Na$ (MNa^+) 597.2248, found 597.2259.

Diethyl 3-cinnamoyl-2-(furan-2-yl)-4-oxo-6-phenylcyclohexane-1,1-dicarboxylate (3g)

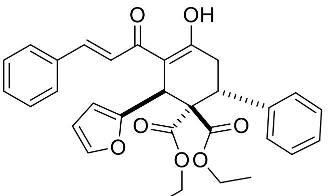

3g

Yellow solid; yield 54% (28 mg), dr > 95:5; R_f: 0.5 (15% ethyl acetate in pet ether; mp 119 °C; IR (KBr, cm^{-1}) 3450 (br w), 2982 (m), 2927 (m), 1730 (vs), 1629 (m), 1601 (m), 1244 (s), 1205 (m), 757 (vs), 701 (m); ^{1}H NMR (500 MHz, $CDCl_3$) δ 1.02 (dd collapsed to t, *J* = 7.5 Hz, 3H), 1.16 (dd collapsed to t, *J* = 7.0 Hz, 3H), 2.90, 3.40 (ABq, *J* = 19.0 Hz, upper half further split into d, *J* = 6 Hz and lower half further split into d, *J* = 11.9 Hz, 2H), 3.91 (q, *J* = 7.0 Hz, 2H), 4.10 (dd, *J* = 12.0 Hz,

7.0 Hz, 1H), 4.10, 4.26 (ABqq, J = 10.5, 7.0 Hz, 2H), 5.01 (s, 1H), 6.23 (d, J = 3.0 Hz, 1H), 6.36 (dd, J = 3.0 Hz, 2.0, Hz, 1H), 6.83 (d, J = 15.5 Hz, 1H), 7.22-7.26 (m, 3H), 7.34 (d, J = 6.7 Hz, 2H), 7.37 (t, 3.5 Hz, 3H), 7.40 (Unresolved d, 1.5 Hz, 1H) 7.45 (Unresolved dd, J = 7.0 Hz, 3.5 Hz, 2H), 7.72 (d, J = 15.5 Hz, 1H); ^{13}C NMR (125 MHz, $CDCl_3$) δ 13.8, 14.0, 37.5, 40.1, 41.4, 61.6, 61.8, 62.2, 107.5, 110.1, 111.1, 119.7, 127.3, 127.6, 128.5, 129.1, 130.5, 130.6, 135.2, 139.4, 142.5, 143.2, 154.4, 168.9, 169.2, 184.6, 187.7; HRMS (ES+) calcd for $C_{31}H_{30}O_7K$ (MK^+) 553.1623, found 553.1625.

(*E*)-Diethyl 2-(4-chlorophenyl)-4-oxo-6-*p*-tolyl-3-(3-*p*-tolylacryloyl)cyclohexane-1,1-dicarboxylate (3i)

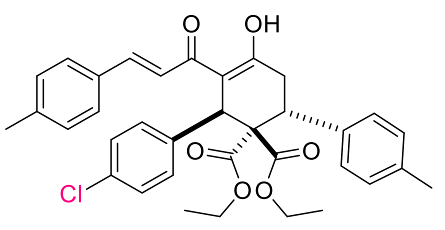

3i

Yellow oil; yield 43% (25 mg); dr > 95:5; R_f: 0.5 (10% ethyl acetate in pet ether; IR (KBr, cm^{-1}) 2983 (m), 2926 (m), 1727 (vs), 1627 (s), 1606 (m), 1411 (m), 1241 (vs), 1181 (s), 1093 (m), 815 (m), 757 (vs); ^{1}H NMR (400 MHz, $CDCl_3$) δ 0.94 (dd collapsed to t, J = 7.2 Hz, 3H), 1.19 (dd collapsed to t, J = 7.2 Hz, 3H), 2.29 (s, 3H), 2.36 (s, 3H), 2.95, 3.01 (ABq, J = 19.3 Hz, upper half further split into d, J = 7.4 Hz and lower half further split into d, 10.5 Hz, 2H), 3.75 (ABqq, J = 10.8, 7.2 Hz, 2H), 3.80 (dd, J = 10.5, 7.4 Hz, 1H), 4.12 (q, J = 10.8, 7.2 Hz, 1H), 4.29 (dq, J = 10.8, 7.2 Hz, 1H), 4.90 (s, 1H), 6.70 (d, J = 15.5 Hz, 1H), 7.02 (d, J = 8.1 Hz, 2H), 7.15 (d, J = 8.1 Hz, 4H), 7.16, 7.26 (ABq, J = 8.0 Hz, 4H), 7.24, 7.34 (ABq, J = 8.5 Hz, 4H), 7.63 (d, J = 15.5 Hz, 1H), 17.08 (s, 1H); ^{13}C NMR (100 MHz, $CDCl_3$) δ 13.7, 14.0, 21.2, 21.7, 37.5, 39.0, 46.4, 61.3, 61.8, 63.1, 108.9, 119.2, 128.3, 128.5, 128.8, 129.8, 130.4, 131.2, 132.4, 133.8, 136.1, 137.0, 140.1, 141.2, 143.3, 168.9, 169.6, 185.5, 186.6; HRMS (ES+) calcd for $C_{35}H_{35}{}^{35}ClO_6Na$ (MNa^+) 609.2014, found 609.2021.

(*E*)-Diethyl 6-(4-bromophenyl)-3-(3-(4-bromophenyl)acryloyl)-2-(4-chlorophenyl)-4-oxocyclohexane-1,1-dicarboxylate (3j)

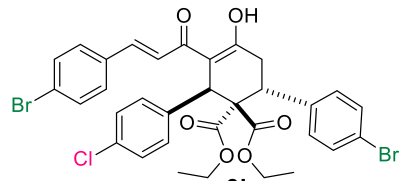

3j

Yellow solid; yield 35% (25 mg); dr > 95:5; R_f: 0.4 (10% ethyl acetate in pet ether; mp 97 °C; IR (KBr, cm^{-1}) 2929 (m), 2854 (w), 1725 (vs), 1627 (w), 1488 (m), 1245 (m), 1010 (m), 817 (m), 757 (s), 738 (m); ^{1}H NMR (400 MHz, $CDCl_3$) δ 0.96 (dd collapsed to t, J = 7.1 Hz, 3H), 1.19 (dd collapsed to t, J = 7.1 Hz, 3H), 2.91, 2.96 (ABq, J = 19.4 Hz, upper half further split into d, J = 7.4 Hz and lower half further split into d, 11.0 Hz, 2H), 3.74, 3.81 (overlapped ABqq, J = 10.8, 7.1 Hz, 2H), 3.78 (overlapped dd, J = 11.0, 7.4 Hz, 1H), 4.14, 4.30 (ABqq, J = 10.8, 7.1 Hz, 2H), 4.84 (s, 1H), 6.69 (d, J = 15.4 Hz, 1H), 7.15 (d, J = 8.6 Hz, 2H), 7.20 (d, J = 8.4 Hz, 4H), 7.33 (d, J = 8.4 Hz, 2H), 7.36 (d, J = 8.6 Hz, 2H), 7.48 (d, J = 8.4 Hz, 2H), 7.56 (d, J = 15.4 Hz, 1H), 16.93 (s, 1H); ^{13}C NMR (100 MHz, $CDCl_3$) δ 13.7, 14.1, 37.3, 38.8, 46.5, 61.6, 62.0, 62.9, 109.0, 120.7, 121.5, 125.0, 129.0, 129.8, 130.7, 131.1, 132.4, 132.5, 133.9, 134.1, 138.0, 139.6, 142.0, 168.5, 169.3, 184.9, 186.6; HRMS (ES+) calcd for $C_{33}H_{29}{}^{79}Br_2{}^{35}ClO_6Na$ (MNa^+) 736.9912, found 736.9883.

Diethyl-2-(4-chlorophenyl)-4-hydroxy-6-(naphthalen-1-yl)-3-((E)-3-(naphthalen-1 yl)acryloyl)cyclohex-3-ene-1,1-dicarboxylate (3k)

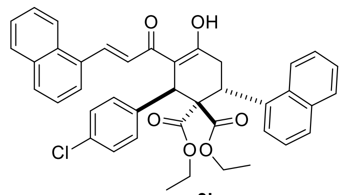

3k

Yellow solid; yield 44% (25 mg); dr .95:5; R_f: 0.3 (10% ethyl acetate in pet ether;IR (KBr, cm^{-1}) 3124 (br, s), 2982 (s), 1743 (s), 1731 (vs), 1705 (s), 1625 (m), 1601 (s), 1512 (vs), 1403 (s), 1284 (s), 1257 (vs),

1229 (vs), 1206 (s), 1174 (vs), 1032 (m), 831 (s); ^{1}H NMR (400 MHz, $CDCl_3$) δ 0.52 (dd collapsed to t, J = 7.2 Hz), 1.06 (dd collapsed to t, J = 7.2 Hz), 3.09, 3.35 (ABqd, J = 19.2 Hz, upper half further split into d, J = 10.0 Hz, and lower half further split into d, J = 6.4 Hz, 2H), 3.47, 3.51 (ABqq, J = 10.8, 7.2 Hz), 4.10, 4.23 (ABqq, J = 10.8, 7.2 Hz, 2H), 4.89 (dd, J = 10.0, 6.4 Hz, 1H), 5.28 (s, 1H), 6.91 (d, J = 15.4 Hz, 1H), 7.36 (d, J = 7.7 Hz, 1H), 7.39-7.40 (m, 1H), 7.43 (d, J = 7.2 Hz, 2H), 7.45–7.48 (m, 5H), 7.52-7.55 (m, 2H), 7.60 (d, J = 7.2 Hz, 1H), 7.72 (d, J = 8.0 Hz, 1H), 7.81-7.83 (m, 1H), 7.86-7.90 (m, 3H), 8.09 (d, J = 8.0 Hz, 1H), 8.52 (d, J = 15.4 Hz, 1H); Confirmed by ^{1}H-^{1}H COSY experiment; ^{13}C NMR (125 MHz, $CDCl_3$) δ 13.1, 13.8, 34.1, 38.4, 46.4, 61.1, 61.8, 62.9, 108.9, 122.6, 123.0, 123.5, 125.0, 125.3 (× 2), 125.5, 125.7, 126.2, 126.3, 127.0, 127.8, 128.7, 128.8, 129.1, 130.8, 131.4, 131.5, 132.2, 132.4, 133.7, 133.8 (× 2), 136.3, 140.1, 140.2, 169.1, 169.8, 184.3, 184.4; HRMS (ES+) calcd for $C_{41}H_{35}{}^{35}ClO_6K$ (MK^+) 697.1754, found 697.1752.

(*E*)-Diethyl 2-(4-fluorophenyl)-6-(4-methoxyphenyl)-3-(3-(4-methoxyphenyl)acryloyl)-4-oxocyclohexane-1,1-dicarboxylate (3l)

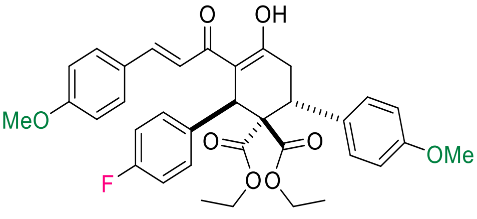

3l

Yellow solid; yield 54% (32 mg), dr > 95:5; R_f: 0.3 (20% ethyl acetate in pet ether; mp 143 °C; IR (KBr, cm^{-1}) 3301 (br w), 2935 (m), 2837 (w), 1725 (s), 1629 (m), 1611 (m), 1513 (vs), 1420 (m), 1252 (vs), 1159 (s), 1034 (m), 830 (m), 756 (vs); ^{1}H NMR (500 MHz, $CDCl_3$) δ 0.95 (dd collapsed to t, J = 7.2 Hz, 3H), 1.19 (dd collapsed to t, J = 7.2 Hz, 3H), 2.93, 2.98 (ABq, J = 19.3 Hz, upper half further split into d, J = 7.2 Hz and lower half further split into d, 10.8 Hz, 2H), 3.71-3.81 (overlapped m, 3H), 3.76 (s, 3H), 3.82 (s, 3H), 4.12 (dq, J = 10.8, 7.2 Hz, 1H), 4.29 (dq, J = 10.8, 7.2 Hz, 1H),4.90 (s, 1H), 6.61 (d, J = 15.4 Hz, 1H), 6.75 (d, J = 8.8 Hz, 2H), 6.86 (d, J = 8.7 Hz, 2H), 7.05 (t, J = 8.5 Hz, 2H), 7.19 (d, J = 8.8 Hz, 2H), 7.28 (dd, J = 8.5, 5.4 Hz, 2H), 7.31 (d, J = 8.7 Hz, 2H), 7.61 (d, J = 15.4 Hz, 1H), 17.12 (s, 1H); ^{13}C NMR (125 MHz, $CDCl_3$) δ 13.7, 14.0, 37.4, 38.5, 46.3, 55.3, 55.6, 61.3, 61.7, 63.3, 108.9, 112.9, 114.6, 115.5 (d, $J_{C\text{-}F}$ = 20.2 Hz), 118.0, 127.9, 130.2, 131.1, 131.5 (d, $J_{C\text{-}F}$ = 8.2 Hz), 131.7, 137.3 (d, $J_{C\text{-}F}$ = 2.8 Hz), 143.0, 158.7, 161.7, 162.3 (d, $J_{C\text{-}F}$ = 247.8 Hz), 168.9, 169.7, 185.7, 186.0; ^{19}F NMR (470 MHz, $CDCl_3$) δ -114.40; HRMS (ES+) calcd for $C_{35}H_{35}FO_8Na$ (MNa^+) 625.2208, found 625.2207.

(*E*)-Diethyl 6-(4-methoxyphenyl)-3-(3-(4-methoxyphenyl)acryloyl)-4-oxo-2-*p*-tolylcyclohexane-1,1-dicarboxylate (3m)

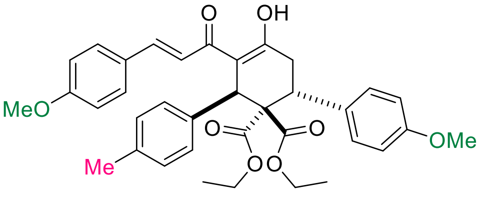

3m

Yellow solid; mp 151 °C; yield 42% (25 mg), dr > 95:5; R_f: 0.3 (15% ethyl acetate in pet ether); IR (KBr, cm^{-1}) 3426 (w), 2955 (w), 2928 (m), 2837 (w), 1726 (vs), 1625 (m), 1602 (s), 1512 (vs), 1302 (m), 1172 (m), 1034 (m), 830 (m), 757 (s); ^{1}H NMR (500 MHz, $CDCl_3$) δ 0.95 (dd collapsed to t, J = 7.2 Hz, 3H), 1.19 (dd collapsed to t, J = 7.2 Hz, 3H), 2.32 (s, 3H), 2.88, 2.96 (ABq, J = 19.4 Hz, upper half further split into d, J = 6.5 Hz and lower half further split into d, 12.0 Hz, 2H), 3.72-3.88 (overlapped m, 3H), 3.76 (s, 3H), 3.82 (s, 3H), 4.11 (dq, J = 10.8, 7.2 Hz, 1H), 4.30 (dq, J = 10.8, 7.2 Hz, 1H), 4.84 (s, 1H), 6.68 (d, J = 15.4 Hz, 1H), 6.74 (d, J = 8.8 Hz, 2H), 6.86 (d, J = 8.7 Hz, 2H), 7.14-7.16 (ABq collapsed to unresolved m, 4H), 7.20 (d, J = 8.8 Hz, 2H), 7.32 (d, J = 8.7 Hz, 2H), 7.60 (d, J = 15.4 Hz, 1H), 17.10 (s, 1H); ^{13}C NMR (125 MHz, $CDCl_3$) δ 13.7, 14.1, 21.3, 37.5, 38.3, 46.9, 55.3, 55.6, 61.1, 61.6, 63.3, 109.3, 112.8, 114.5, 118.3, 128.1, 129.3, 129.8, 130.2, 131.4, 131.9, 137.5, 138.2, 142.6, 158.6, 161.6, 168.9, 169.9, 185.8, 185.9; HRMS (ES+) calcd for $C_{36}H_{38}O_8$ (MNa^+) 621.2459, found 621.2481.

Diethyl 2,7-bis(4-methoxyphenyl)-4-oxo-5-*p*-tolyl-3,4,7,8-tetrahydro-2*H*-chromene-6,6(5*H*)-dicarboxylate (4m)

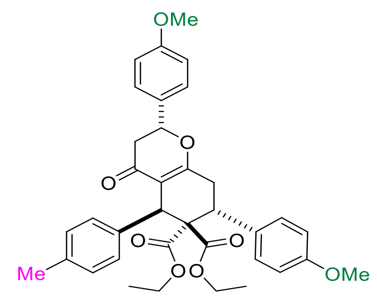

4m

Yellow oil; yield 16% (10 mg), dr 70:30; R_f: 0.3 (20% ethyl acetate in pet ether); The major isomer was isolated in pure form by silica gel column chromatography; IR (KBr, cm^{-1}) 2957 (m), 2933 (m), 2927 (m), 2838 (w), 1725 (vs), 1668 (m), 1628 (s), 1613 (s), 1514 (vs), 1406 (m), 1249 (vs), 1181 (s), 1157 (m), 1034 (m), 830 (m), 756 (vs); ^1H NMR (400 MHz, $CDCl_3$) δ 0.99 (dd collapsed to t, *J* = 7.1 Hz, 3H), 1.14 (dd collapsed to t, *J* = 7.1 Hz, 3H), 2.32 (s, 3H), 2.58 (dd, *J* = 17.0, 2.9 Hz, 1H), 2.94 (dd, *J* = 17.0, 14.3 Hz, 1H), 2.83, 2.89 (ABq, J = 19.0 Hz, the upper half was further split into d, *J* = 6.9 Hz and the lower half was further split into d *J* = 10.5 Hz, 2H), 3.64, 3.79 (ABqq, *J* = 10.7, 7.1 Hz, 2H), 3.76 (s, 3H), 3.82 (s, 3H), 3.93 (dd, *J* = 10.5, 6.9 Hz, 1H), 4.01 (dq, *J* = 10.7, 7.1 Hz, 1H), 4.24 (dq, *J* = 10.7, 7.1 Hz, 1H), 4.80 (s, 1H), 5.34 (dd, *J* = 14.3, 2.9 Hz, 1H), 6.74 (d, *J* = 8.6 Hz, 2H), 6.93 (d, *J* = 8.6 Hz, 2H), 7.11 (d, *J* = 8.2 Hz, 2H), 7.18 (ABq, *J* = 7.9 Hz, 4H), 7.34 (d, *J* = 8.2 Hz, 2H); ^{13}C NMR (100 MHz, $CDCl_3$) δ 13.7, 14.0, 21.3, 34.0, 39.5, 43.2, 43.7, 55.3, 55.5, 61.0, 61.2, 62.3, 79.8, 112.9, 114.1, 114.3, 128.0, 129.0, 129.6, 130.5, 131.6 (× 2), 137.1, 137.5, 158.7, 160.2, 169.0, 169.4, 169.9, 190.0; HRMS (ES+) calcd for $C_{36}H_{39}O_8$ (MNa^+) 599.2639, found 599.2633.

(*E*)-Diethyl 6-(furan-2-yl)-3-(3-(furan-2-yl)acryloyl)-4-oxo-2-phenylcyclohexane-1,1-dicarboxylate (3n)

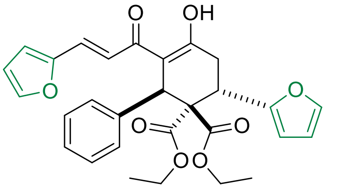

3n

Yellow oil; yield 56% (28 mg), dr > 95:5; R_f: 0.3 (15% ethyl acetate in pet ether); IR (KBr, cm^{-1}) 3449 (br w), 2984 (w), 2929 (w), 1729 (vs), 1628 (m), 1450 (w), 1244 (vs), 1206 (s), 757 (vs), 701 (m); ^1H NMR (400 MHz, $CDCl_3$) δ 0.98 (dd collapsed to t, *J* = 7.2 Hz, 3H), 1.12 (dd collapsed to t, *J* = 7.2 Hz, 3H), 2.98, 3.07 (ABq, *J* = 19.8, upper half further split into d, *J* = 6.8 Hz and lower half further split into d, 12.0 Hz, 2H), 3.82, 3.88 (ABqq, *J* = 10.7, 7.2 Hz, 2H), 4.06 (overlapped dd, *J* = 12.0, 6.8 Hz, 1H), 4.08, 4.18 (ABqq, *J* = 10.7, 7.2 Hz, 2H), 4.87 (s, 1H), 6.19 (d, *J* = 3.3 Hz, 1H), 6.26 (dd, *J* = 3.3, 1.8 Hz, 1H), 6.43 (dd, *J* = 3.4, 1.5 Hz, 1H), 6.54 (d, *J* = 3.4 Hz, 1H), 6.70 (d, *J* = 15.2 Hz, 1H), 7.21 (d, *J* = 7.3 Hz, 2H), 7.25-7.27 (overlapped m, 2H), 7.32 (t, *J* = 7.3 Hz, 2H), 7.38 (d, *J* = 15.2 Hz, 1H), 7.44 (d, *J* = 1.5 Hz, 1H), 17.00 (s, 1H); ^{13}C NMR (100 MHz, $CDCl_3$) δ 13.7, 13.9, 32.7, 34.2, 46.4, 61.4, 61.9, 62.7, 108.7, 109.0, 110.6, 112.7, 115.9, 117.9, 127.8, 128.7, 129.2, 129.7, 140.8, 141.0, 145.2, 151.9, 153.4, 168.8, 169.1, 185.1, 185.2; HRMS (ES+) calcd for $C_{29}H_{28}O_8Na$ (MNa^+) 527.1676, found 527.1682.

References

1. Yin, X.-G.; Liu, X.-Y.; Hu, Z.-P.; Yan, M. *Org. Biomol. Chem.* **2012**, *10*, 1506.
2. Wang, J.; Zhou, Y.; Zhang, L.; Li, Z.; Chen, X.; Liu, H. *Org. Lett.* **2023**, *15*, 1508.
