# Supplementary material for: Diastereoselective synthesis of highly substituted cyclohexanones and tetrahydrochromene-4-ones via conjugate addition of curcumins to arylidenemalonates
Source: Beilstein J Org Chem. 2024 Aug 15;20:2016–23. doi: 10.3762/bjoc.20.177 (PMC11331540; doi:10.3762/bjoc.20.177)
Supplement: File 2 — Copies of NMR spectra of all new compounds. [file Beilstein_J_Org_Chem-20-2016-s002.pdf]

BEILSTEIN JOURNAL OF ORGANIC CHEMISTRY

Supporting Information

for

Diastereoselective synthesis of highly substituted cyclohexanones and tetrahydrochromene-4-ones via conjugate addition of curcumins to arylidenemalonates

Deepa Nair, Abhishek Tiwari, Banamali Laha and Irishi N. N. Namboothiri

Beilstein J. Org. Chem. **2024,** *20,* 2016–2023. doi:10.3762/bjoc.20.177

Copies of NMR spectra of all new compounds

License and Terms: This is a supporting information file under the terms of the Creative Commons Attribution License (https://creativecommons.org/licenses/by/4.0). Please note that the reuse, redistribution and reproduction in particular requires that the author(s) and source are credited and that individual graphics may be subject to special legal provisions.
The license is subject to the *Beilstein Journal of Organic Chemistry* terms and conditions: (https://www.beilstein-journals.org/bjoc/terms)

Entry	Table of contents	Page
1	**Fig S1. ^{1}H NMR Spectrum of 3a**	S3
2	**Fig S2. ^{13}C NMR Spectrum of 3a**	S4
3	**Fig S3. ^{1}H NMR Spectrum of 4a**	S5
4	**Fig S4. ^{13}C NMR Spectrum of 4a**	S6
5	**Fig S5. ^{1}H -^{1}H COSY NMR Spectrum of 4a**	S7
6	**Fig S6. ^{1}H -^{1}H NOESY NMR Spectrum of 4a**	S8
5	**Fig S7. ^{1}H NMR Spectrum of 3b**	S9
6	**Fig S8 ^{1}H -^{1}H COSY NMR Spectrum of 3b**	S10
7	**Fig S9 ^{1}H -^{1}H COSY NMR Spectrum (expansion) of 3b**	S11
8	**Fig S10. ^{1}H -^{1}H NOESY NMR Spectrum of 3b**	S12
9	**Fig S11. ^{1}H -^{1}H NOESY NMR Spectrum (expansion) of 3b**	S13
10	**Fig S12. ^{13}C NMR Spectrum of 3b**	S14
11	**Fig S13. ^{13}C NMR Spectrum (expansion) of 3b**	S15
12	**Fig S14. ^{13}C-APT Spectrum of 3b**	S16
13	**Fig S15. ^{19}F NMR Spectrum of 3b**	S17
14	**Fig S16. ^{1}H NMR Spectrum of 4b**	S18
15	**Fig S17. ^{13}C NMR Spectrum of 4b**	S19
16	**Fig S18. ^{13}C NMR Spectrum (expansion) of 4b**	S20
17	**Fig S19. ^{13}C-APT Spectrum of 4b**	S21
18	**Fig S20. ^{19}F NMR Spectrum of 4b**	S22
19	**Fig S21. ^{1}H NMR Spectrum of 3c**	S23
20	**Fig S22. ^{13}C NMR Spectrum of 3c**	S24
21	**Fig S23. ^{1}H NMR Spectrum of 4c**	S25
22	**Fig S24. ^{13}C NMR Spectrum of 4c**	S26
23	**Fig S25. ^{1}H NMR Spectrum of 3e**	S27
24	**Fig S26. ^{13}C NMR Spectrum of 3e**	S28
25	**Fig S27. ^{1}H NMR Spectrum of 3f**	S29
26	**Fig S28. ^{1}H-^{1}H COSY NMR Spectrum of 3f**	S30
27	**Fig S29. ^{13}C NMR Spectrum of 3f**	S31
28	**Fig S30. ^{13}C NMR Spectrum (exapansion of 3f**	S32
29	**Fig S31. ^{13}C-APT Spectrum of 3f**	S33
30	**Fig S32. ^{1}H NMR Spectrum of 3g**	S34
31	**Fig S33. ^{13}C NMR Spectrum of 3g**	S35

32	**Fig S34. ^{1}H NMR Spectrum of 3i**	S36
33	**Fig S35. ^{13}C NMR Spectrum of 3i**	S37
34	**Fig S36. ^{1}H NMR Spectrum of 3j**	S38
35	**Fig S37. ^{13}C NMR Spectrum of 3j**	S39
36	**Fig S38. ^{1}H NMR Spectrum of 3k**	S40
37	**Fig S39. ^{1}H-^{1}H COSY NMR spectrum of 3k**	S41
38	**Fig S40. ^{13}C NMR Spectrum of 3k**	S42
39	**Fig S41. ^{13}C NMR Spectrum (expansion) of 3k**	S43
40	**Fig S42. ^{1}H NMR Spectrum of 3l**	S44
41	**Fig S43. ^{13}C NMR Spectrum of 3l**	S45
42	**Fig S44. ^{13}C-APT Spectrum of 3l**	S46
43	**Fig S45. ^{19}F NMR spectrum of 3l**	S47
44	**Fig S46. ^{1}H NMR Spectrum of 3m**	S48
45	**Fig S47. ^{13}C NMR Spectrum of 3m**	S49
46	**Fig S48. ^{1}H NMR Spectrum of 4m**	S50
47	**Fig S49. ^{13}C NMR Spectrum of 4m**	S51
48	**Fig S50. ^{1}H NMR Spectrum of 3n**	S52
49	**Fig S51. ^{13}C NMR Spectrum of 3n**	S53

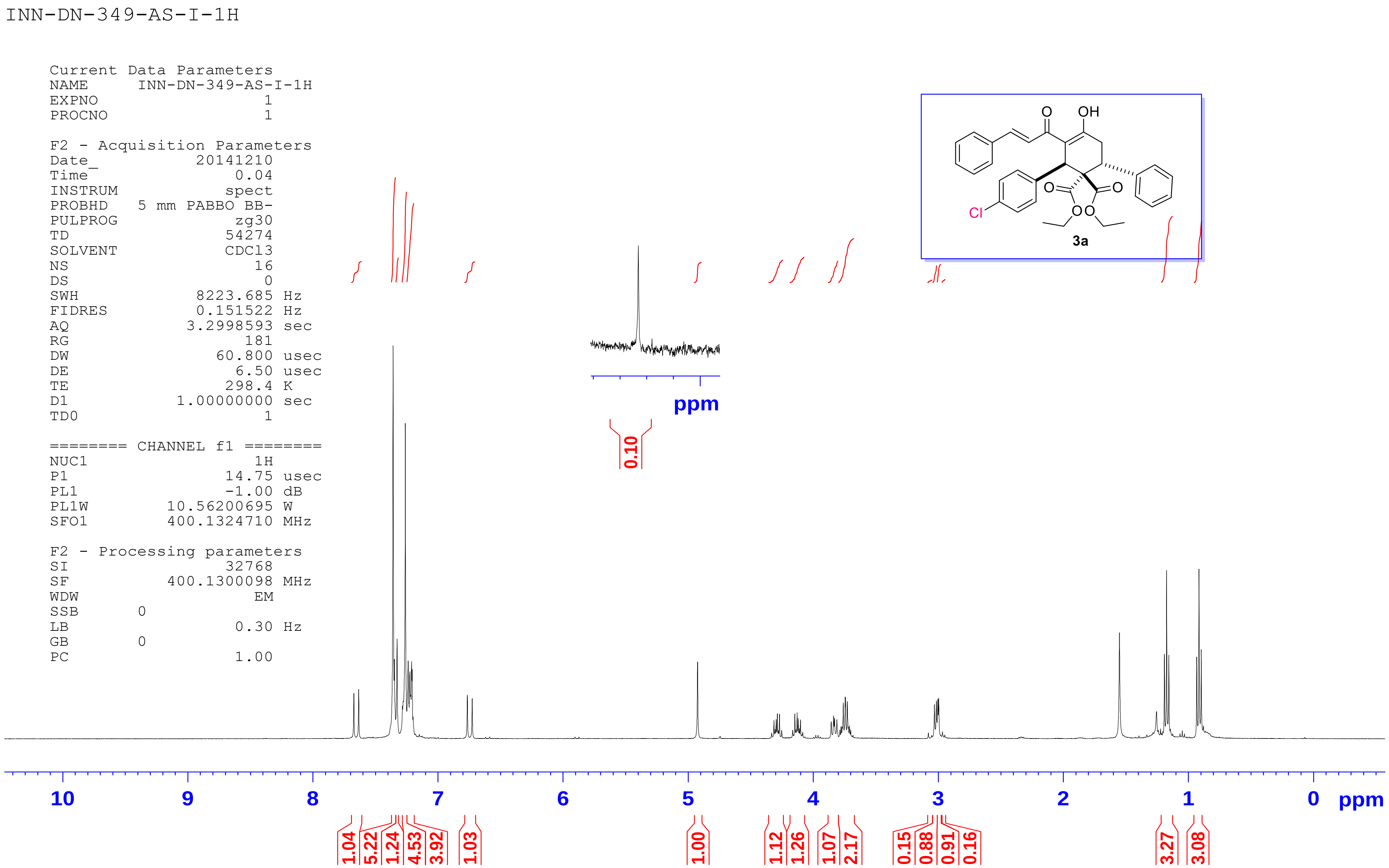

Fig S1. ^{1}**H NMR Spectrum of 3a**

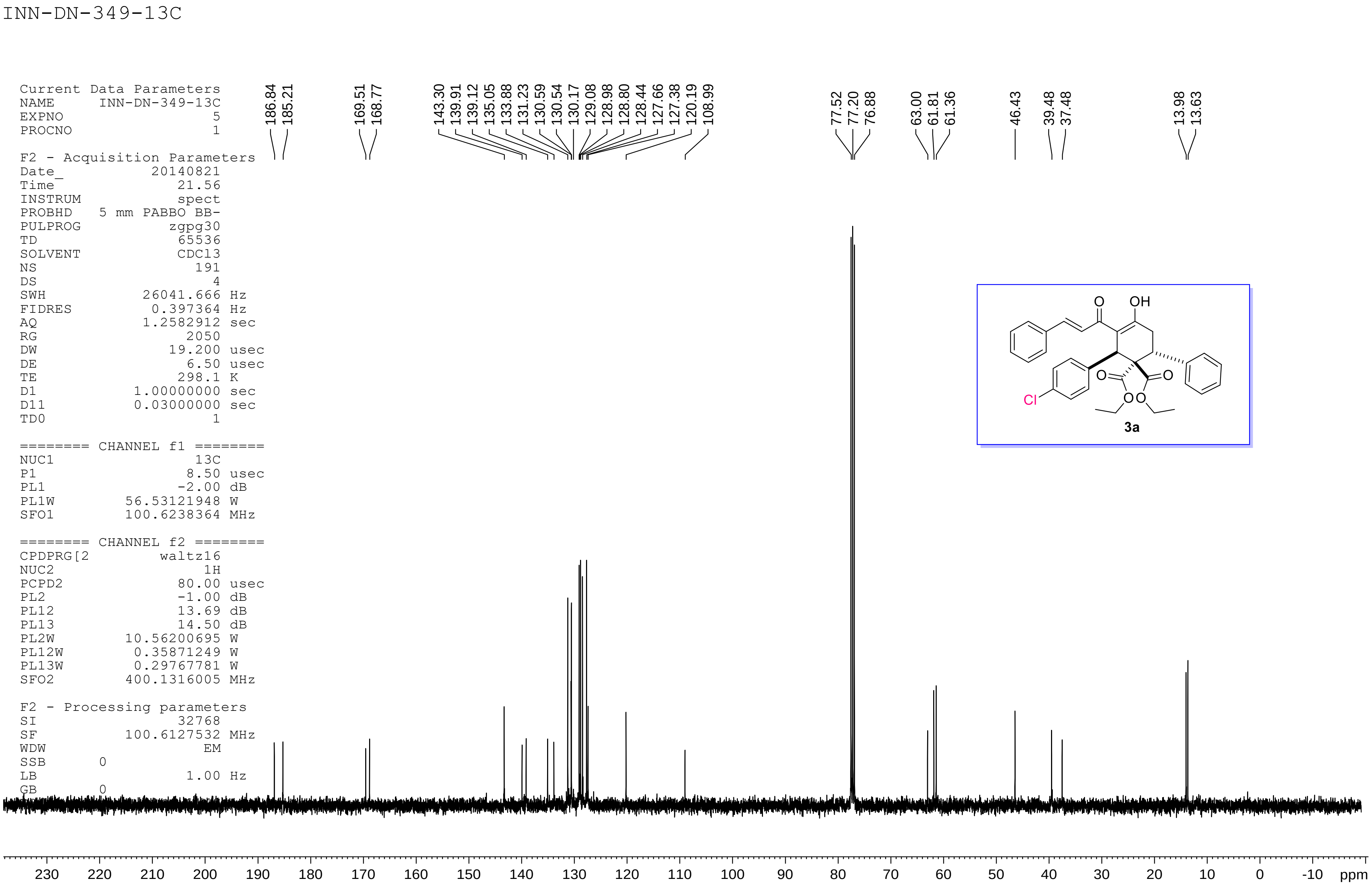

Fig S2. ^{13}C NMR Spectrum of 3a

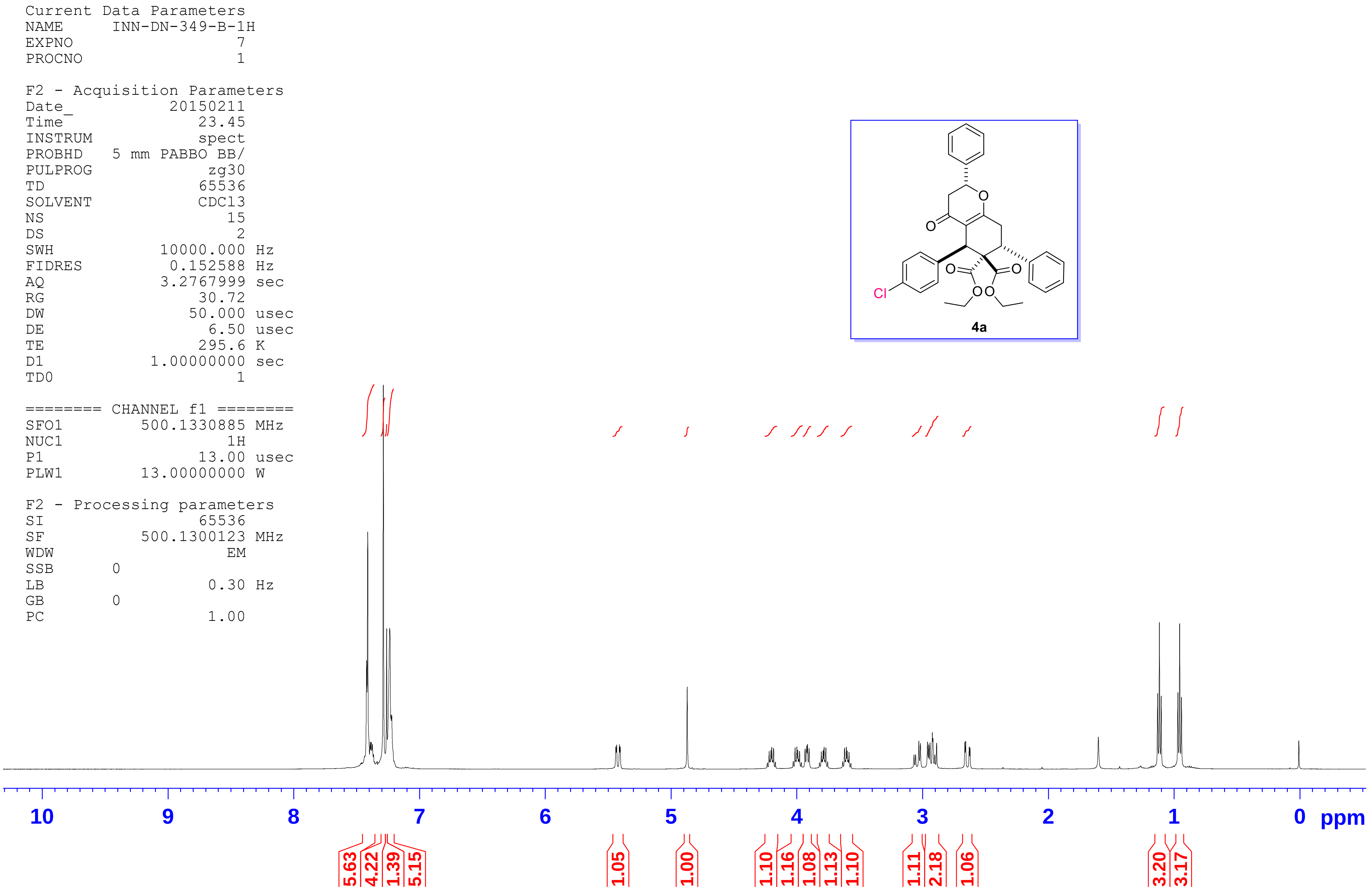

Fig S3. ^{1}H NMR spectrum of 4a

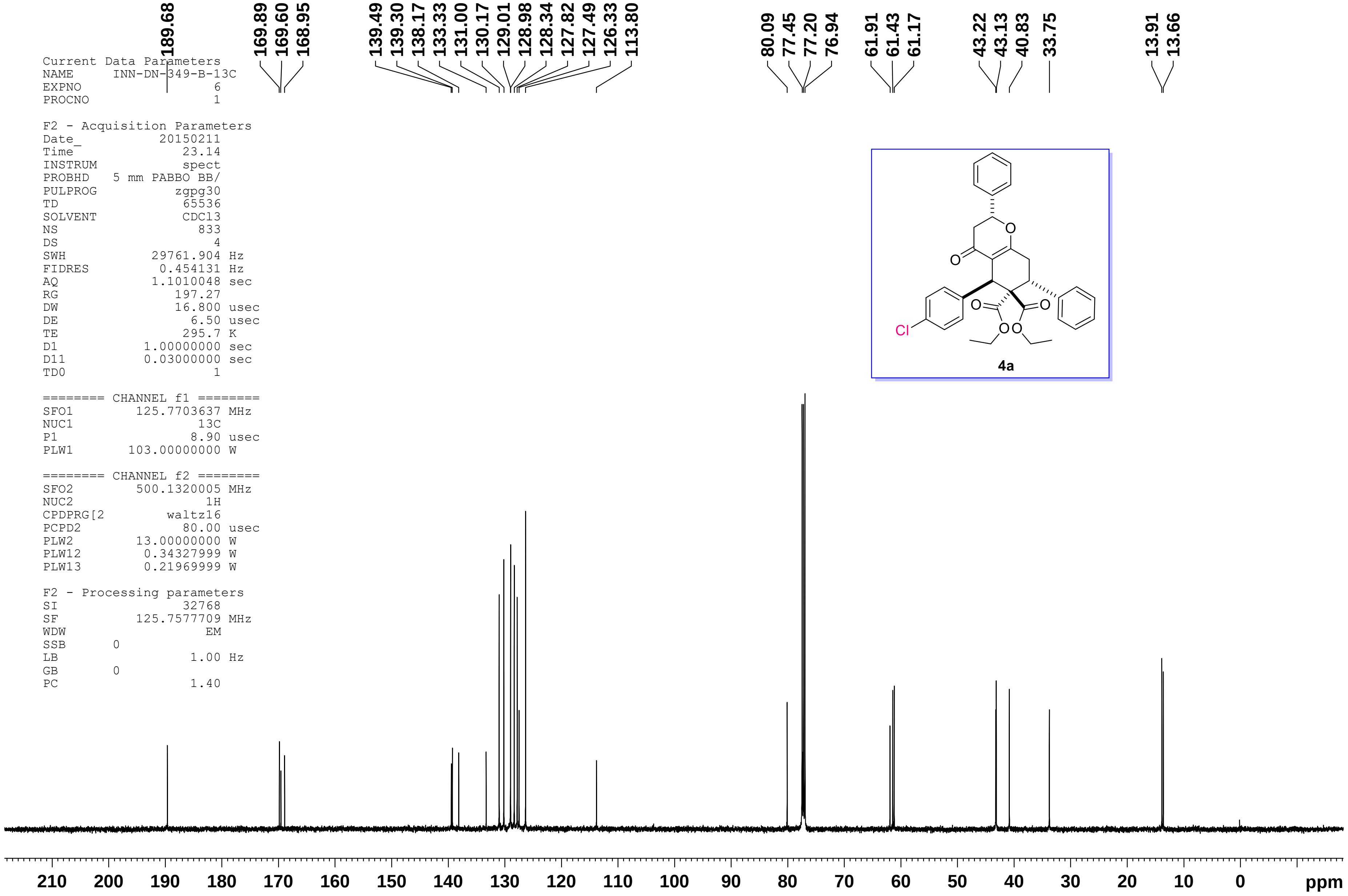

Fig S4. ^{13}C NMR spectrum of 4a

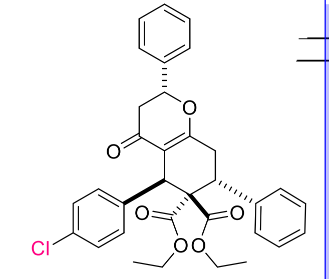

4a

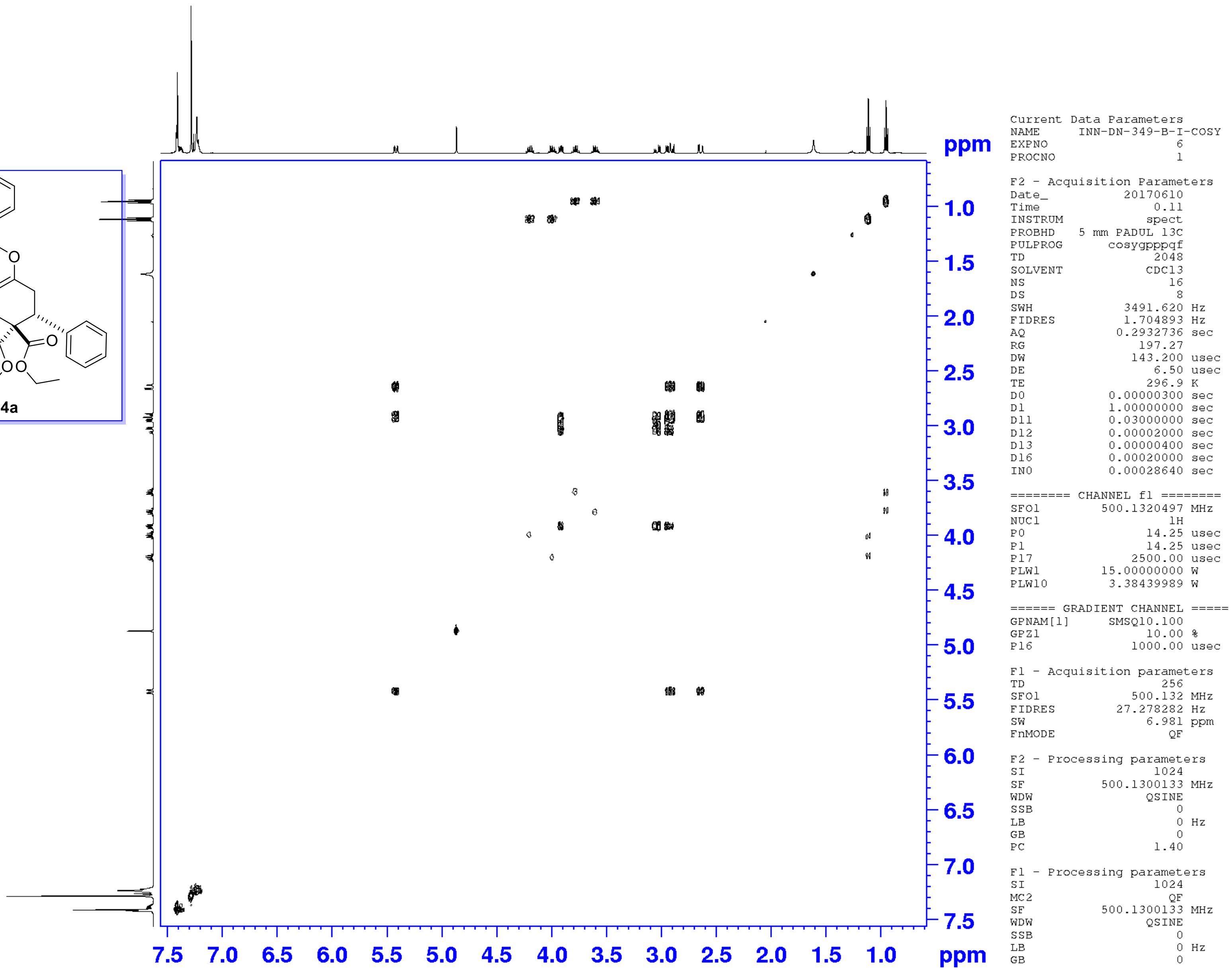

Fig S5. ^{1}H -^{1}H COSY NMR Spectrum of 4a

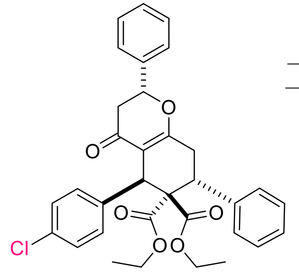

4a

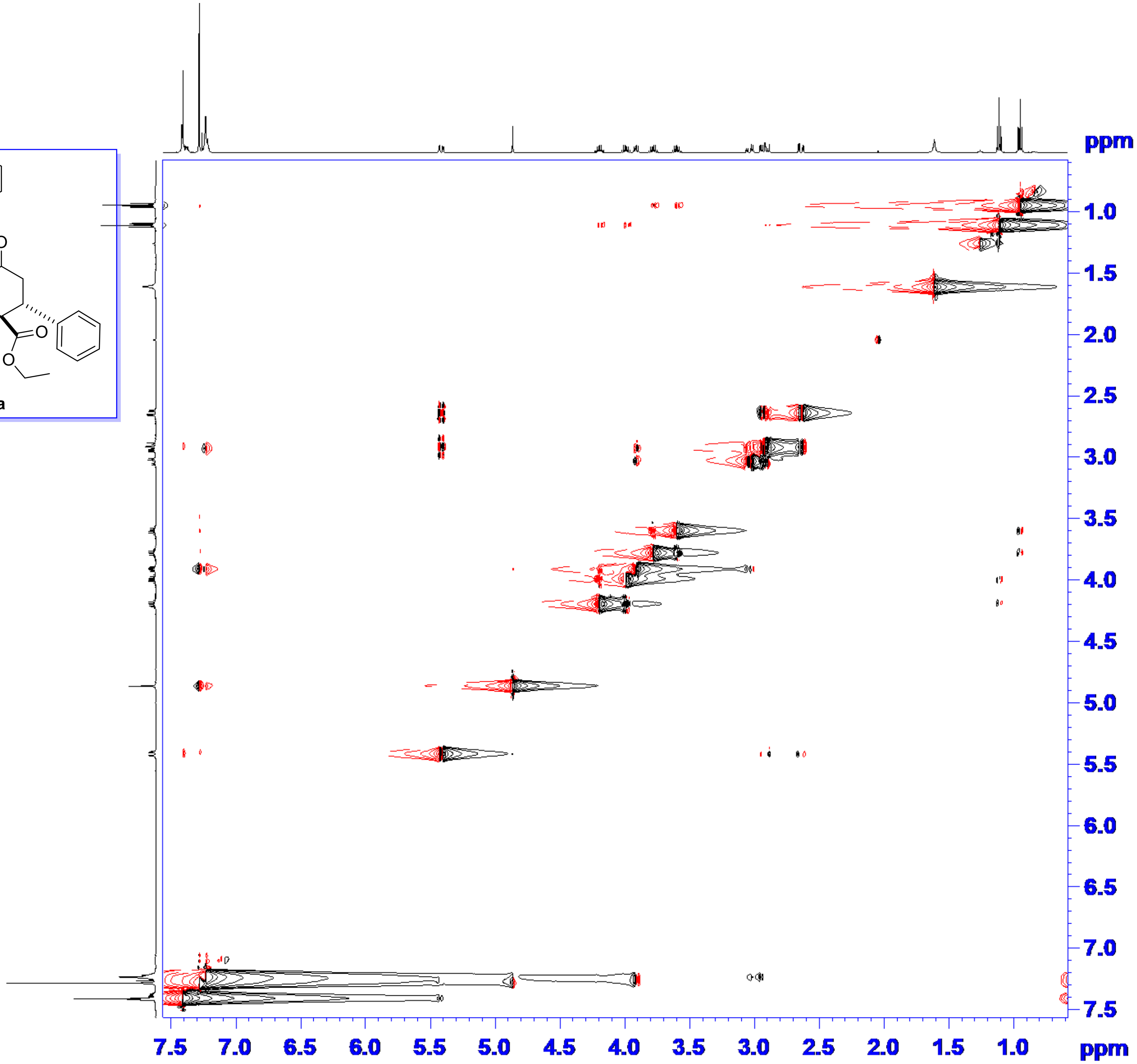

Current Data Parameters
NAME INN-DN-349-B-I-NOESY
EXPNO 6
PROCNO 1

F2 - Acquisition Parameters
Date_ 20170610
Time 1.47
INSTRUM spect
PROBHD 5 mm PADUL 13C
PULPROG noesygpph
TD 2048
SOLVENT CDCl3
NS 32
DS 32
SWH 3491.620 Hz
FIDRES 1.704893 Hz
AQ 0.2932736 sec
RG 197.27
DW 143.200 usec
DE 6.50 usec
TE 296.6 K
D0 0.00012506 sec
D1 1.00000000 sec
D8 0.60000002 sec
D16 0.00020000 sec
IN0 0.00028640 sec

======== CHANNEL f1 ========
SFO1 500.1320497 MHz
NUC1 1H
P1 14.25 usec
P2 28.50 usec
PLW1 15.00000000 W

====== GRADIENT CHANNEL =====
GPNAM[1] SMSQ10.100
GPZ1 40.00 %
P16 1000.00 usec

F1 - Acquisition parameters
TD 256
SFO1 500.132 MHz
FIDRES 27.278282 Hz
SW 6.981 ppm
FnMODE TPPI

F2 - Processing parameters
SI 1024
SF 500.1300133 MHz
WDW QSINE
SSB 2
LB 0 Hz
GB 0
PC 1.00

F1 - Processing parameters
SI 1024
MC2 TPPI
SF 500.1300133 MHz
WDW QSINE
SSB 2
LB 0 Hz
GB 0

Fig S6. ^{1}H -^{1}H NOESY NMR Spectrum of 4a

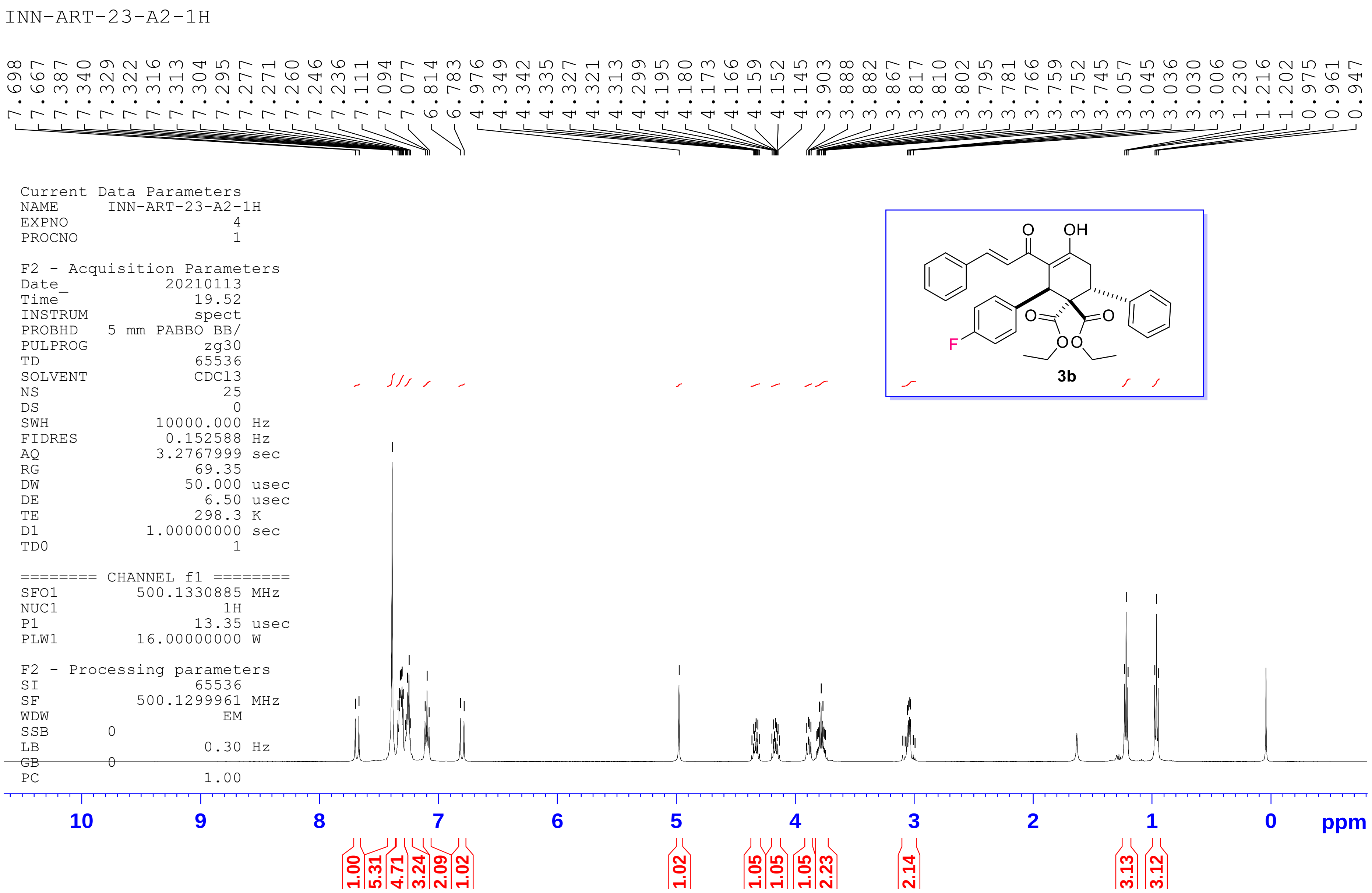

Fig S7. ^{1}**H NMR Spectrum of 3b**

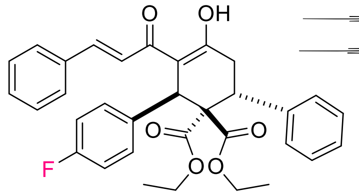

3b

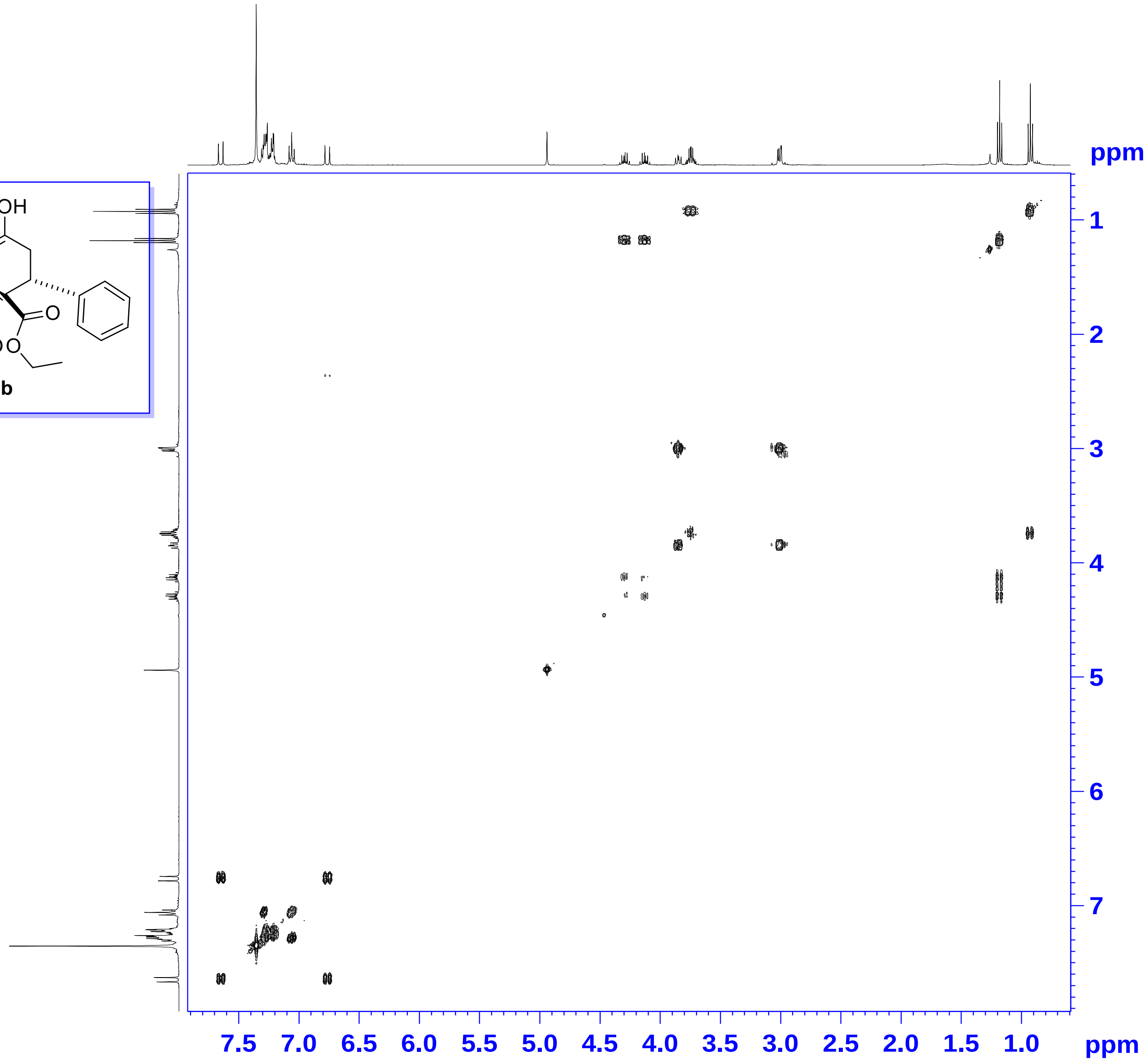


```
Current Data Parameters
NAME      INN-DN-Ph-Ph-F-COSY
EXPNO                   4
PROCNO                  1

F2 - Acquisition Parameters
Date_            20170327
Time                17.50
INSTRUM             spect
PROBHD    5 mm PABBO BB-
PULPROG          cosygpqf
TD                   2048
SOLVENT             CDCl3
NS                     16
DS                     16
SWH              2934.272 Hz
FIDRES           1.432750 Hz
AQ              0.3489792 sec
RG                    256
DW                170.400 usec
DE                   6.50 usec
TE                  298.2 K
D0             0.00000300 sec
D1             1.00000000 sec
D13            0.00000400 sec
D16            0.00020000 sec
IN0            0.00034080 sec

======== CHANNEL f1 ========
NUC1                   1H
P0                  14.75 usec
P1                  14.75 usec
PL1                 -1.00 dB
PL1W          10.56200695 W
SFO1          400.1317136 MHz

====== GRADIENT CHANNEL =====
GPNAM[1]         SINE.100
GPZ1                10.00 %
P16               1000.00 usec

F1 - Acquisition parameters
TD                    256
SFO1             400.1317 MHz
FIDRES          22.924109 Hz
SW                  7.333 ppm
FnMODE                 QF

F2 - Processing parameters
SI                   2048
SF            400.1300100 MHz
WDW                  SINE
SSB       0
LB        0 Hz
GB        0
PC                   1.40

F1 - Processing parameters
SI                    512
MC2                    QF
SF            400.1300100 MHz
WDW                  SINE
SSB       0
LB        0 Hz
GB        0
```

Fig S8. ^{1}H -^{1}H **COSY NMR Spectrum of 3b**

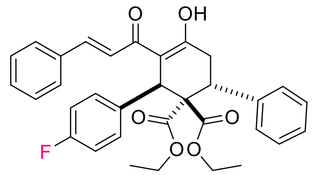

3b

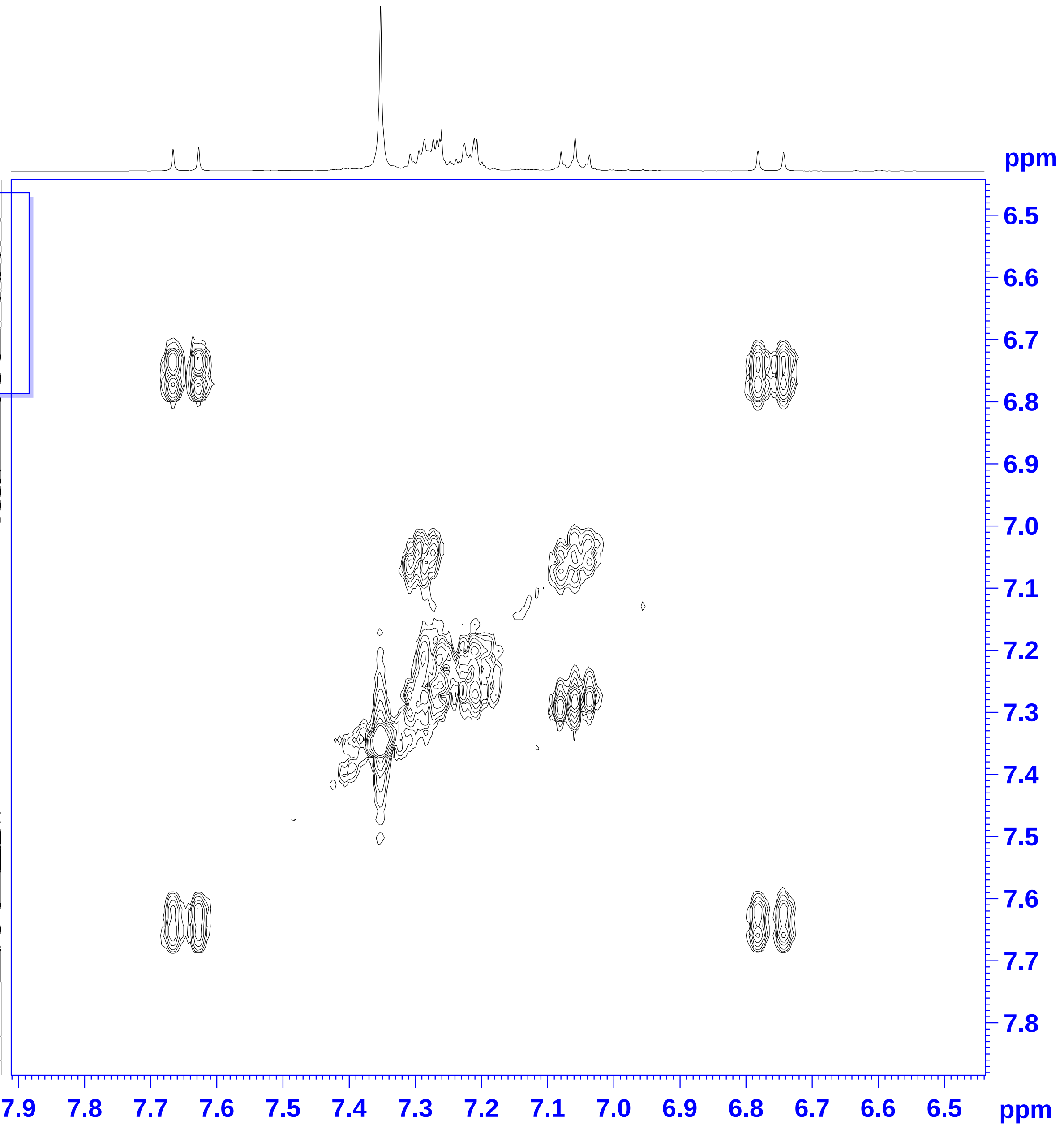


```
Current Data Parameters
NAME      INN-DN-Ph-Ph-F-COSY
EXPNO                   4
PROCNO                  1

F2 - Acquisition Parameters
Date_            20170327
Time                17.50
INSTRUM             spect
PROBHD   5 mm PABBO BB-
PULPROG          cosygpqf
TD                   2048
SOLVENT             CDCl3
NS                     16
DS                     16
SWH              2934.272 Hz
FIDRES           1.432750 Hz
AQ              0.3489792 sec
RG                    256
DW                170.400 usec
DE                   6.50 usec
TE                  298.2 K
D0             0.00000300 sec
D1             1.00000000 sec
D13            0.00000400 sec
D16            0.00020000 sec
IN0            0.00034080 sec

======== CHANNEL f1 ========
NUC1                   1H
P0                  14.75 usec
P1                  14.75 usec
PL1                 -1.00 dB
PL1W          10.56200695 W
SFO1          400.1317136 MHz

====== GRADIENT CHANNEL =====
GPNAM[1]         SINE.100
GPZ1                10.00 %
P16               1000.00 usec

F1 - Acquisition parameters
TD                    256
SFO1             400.1317 MHz
FIDRES          22.924109 Hz
SW                  7.333 ppm
FnMODE                 QF

F2 - Processing parameters
SI                   2048
SF            400.1300100 MHz
WDW                  SINE
SSB      0
LB       0 Hz
GB       0
PC                   1.40

F1 - Processing parameters
SI                    512
MC2                    QF
SF            400.1300100 MHz
WDW                  SINE
SSB      0
LB       0 Hz
GB       0
```

Fig S9. ^{1}H -^{1}H COSY NMR Spectrum (expansion) of 3b

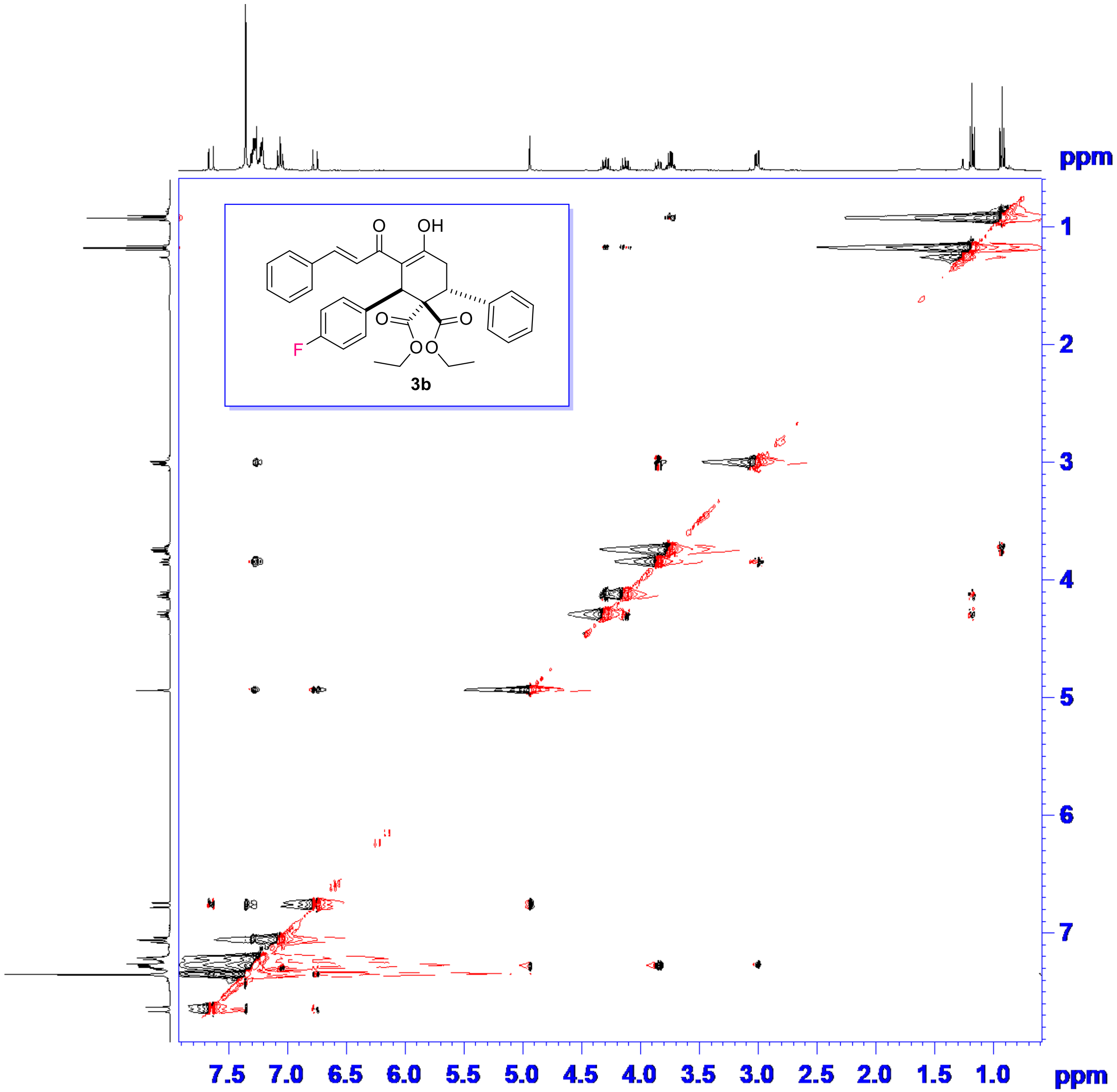

Current Data Parameters
NAME INN-DN-Ph-Ph-F-NOESY
EXPNO 5
PROCNO 1

F2 - Acquisition Parameters
Date_ 20170327
Time_ 19.26
INSTRUM spect
PROBHD 5 mm PABBO BB-
PULPROG noesyph
TD 2048
SOLVENT CDCl3
NS 16
DS 16
SWH 2934.272 Hz
FIDRES 1.432750 Hz
AQ 0.3489792 sec
RG 57
DW 170.400 usec
DE 6.50 usec
TE 297.4 K
D0 0.00015162 sec
D1 1.00000000 sec
D8 0.60000002 sec
IN0 0.00034080 sec

======= CHANNEL f1 =======
NUC1 1H
P1 14.75 usec
PL1 -1.00 dB
PL1W 10.56200695 W
SFO1 400.1317136 MHz

F1 - Acquisition parameters
TD 512
SFO1 400.1317 MHz
FIDRES 11.462054 Hz
SW 7.333 ppm
FnMODE States-TPPI

F2 - Processing parameters
SI 2048
SF 400.1300100 MHz
WDW QSINE
SSB 2
LB 0 Hz
GB 0
PC 1.00

F1 - Processing parameters
SI 512
MC2 States-TPPI
SF 400.1300100 MHz
WDW QSINE
SSB 2
LB 0 Hz
GB 0

Fig S10. ^{1}H -^{1}H NOESY NMR Spectrum of 3b

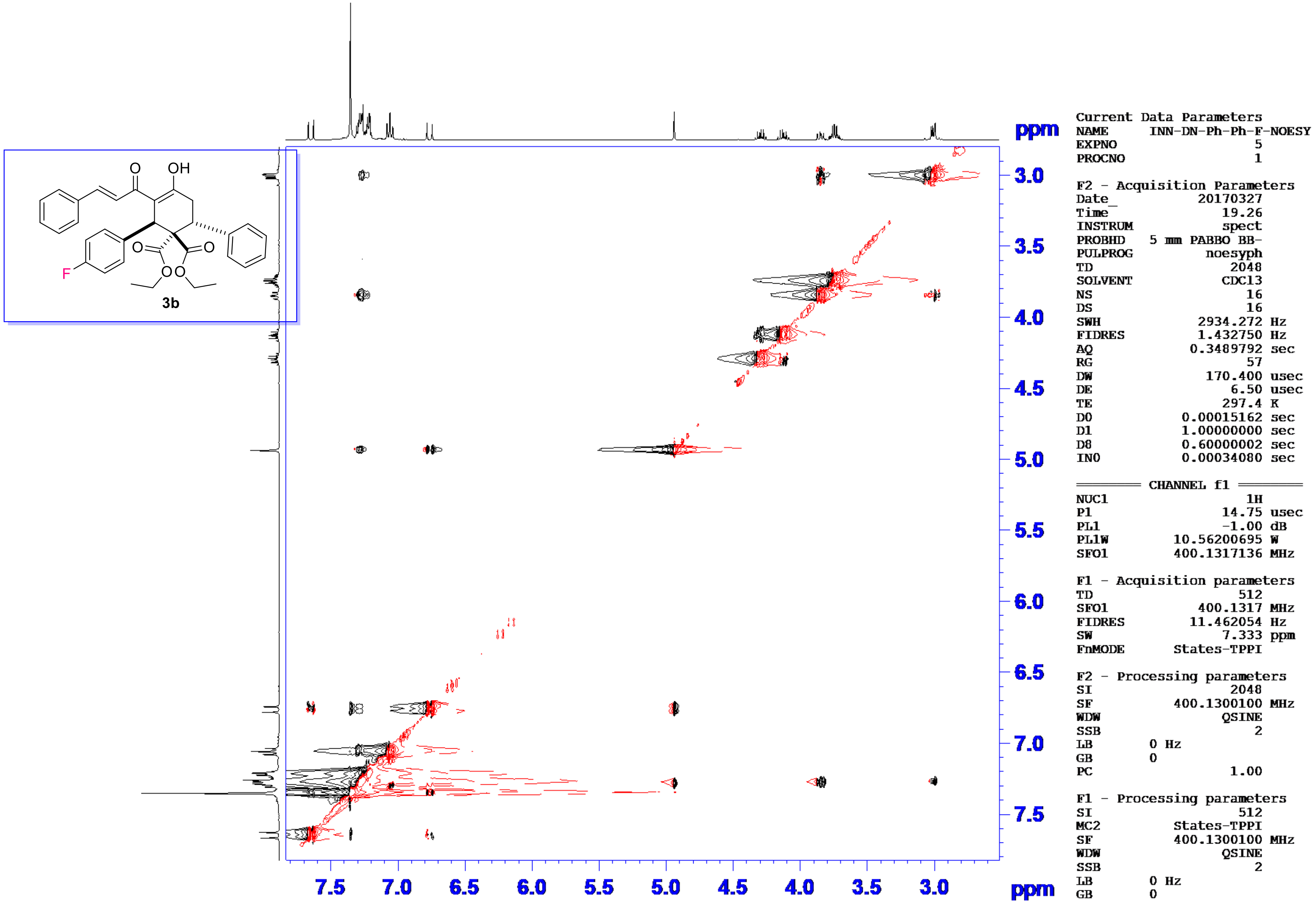

Fig S11. ^{1}H -^{1}H NOESY NMR Spectrum (expansion) of 3b

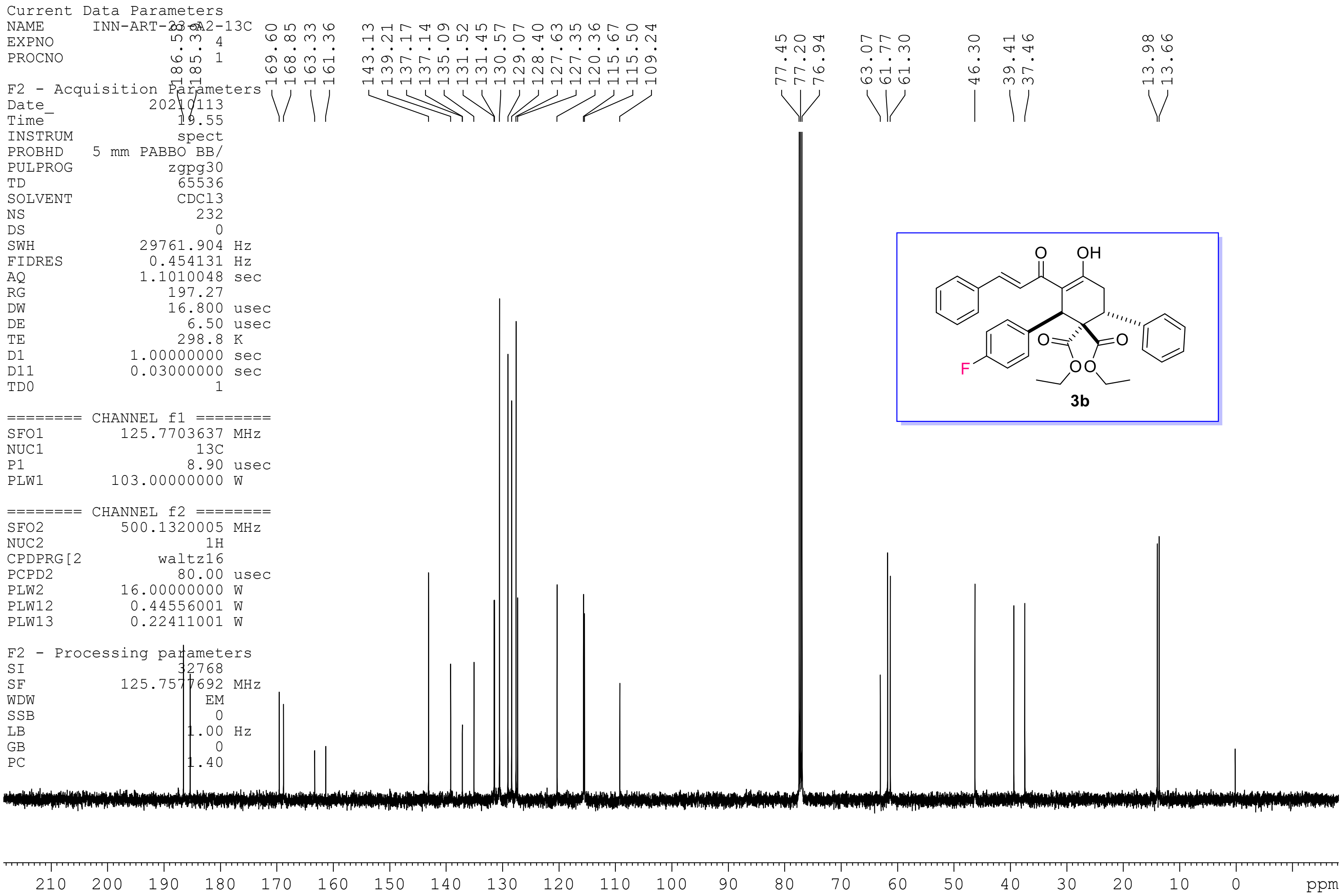

Fig S12. ^{13}**C NMR Spectrum of 3b**

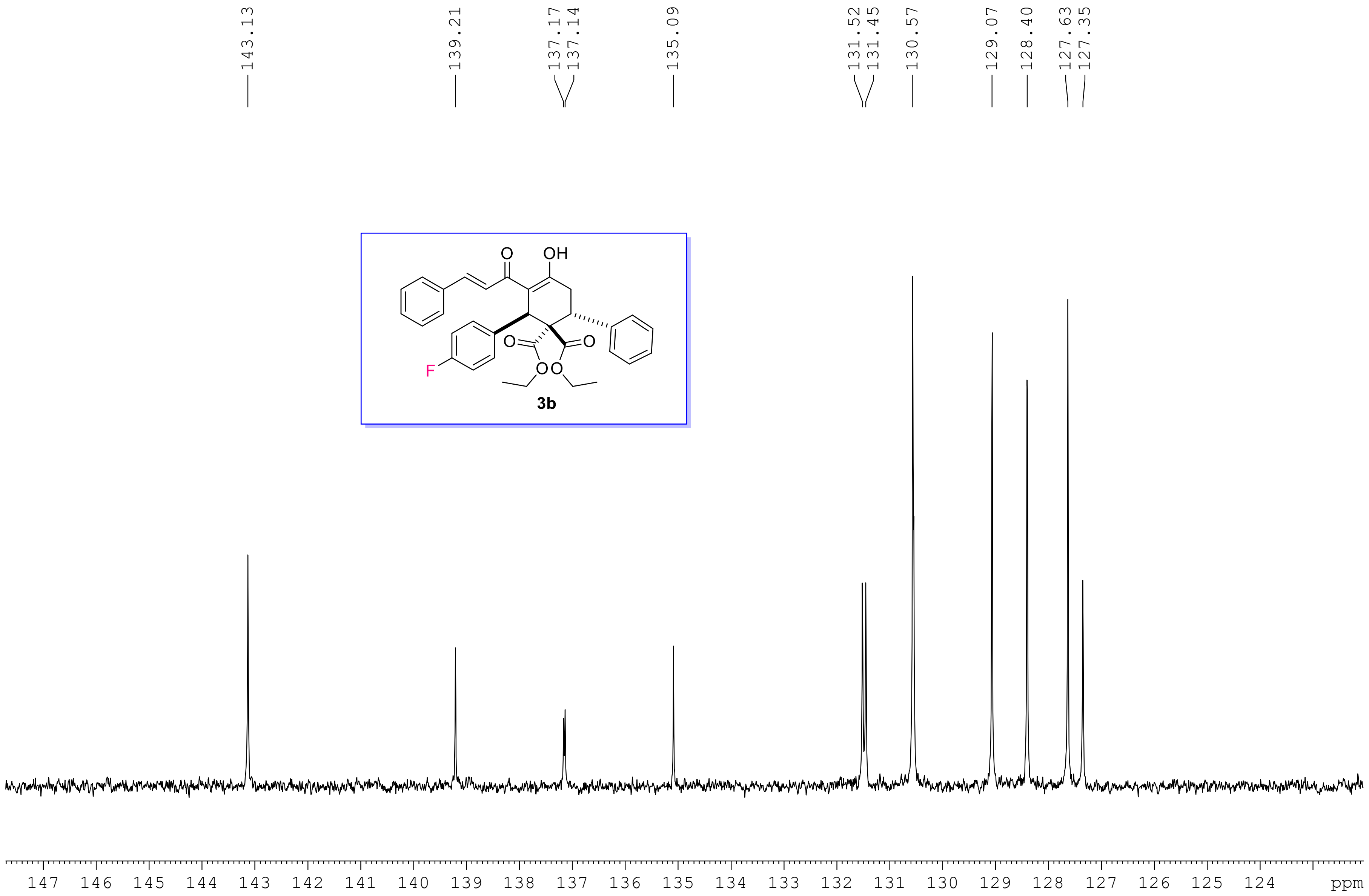

Fig S13. ^{13}C NMR Spectrum (expansion) of 3b

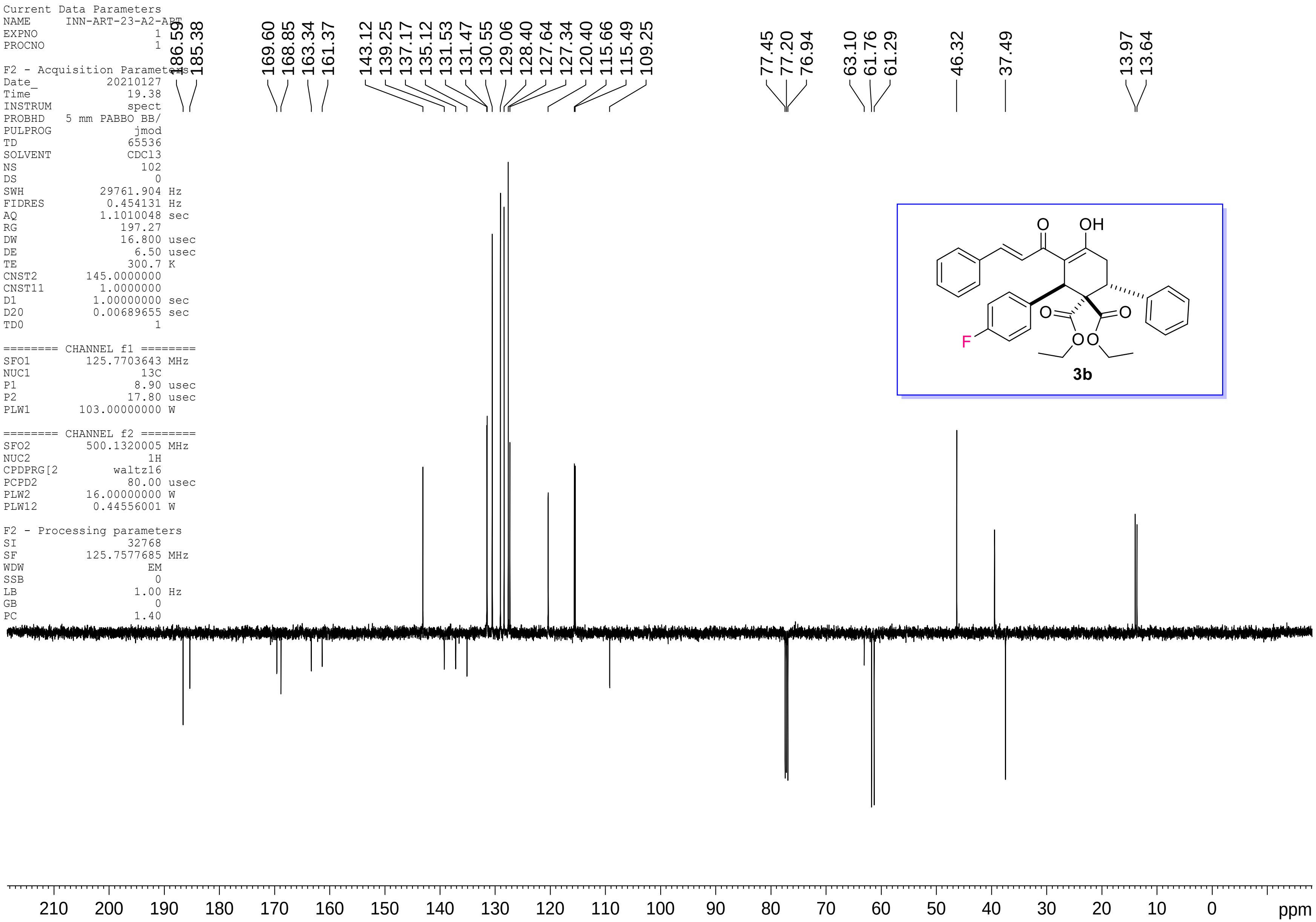

Fig S14. ^{13}C-APT Spectrum of 3b

INN-ART-23-A2--19F

```
Current Data Parameters
NAME      INN-ART-23-A2--19F
EXPNO                    3
PROCNO                   1

F2 - Acquisition Parameters
Date_           20210117
Time               11.56
INSTRUM            spect
PROBHD   5 mm PABBO BB/
PULPROG           zgflqn
TD                131072
SOLVENT            CDCl3
NS                   105
DS                     0
SWH           138888.891 Hz
FIDRES          1.059638 Hz
AQ             0.4718592 sec
RG                197.27
DW                 3.600 usec
DE                  6.50 usec
TE                 299.1 K
D1            1.00000000 sec
TD0                    1

======== CHANNEL f1 ========
SFO1         470.5453180 MHz
NUC1                 19F
P1                 19.75 usec
PLW1         55.00000000 W

F2 - Processing parameters
SI                 65536
SF           470.5923770 MHz
WDW                   EM
SSB      0
LB                  0.30 Hz
GB       0
PC                  1.00
```

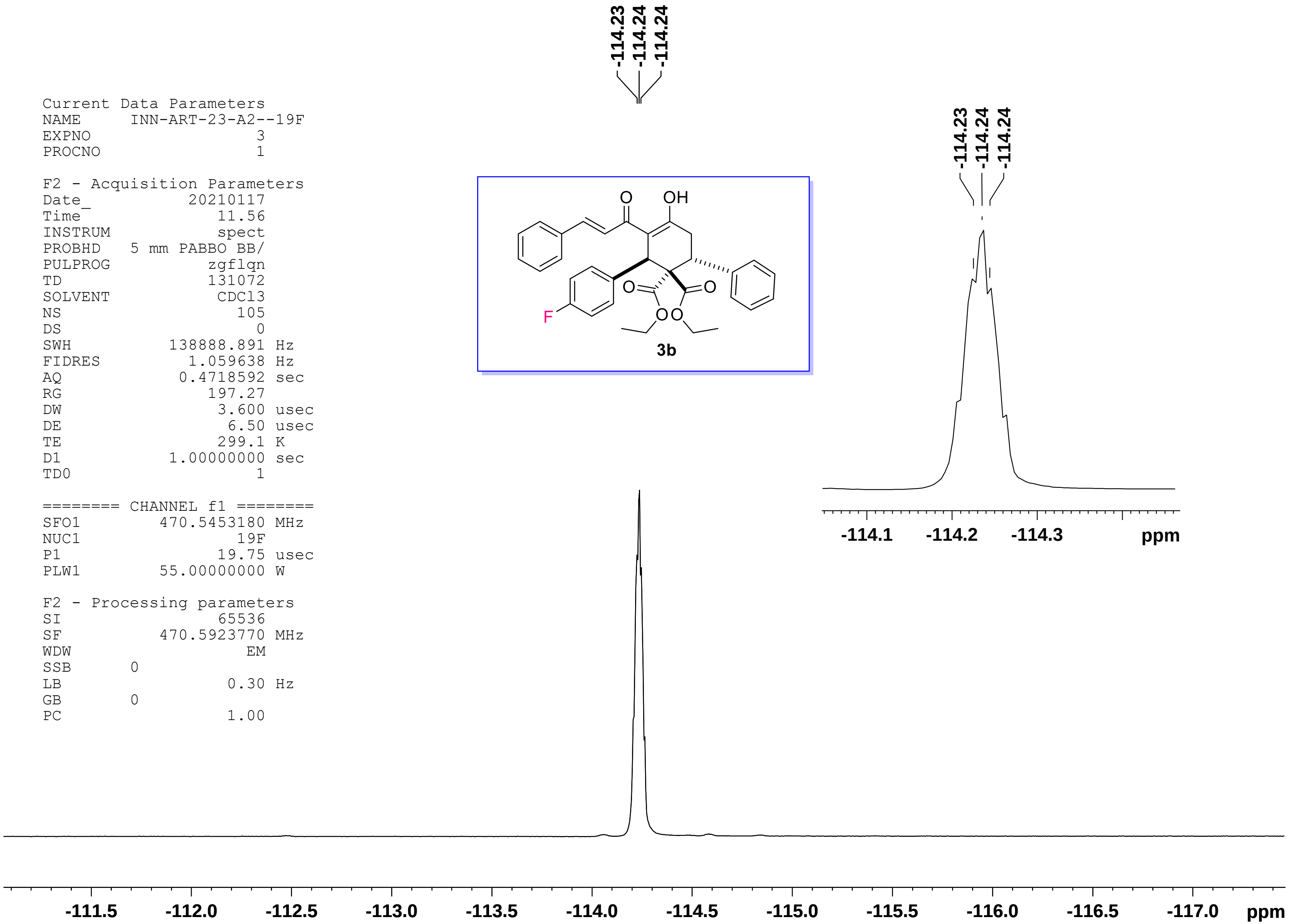

3b

Fig S15. ^{19}F NMR Spectrum of 3b

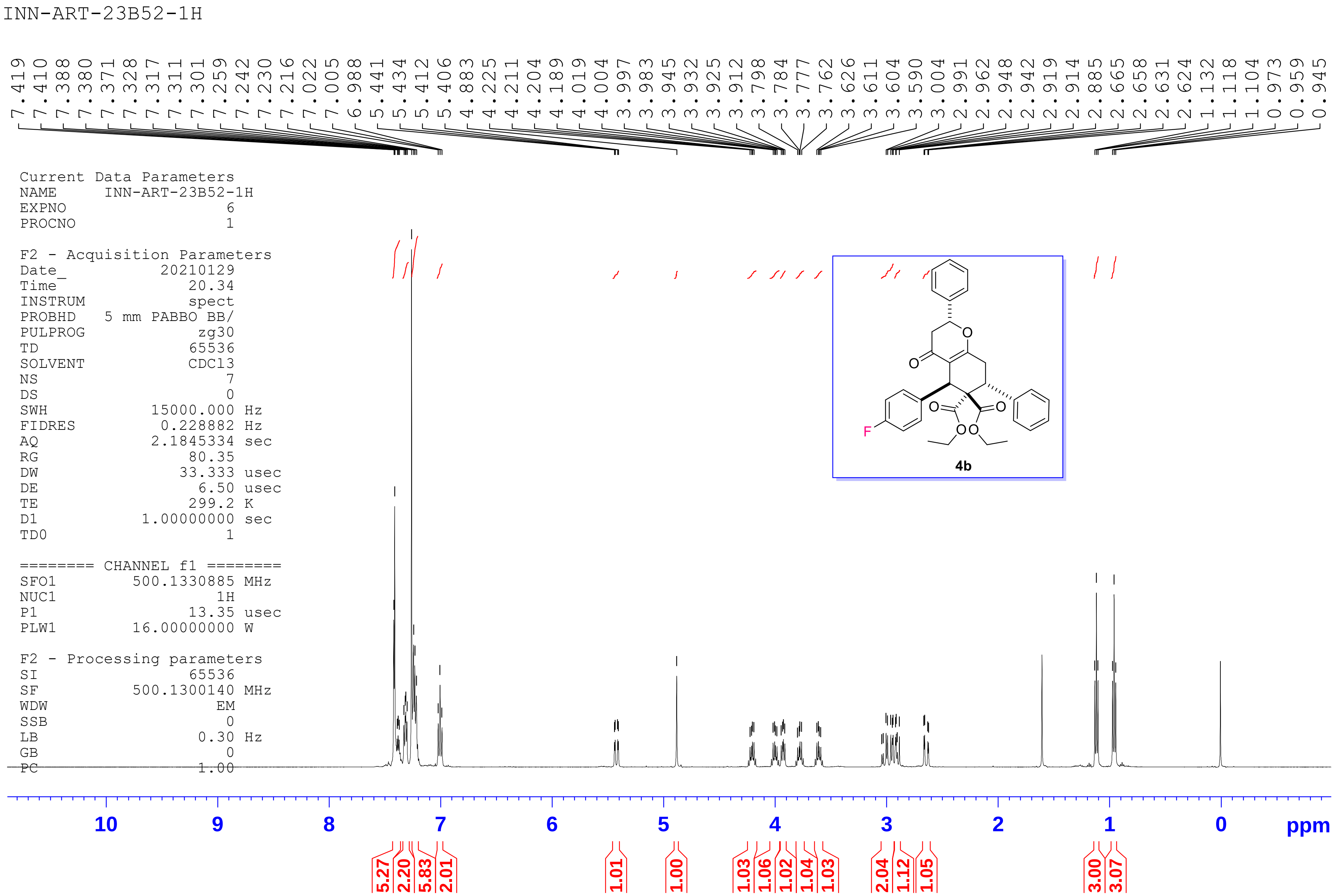

Fig S16. ¹H NMR spectrum of 4b

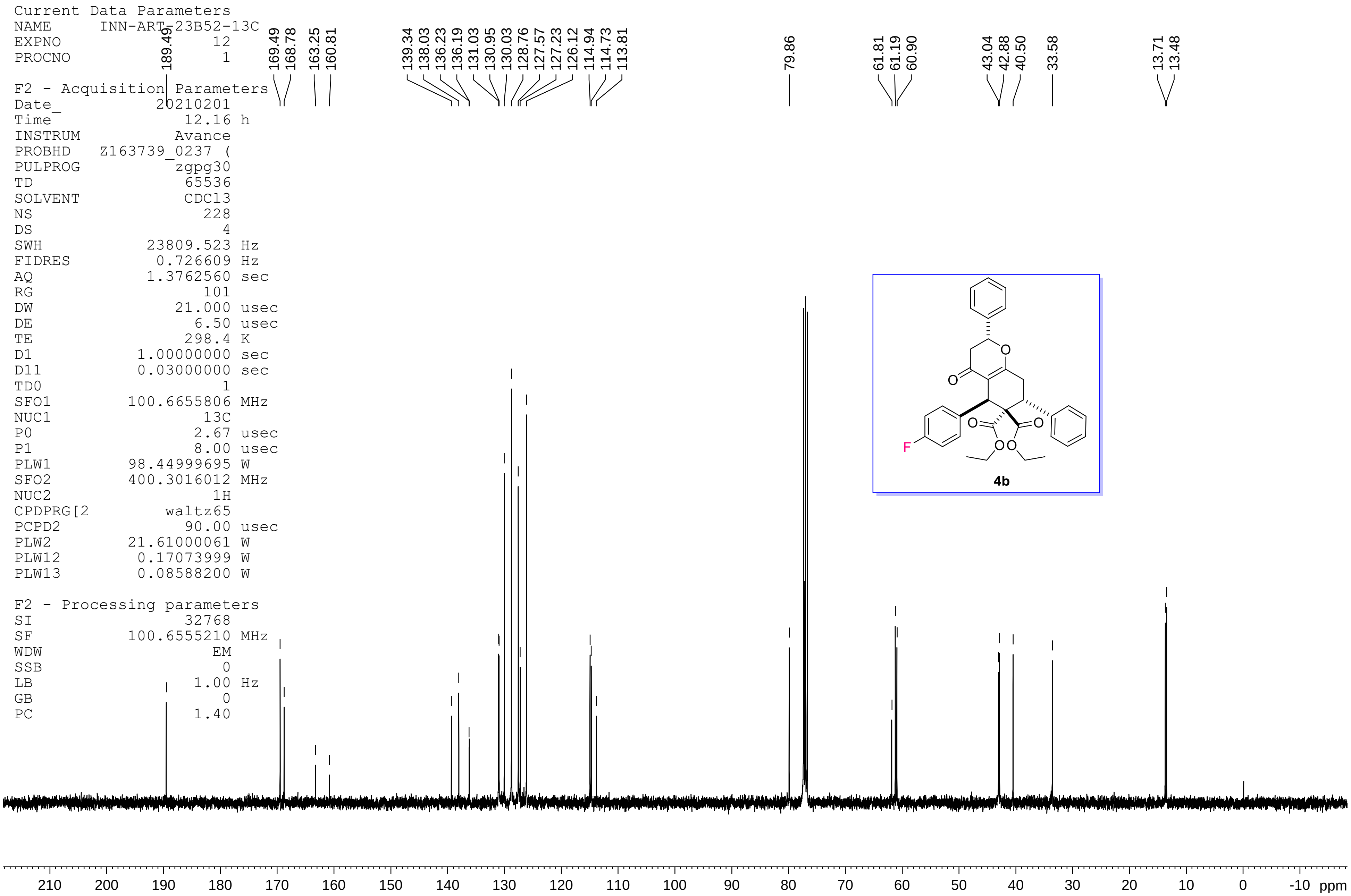

Fig S17. ^{13}**C NMR spectrum of 4b**

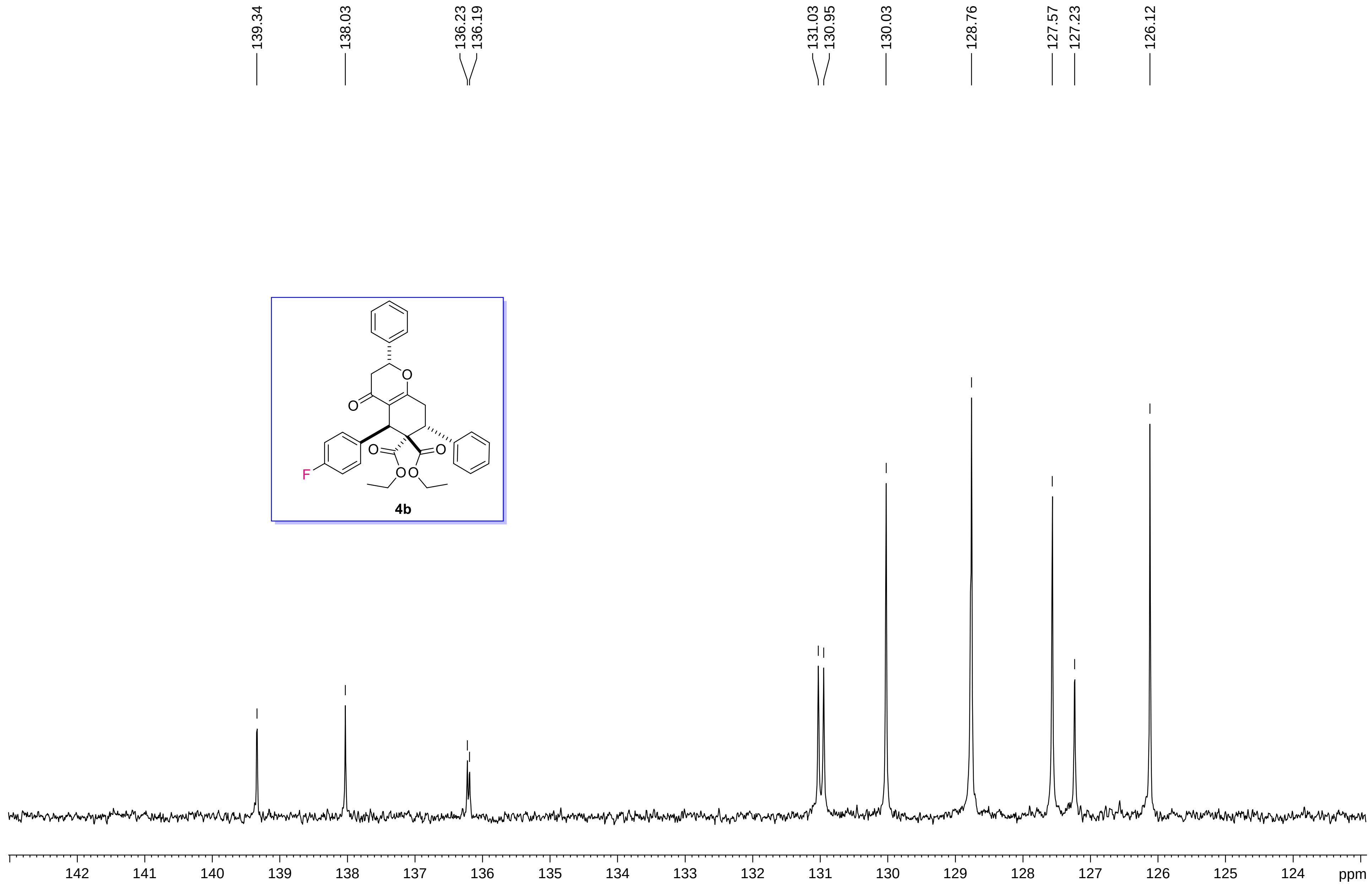

Fig S18. ^{13}C NMR spectrum (expansion) of 4b

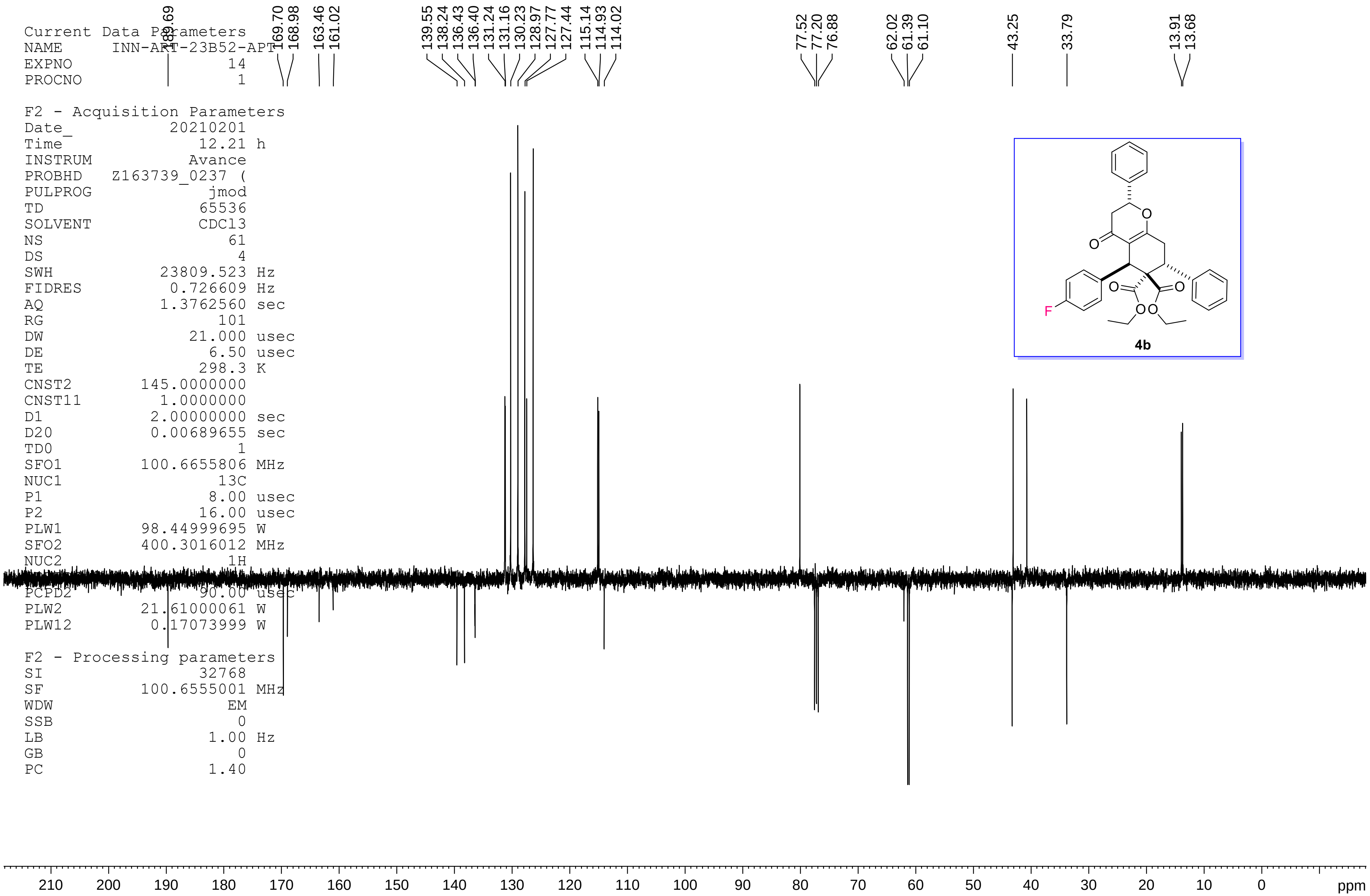

Fig S19. ^{13}C-APT spectrum of 4b

```
Current Data Parameters
NAME     INN-ART-25B-52-19F
EXPNO                     8
PROCNO                    1

F2 - Acquisition Parameters
Date_            20210211
Time                18.06
INSTRUM             spect
PROBHD   5 mm PABBO BB/
PULPROG            zgflqn
TD                 131072
SOLVENT             CDCl3
NS                     16
DS                      0
SWH           113636.367 Hz
FIDRES          0.866977 Hz
AQ             0.5767168 sec
RG                 197.27
DW                  4.400 usec
DE                   6.50 usec
TE                  298.0 K
D1             1.00000000 sec
TD0                     1

======== CHANNEL f1 ========
SFO1         470.5453180 MHz
NUC1                 19F
P1                 19.75 usec
PLW1         55.00000000 W

F2 - Processing parameters
SI                  65536
SF           470.5923770 MHz
WDW                    EM
SSB      0
LB                   0.30 Hz
GB       0
PC                   1.00
```

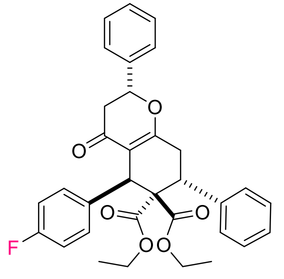

4b

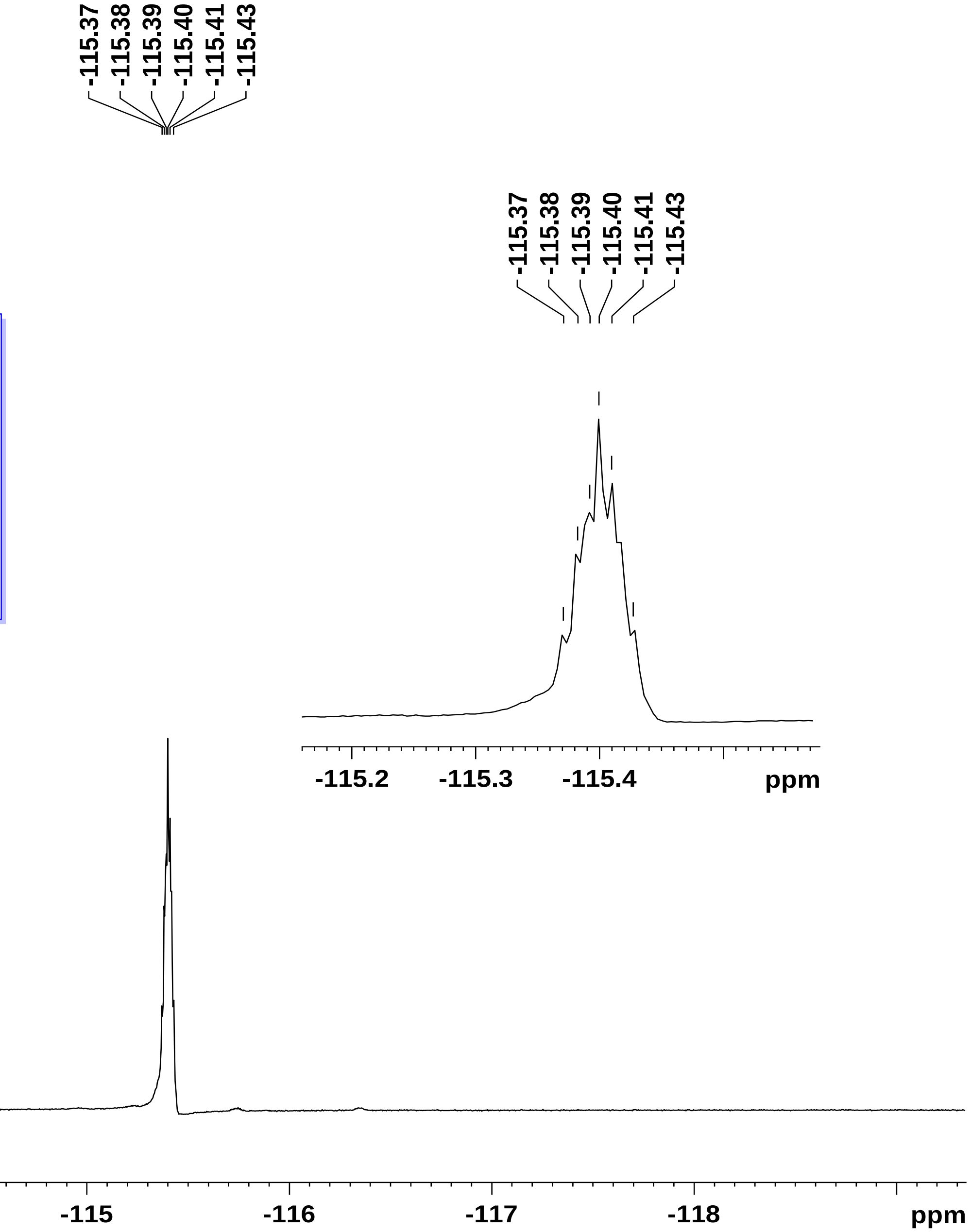

Fig S20. ^{19}F NMR spectrum of 4b

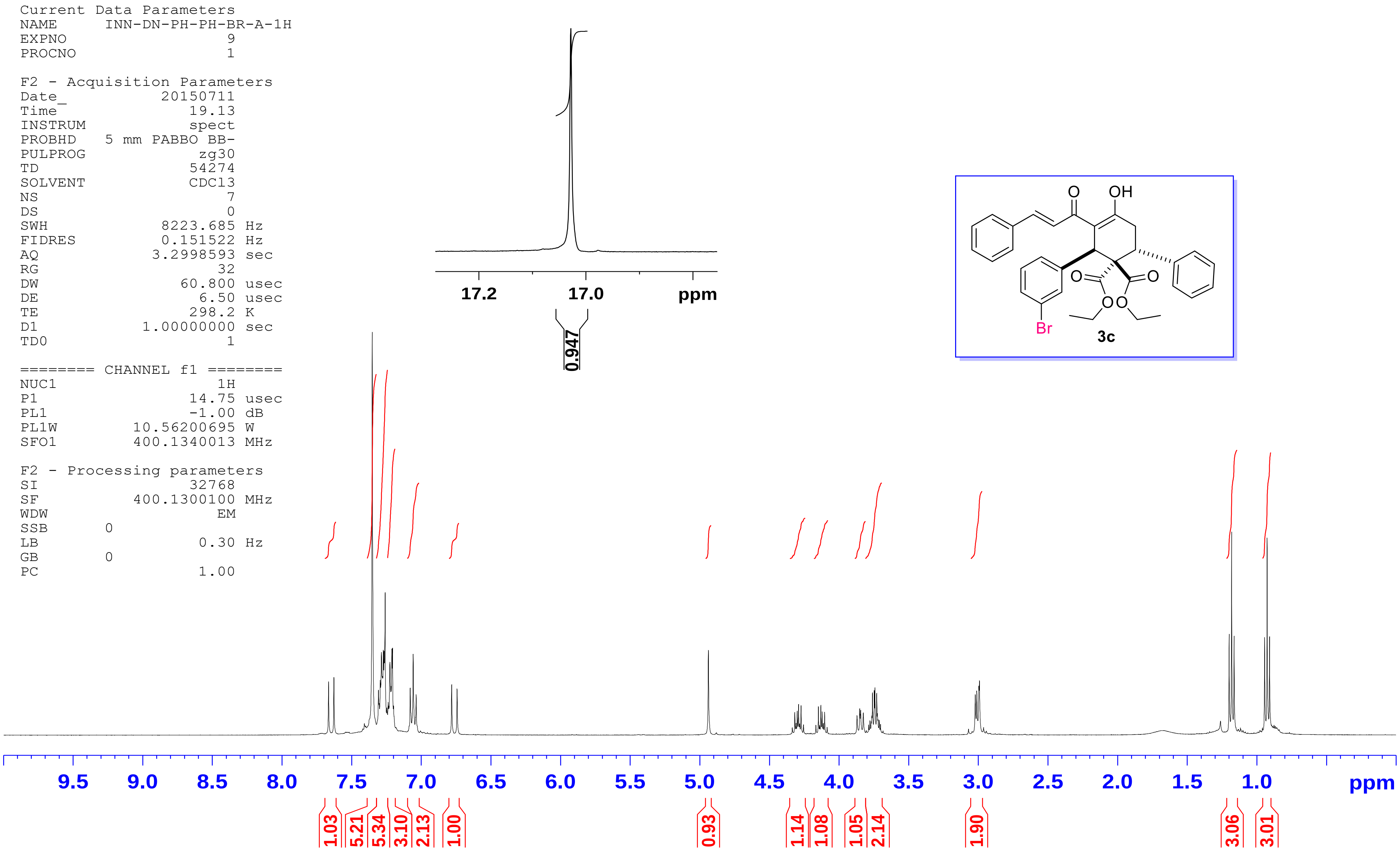

Fig S21. ^{1}H NMR Spectrum of 3c

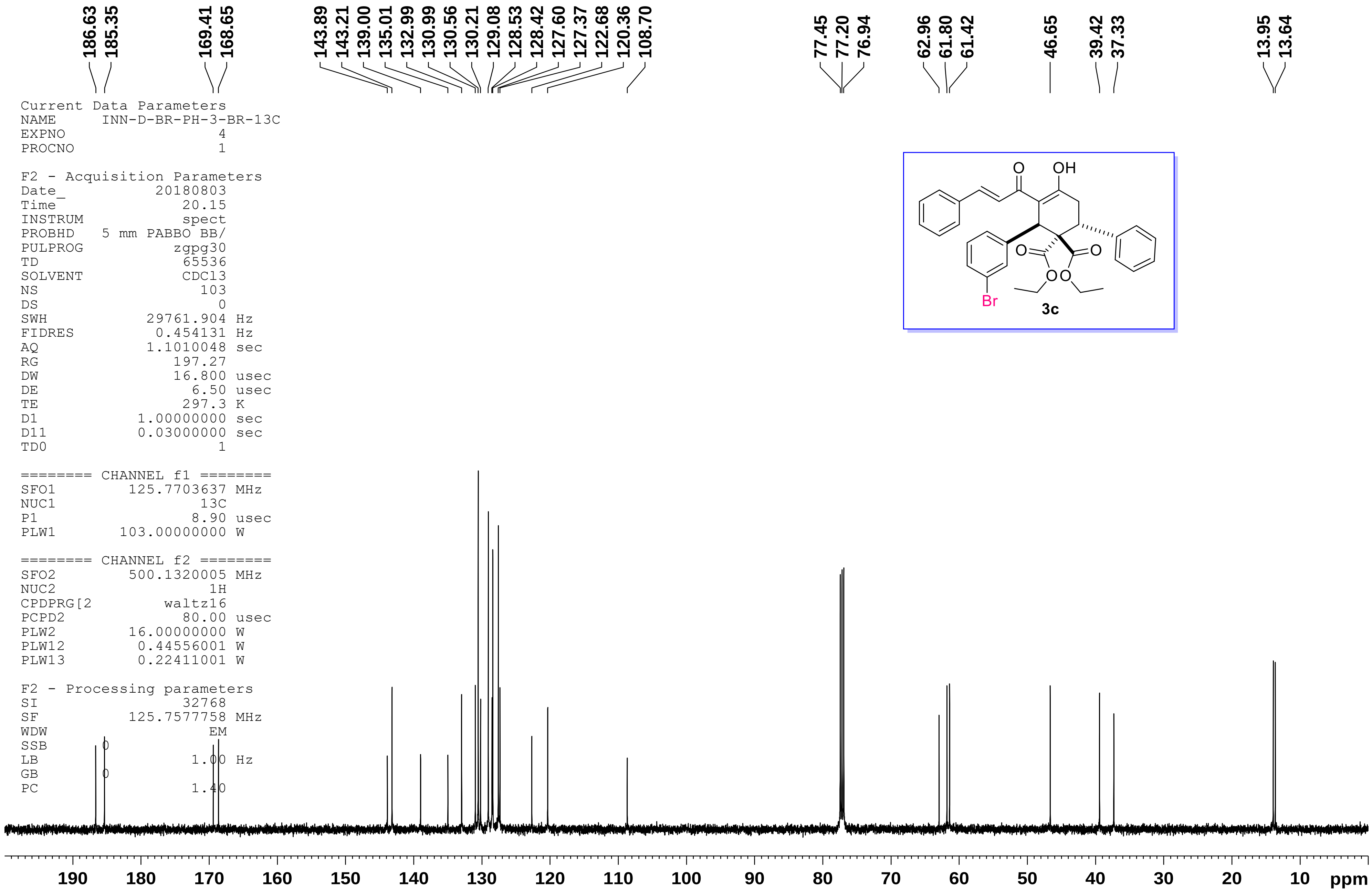

Fig S22. ^{13}C NMR spectrum of 3c

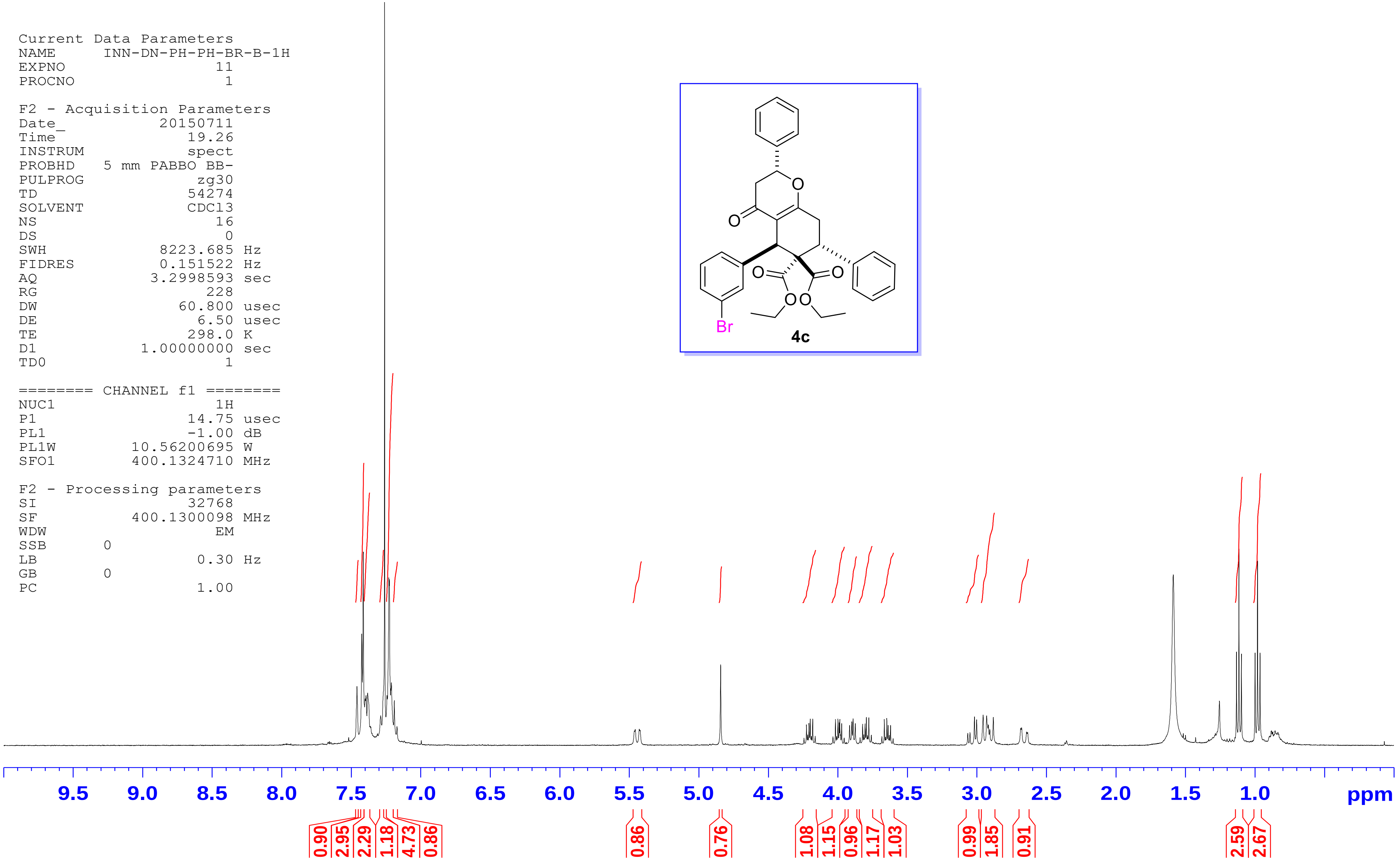

Fig S23. ^{1}H NMR Spectrum of 4c

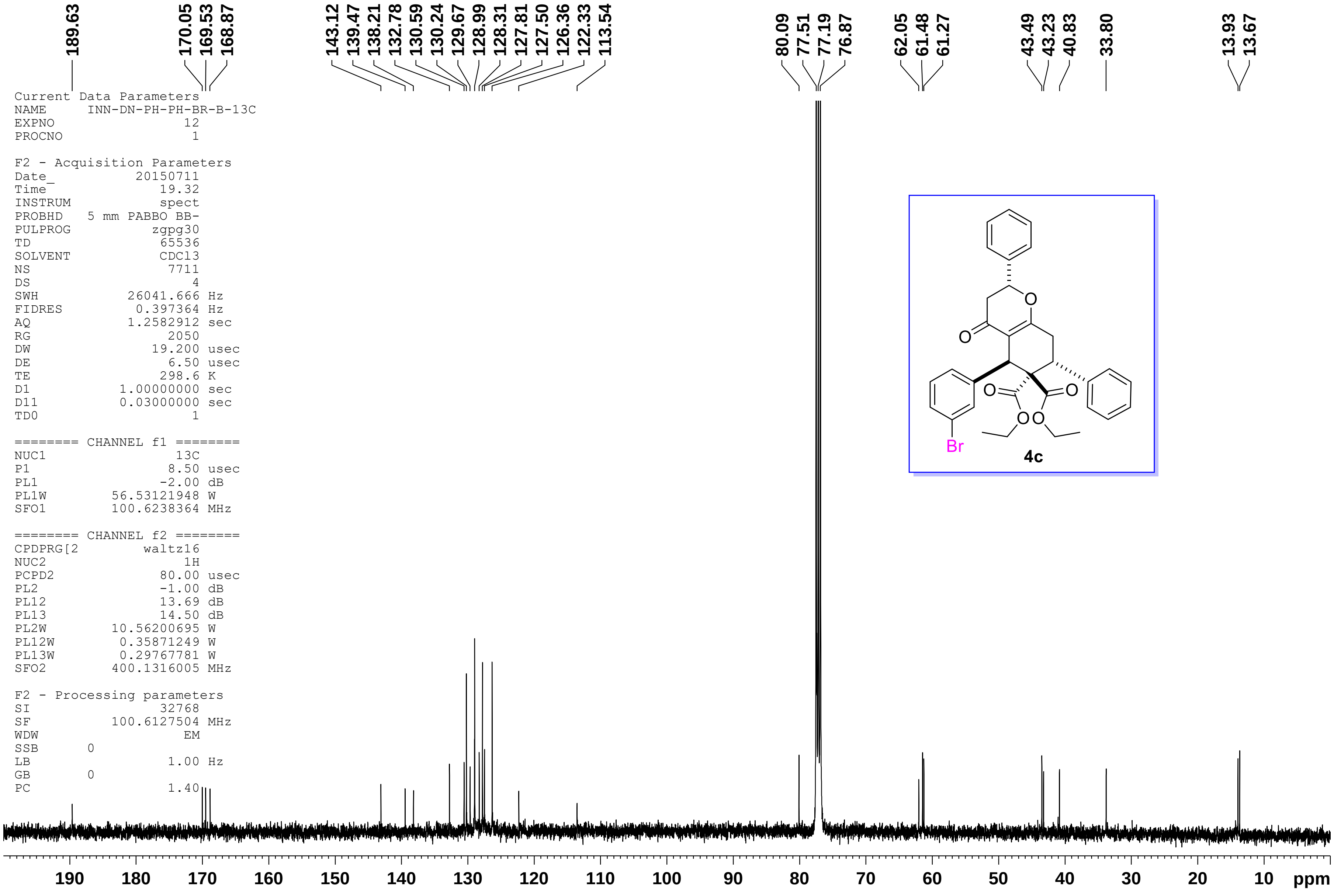

Fig S24. ^{13}C NMR Spectrum of 4c

```
Current Data Parameters
NAME       INN-DN-PH-PH-ME-1-1H
EXPNO                     3
PROCNO                    1

F2 - Acquisition Parameters
Date_              20170528
Time                  10.13
INSTRUM               spect
PROBHD   5 mm PABBO BB-
PULPROG                zg30
TD                    54274
SOLVENT               CDCl3
NS                       16
DS                        0
SWH                8223.685 Hz
FIDRES             0.151522 Hz
AQ                3.2998593 sec
RG                       32
DW                   60.800 usec
DE                     6.50 usec
TE                    299.6 K
D1               1.00000000 sec
TD0                       1

======== CHANNEL f1 ========
NUC1                     1H
P1                    14.75 usec
PL1                   -1.00 dB
PL1W            10.56200695 W
SFO1            400.1340013 MHz

F2 - Processing parameters
SI                    32768
SF              400.1300243 MHz
WDW                      EM
SSB      0
LB                     0.30 Hz
GB       0
PC                     1.00
```

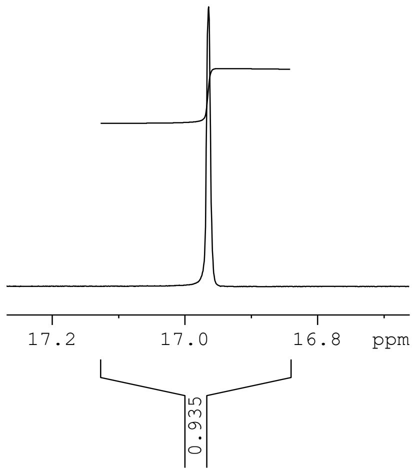

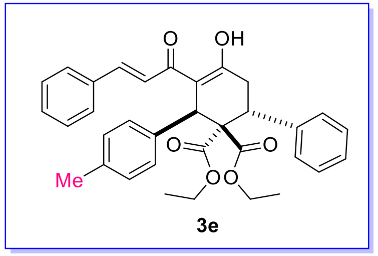

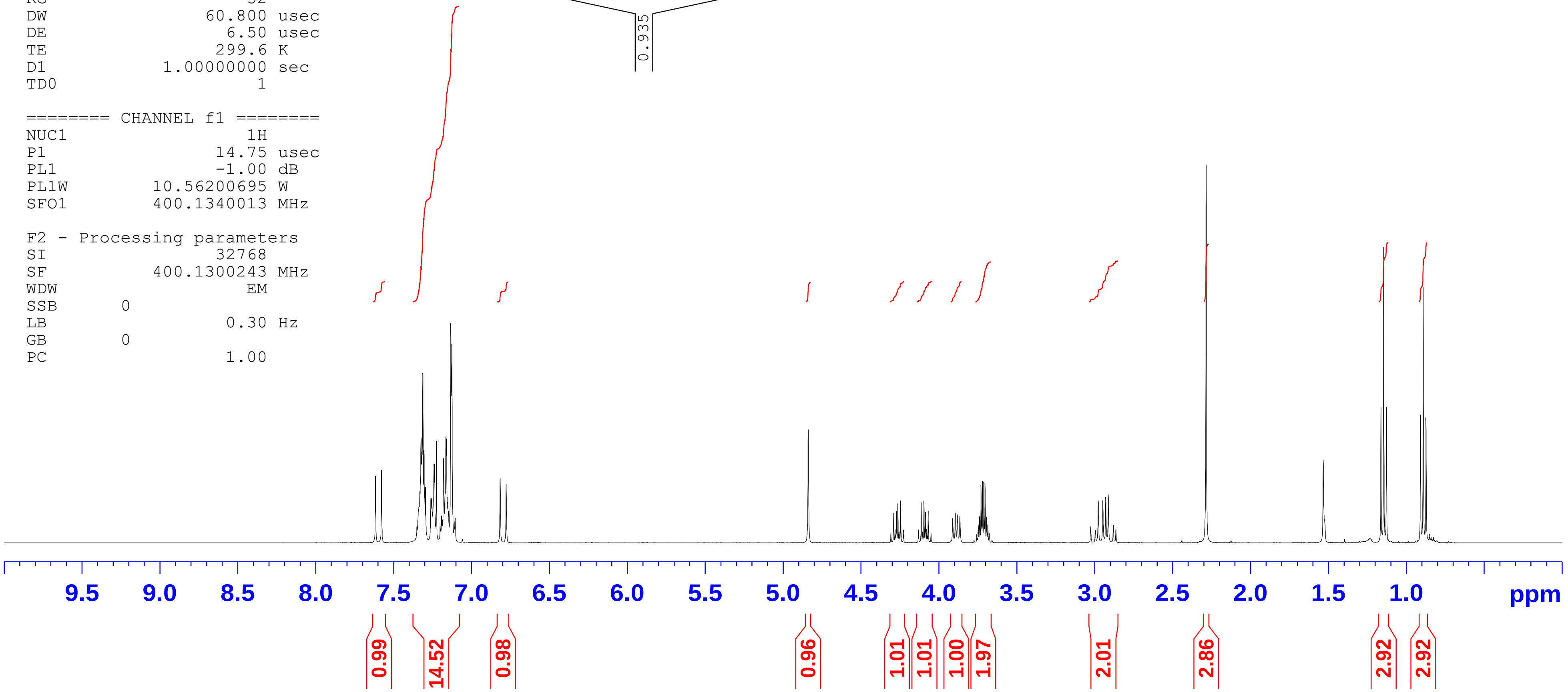

Fig S25. ^{1}H NMR Spectrum of 3e

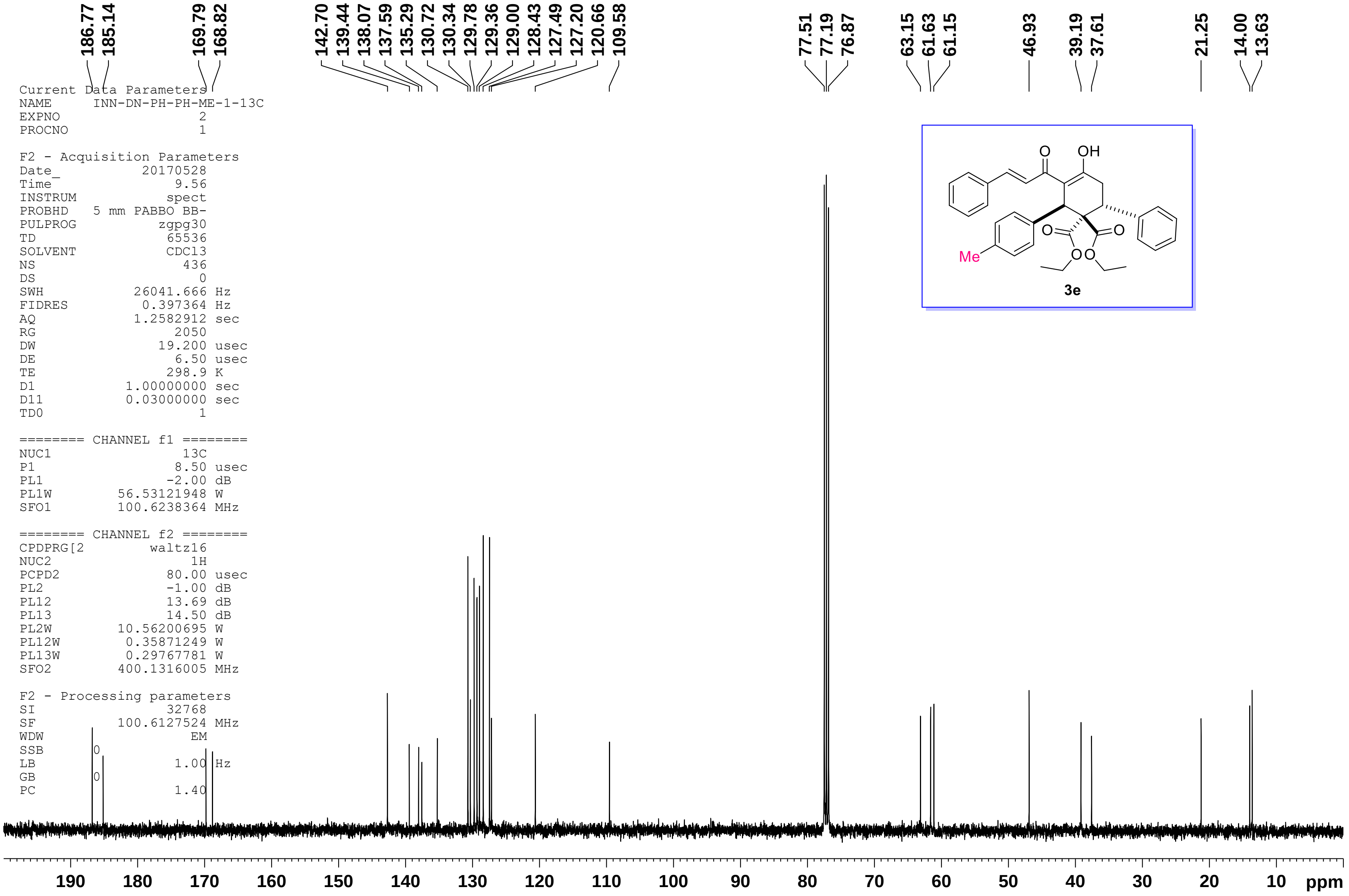

Fig S26. ^{13}C NMR Spectrum of 3e

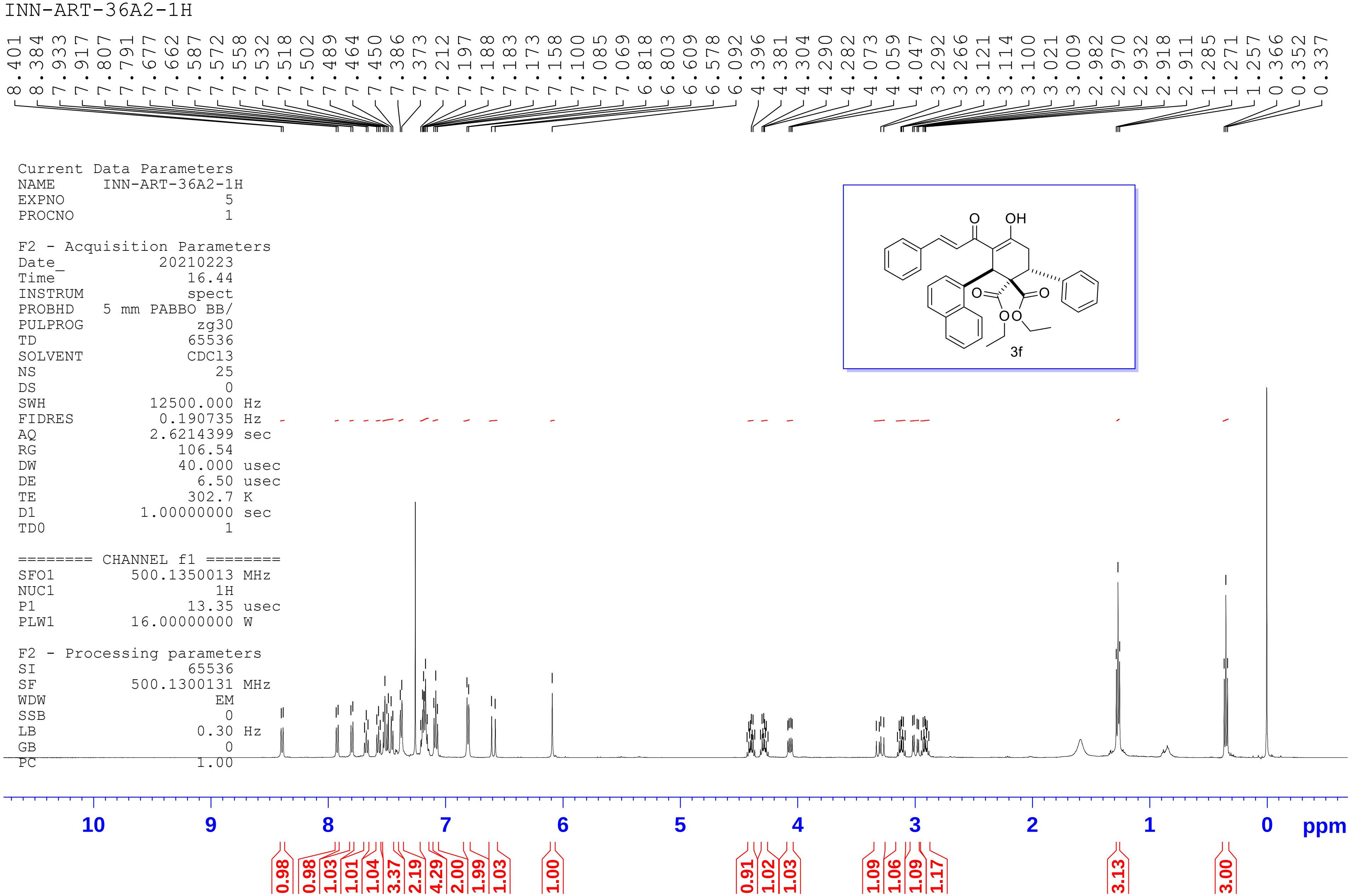

Fig S27. ¹H NMR spectrum of 3f

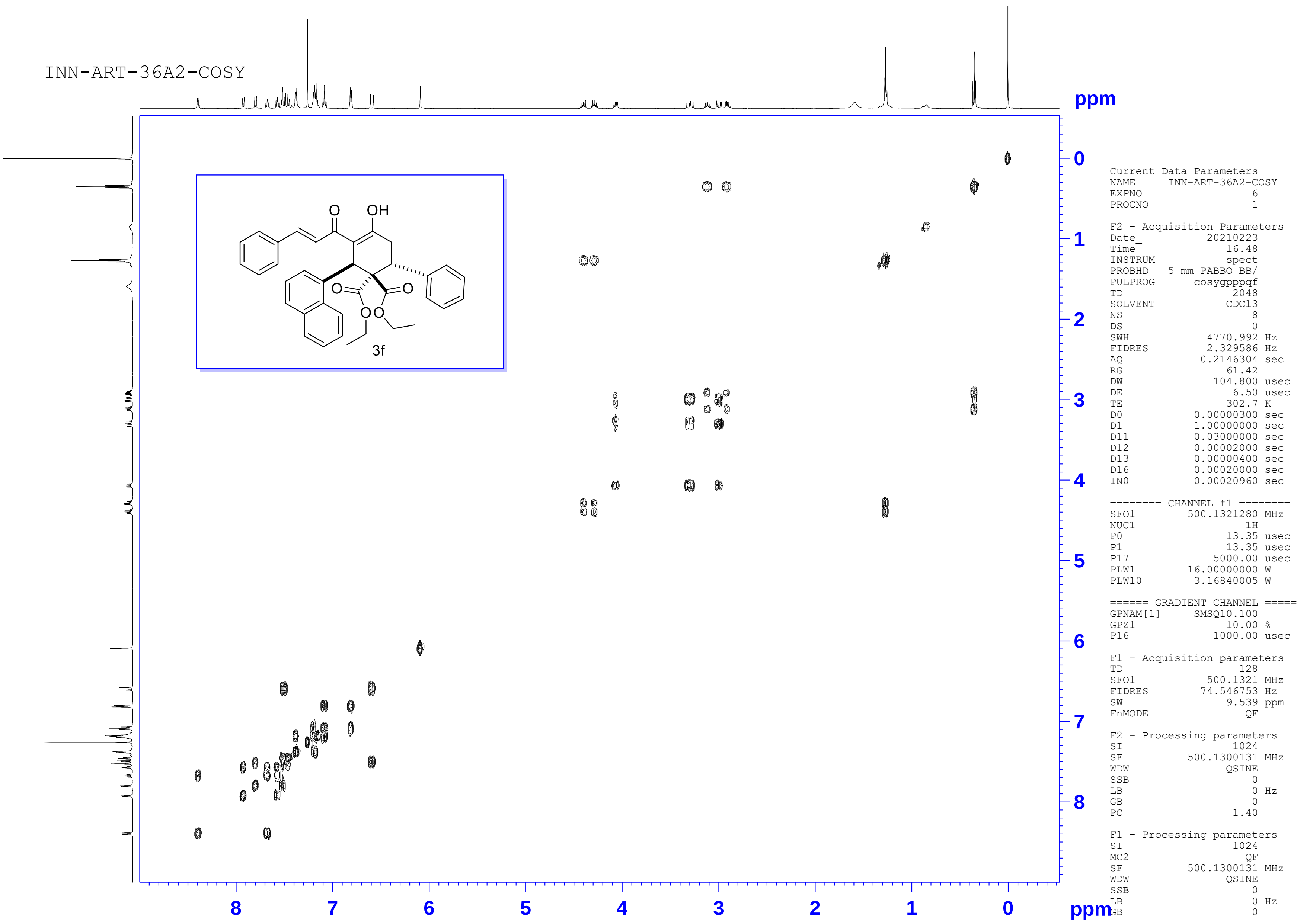

Fig S28. ^{1}H-^{1}H **COSY NMR Spectrum of 3f**

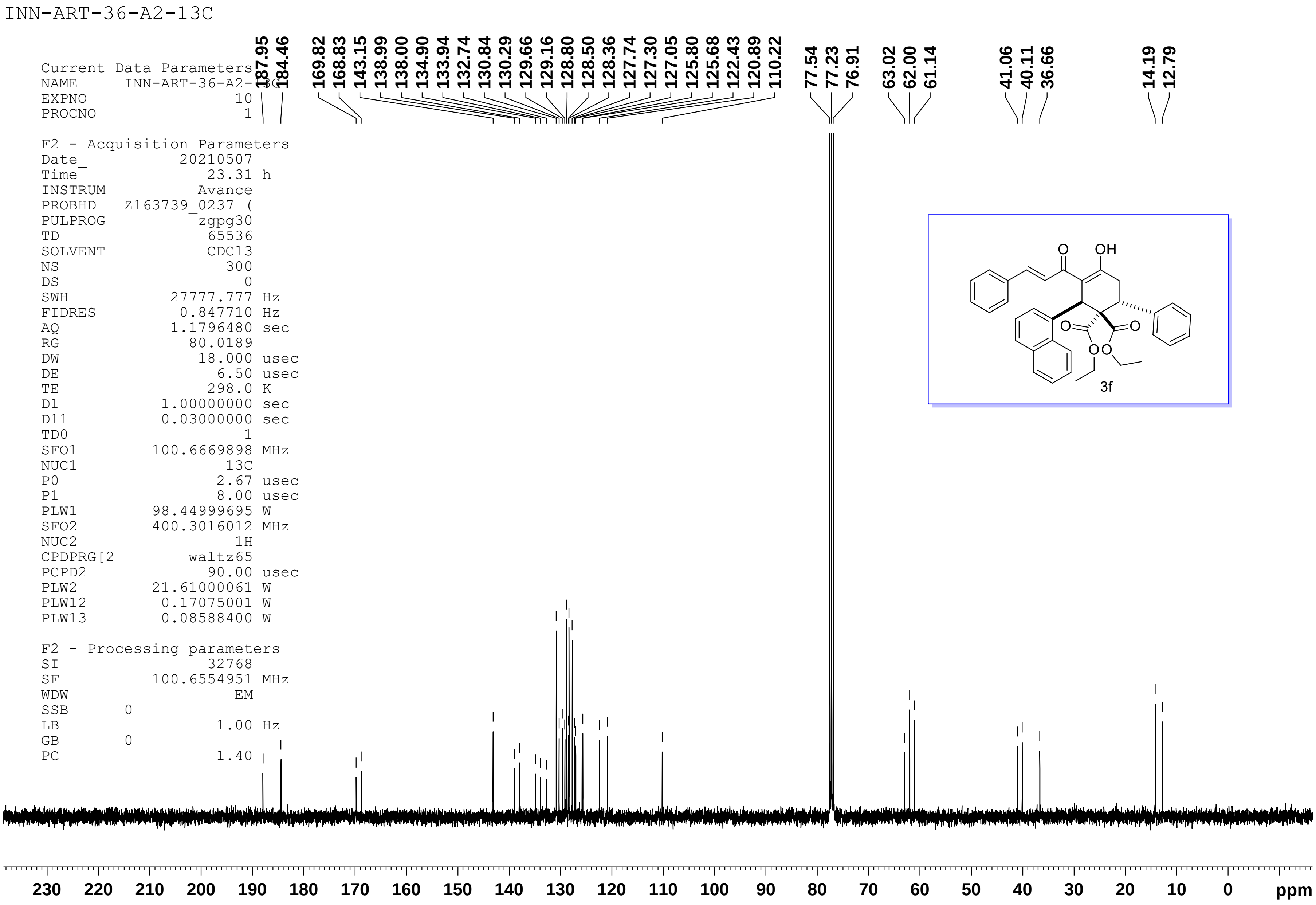

Fig S29. ^{13}C NMR spectrum of 3f

INN-ART-36-A2-13C

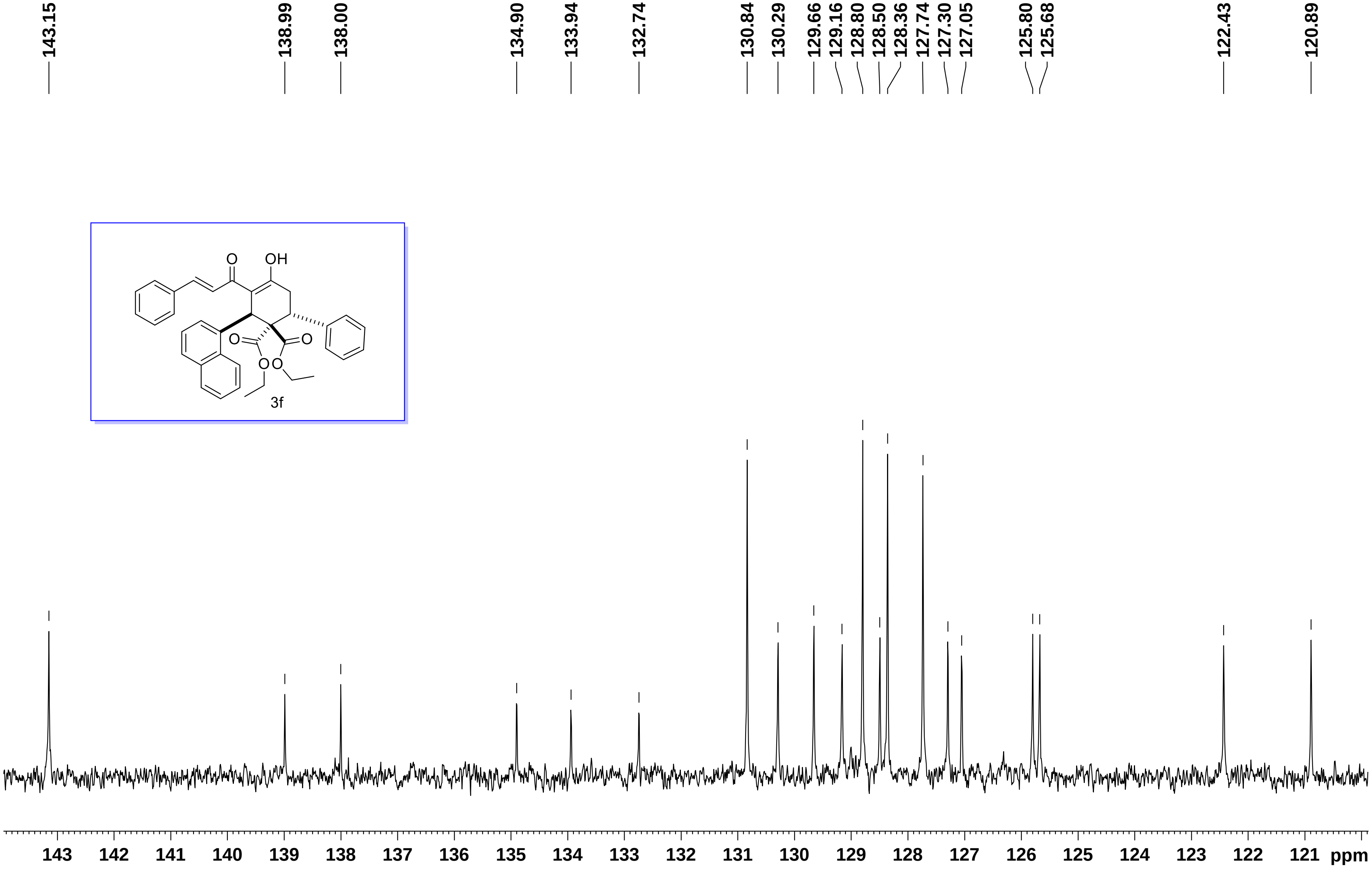

Fig S30. ^{13}C NMR spectrum (exapansion) of 3f

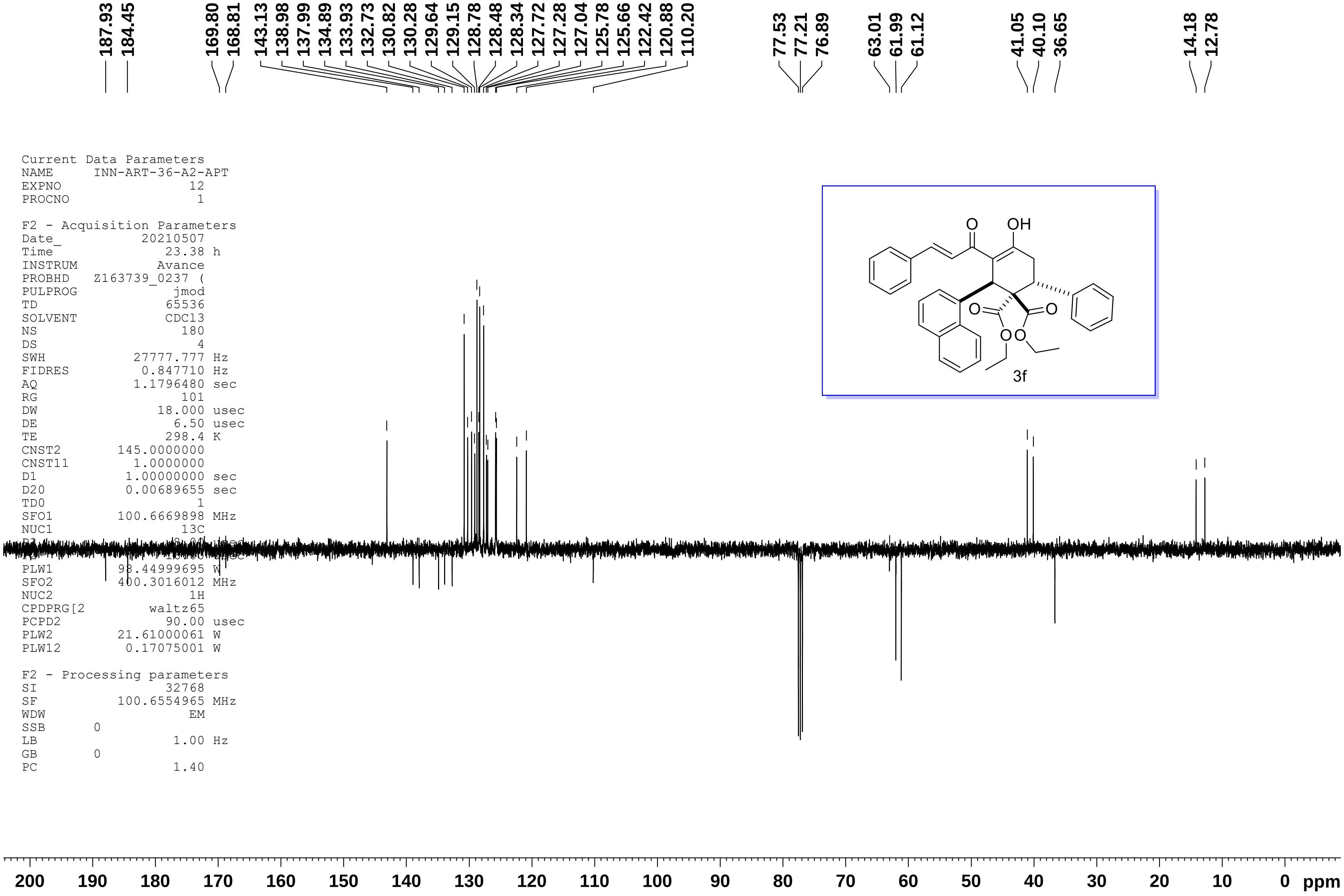

Fig S31. ^{13}C-APT spectrum of 3f

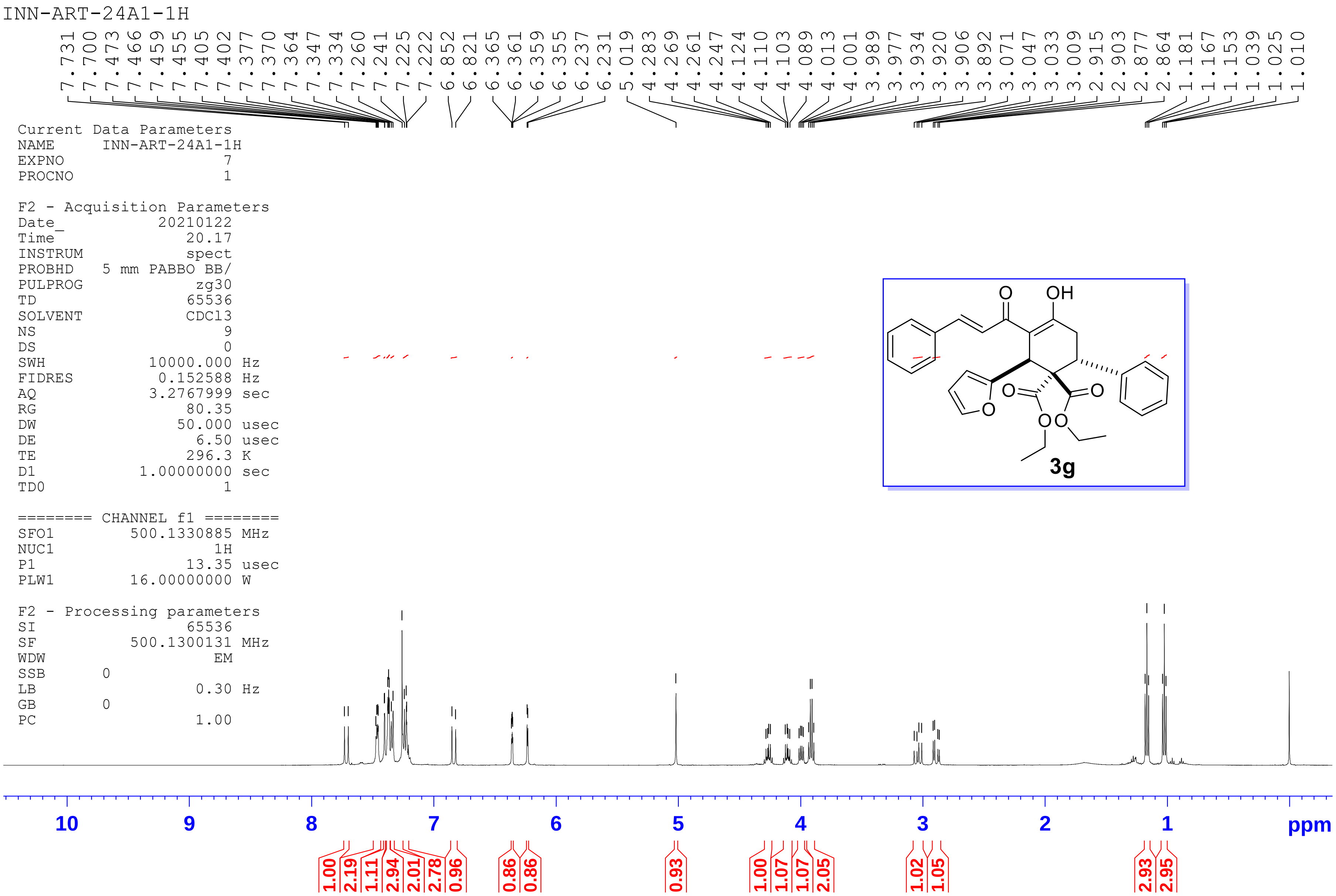

Fig S32. ^{1}H NMR spectrum of 3g

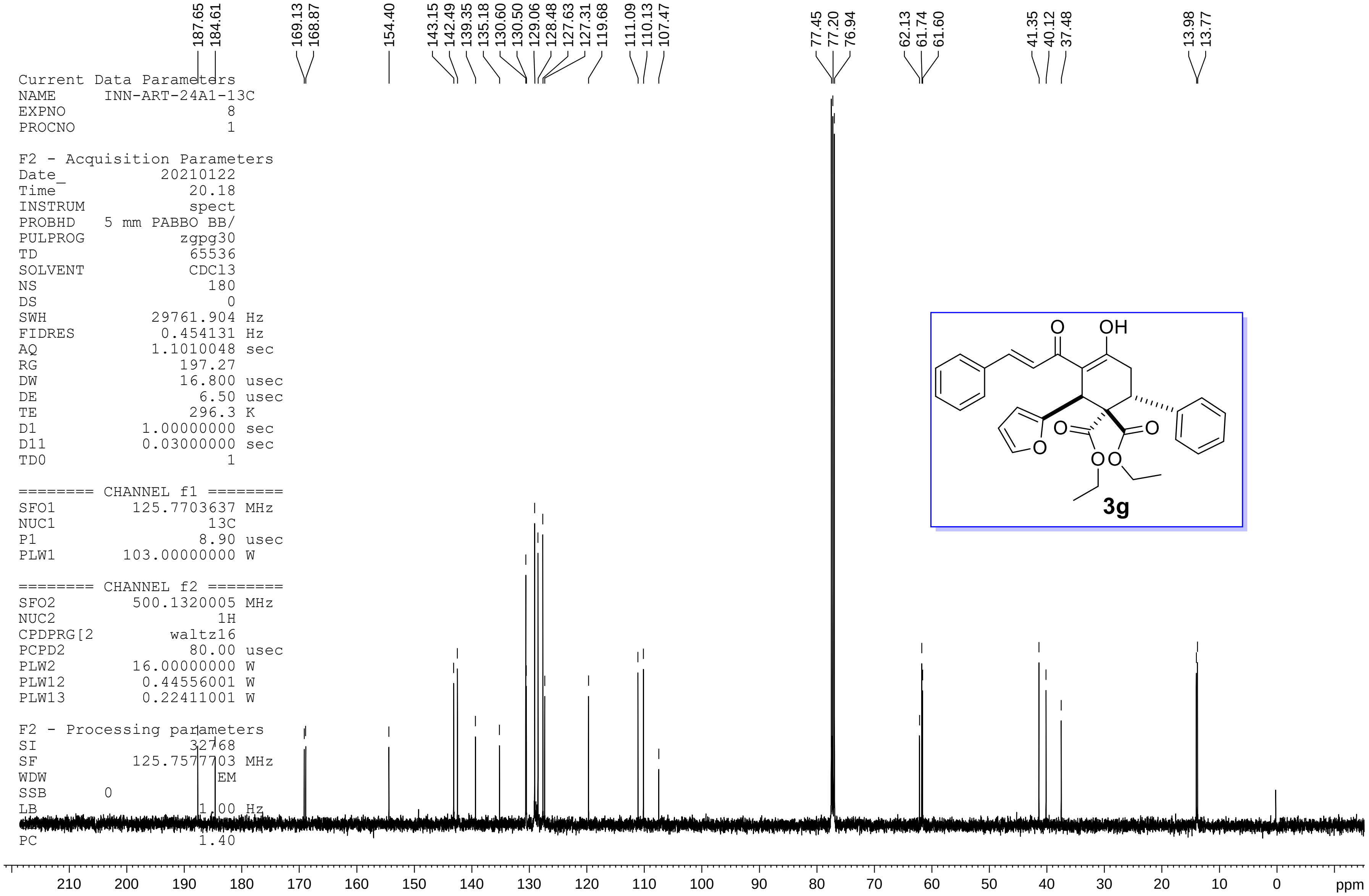

Fig S33. ^{13}C NMR spectrum of 3g

```
Current Data Parameters
NAME      INN-DN-PH-ME-PH-CL-1H
EXPNO                     1
PROCNO                    1

F2 - Acquisition Parameters
Date_              20150711
Time                   5.47
INSTRUM               spect
PROBHD    5 mm PABBO BB-
PULPROG                zg30
TD                    54274
SOLVENT               CDCl3
NS                       19
DS                        0
SWH                8223.685 Hz
FIDRES             0.151522 Hz
AQ                3.2998593 sec
RG                       32
DW                   60.800 usec
DE                     6.50 usec
TE                    299.3 K
D1               1.00000000 sec
TD0                       1

======== CHANNEL f1 ========
NUC1                     1H
P1                    14.75 usec
PL1                   -1.00 dB
PL1W            10.56200695 W
SFO1            400.1340013 MHz

F2 - Processing parameters
SI                    32768
SF              400.1300096 MHz
WDW                      EM
SSB      0
LB                     0.30 Hz
GB       0
PC                     1.00
```

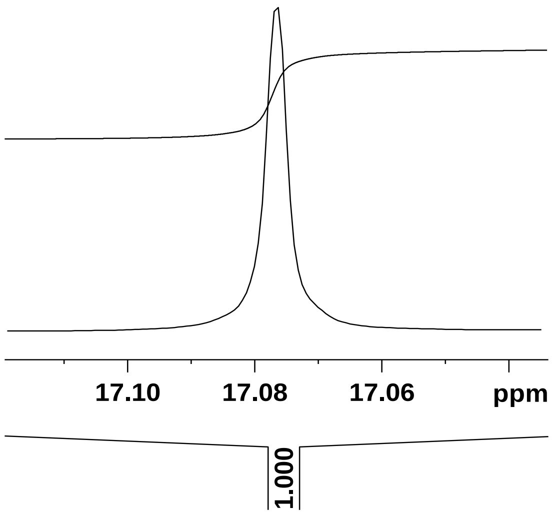

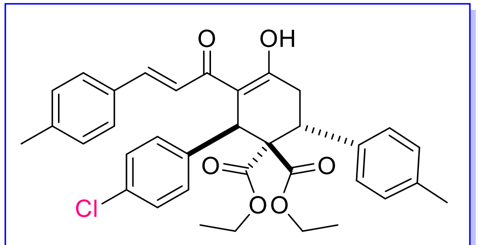

3i

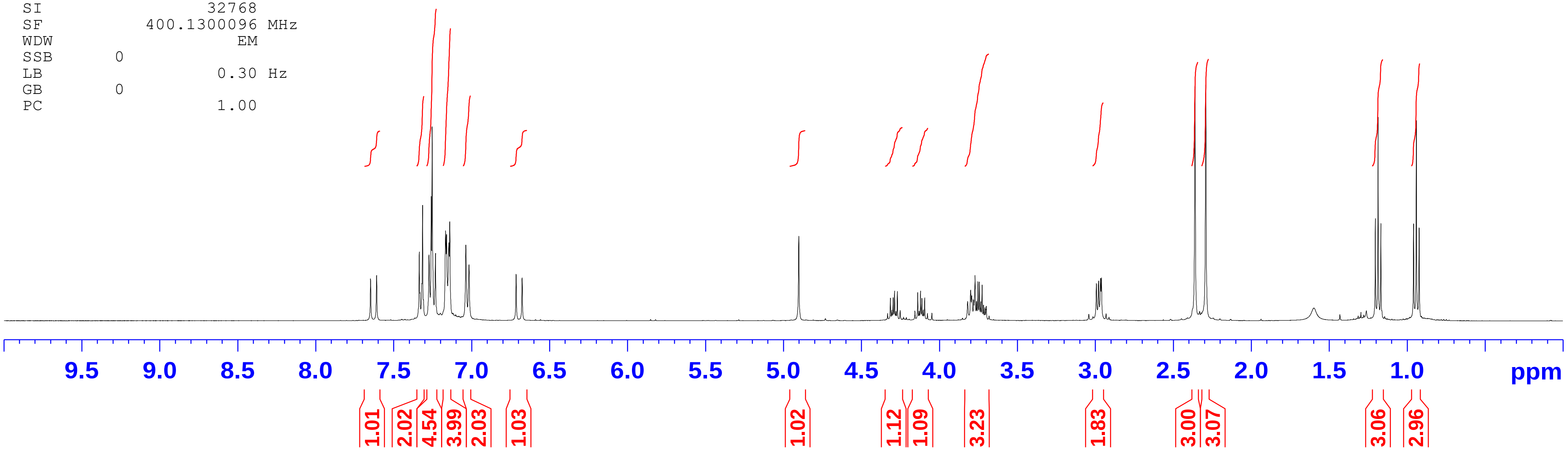

Fig S34. ^{1}H NMR spectrum of 3i

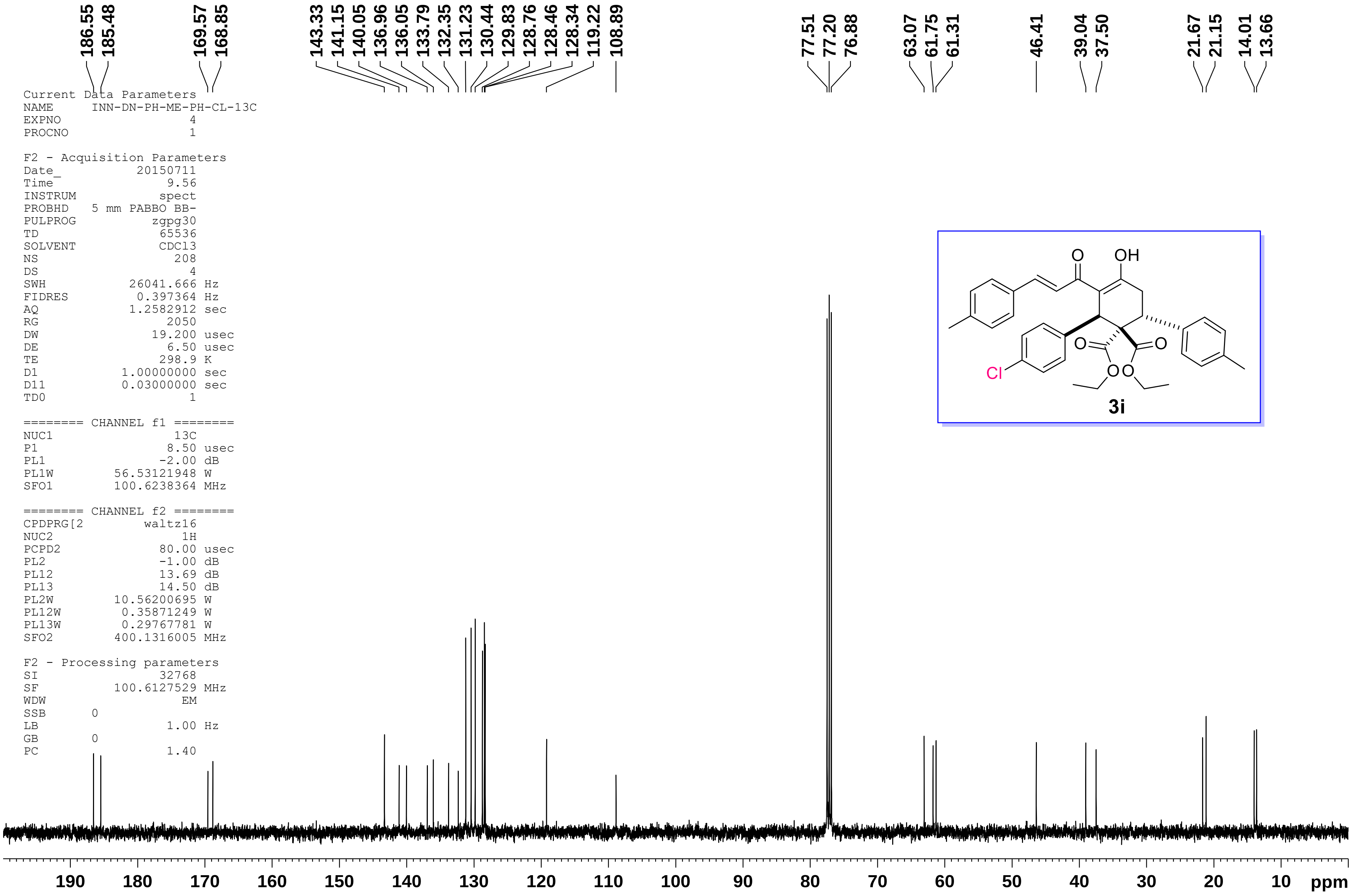

Fig S35. ^{13}C NMR spectrum of 3i

```
Current Data Parameters
NAME      INN-DN-PH-BR-PH-CL-1H
EXPNO                     2
PROCNO                    1

F2 - Acquisition Parameters
Date_              20150711
Time                   5.51
INSTRUM               spect
PROBHD   5 mm PABBO BB-
PULPROG                zg30
TD                    54274
SOLVENT               CDCl3
NS                       13
DS                        0
SWH            8223.685 Hz
FIDRES         0.151522 Hz
AQ            3.2998593 sec
RG                       32
DW               60.800 usec
DE                 6.50 usec
TE                299.4 K
D1           1.00000000 sec
TD0                       1

======== CHANNEL f1 ========
NUC1                     1H
P1                 14.75 usec
PL1                -1.00 dB
PL1W        10.56200695 W
SFO1        400.1340013 MHz

F2 - Processing parameters
SI                    32768
SF          400.1300100 MHz
WDW                      EM
SSB      0
LB                 0.30 Hz
GB       0
PC                     1.00
```

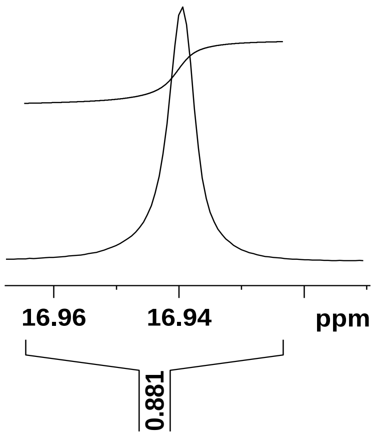

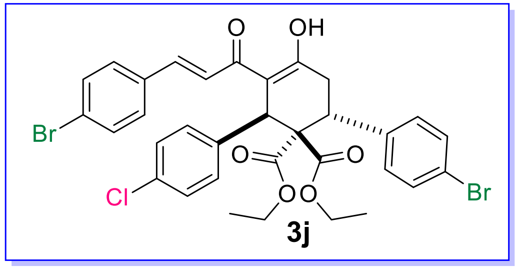

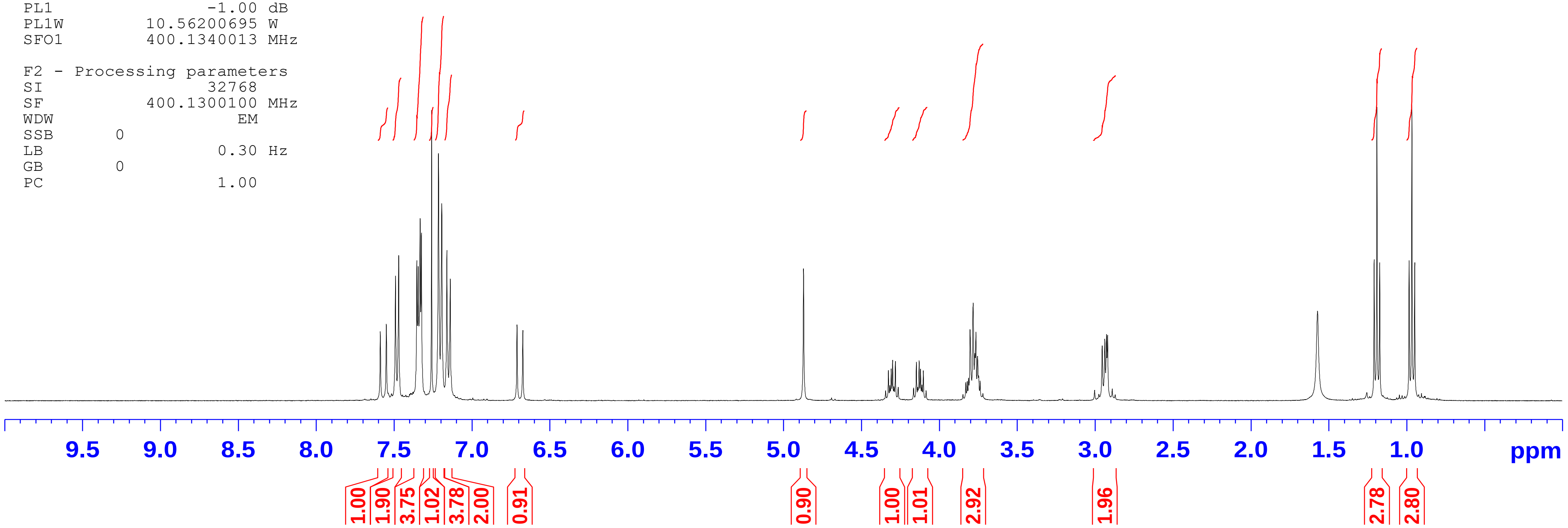

Fig S36. ^{1}H NMR spectrum of 3j

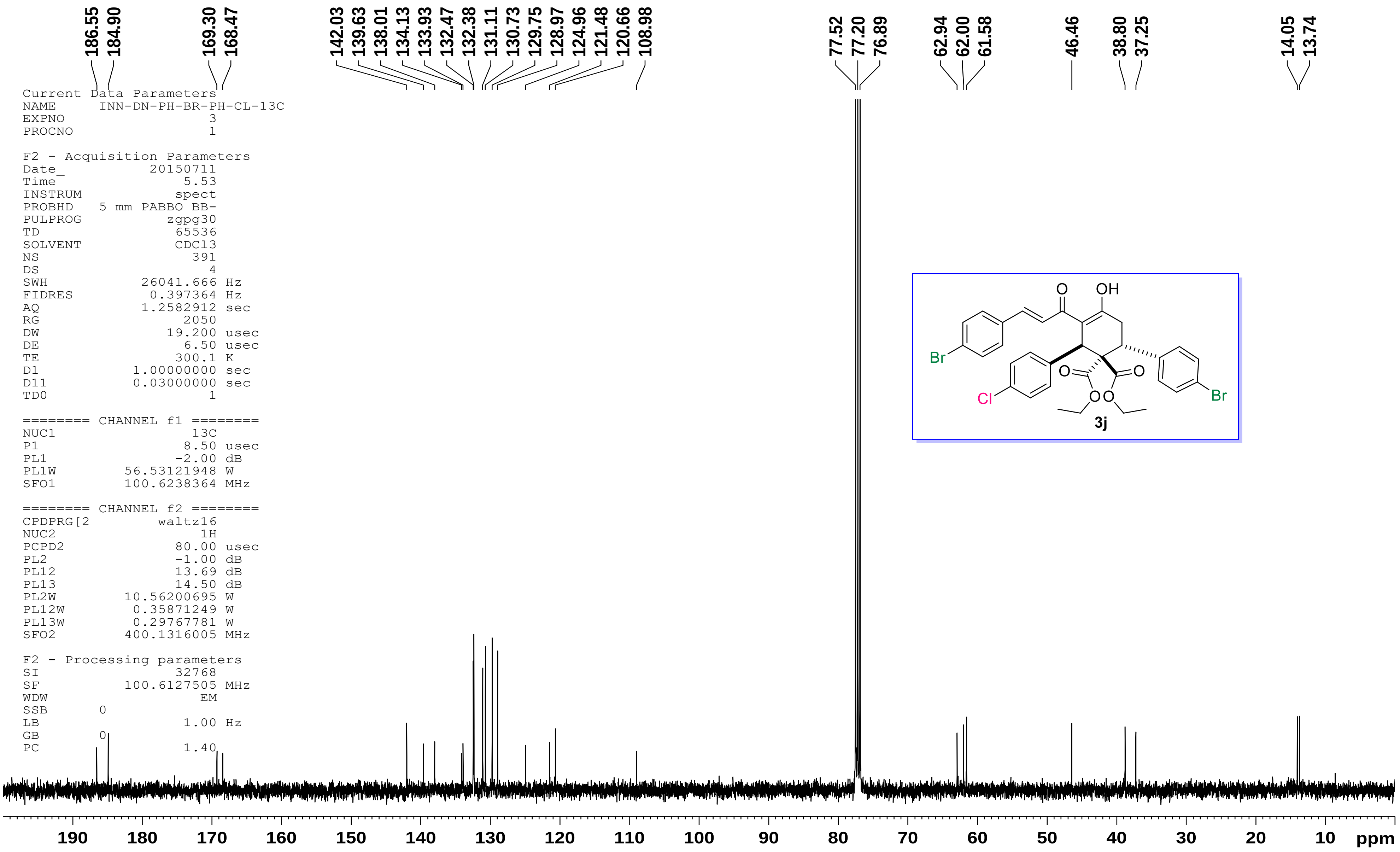

Fig S37. ^{13}**C NMR Spectrum of 3j**

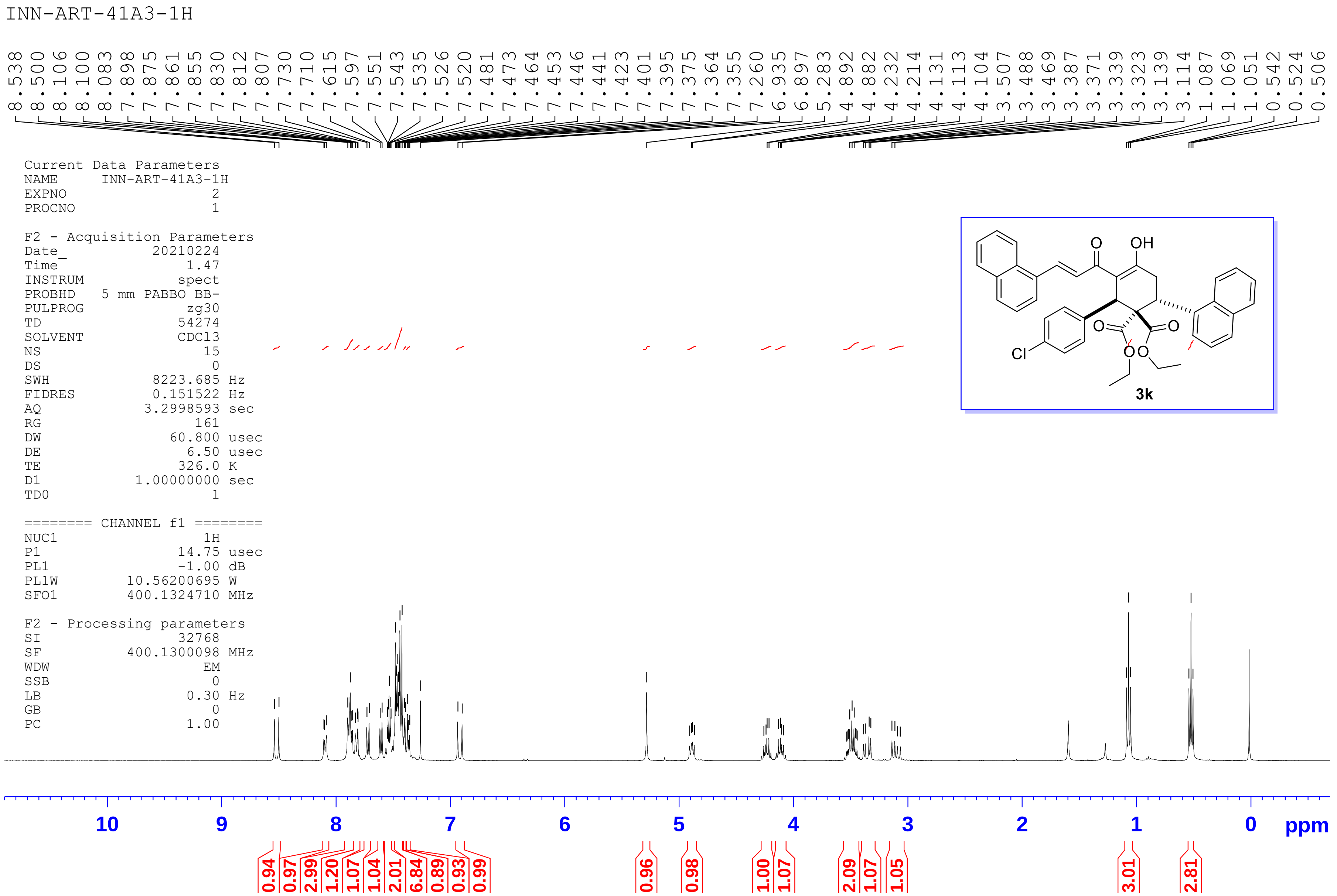

Fig S38. ^{1}H NMR spectrum of 3k

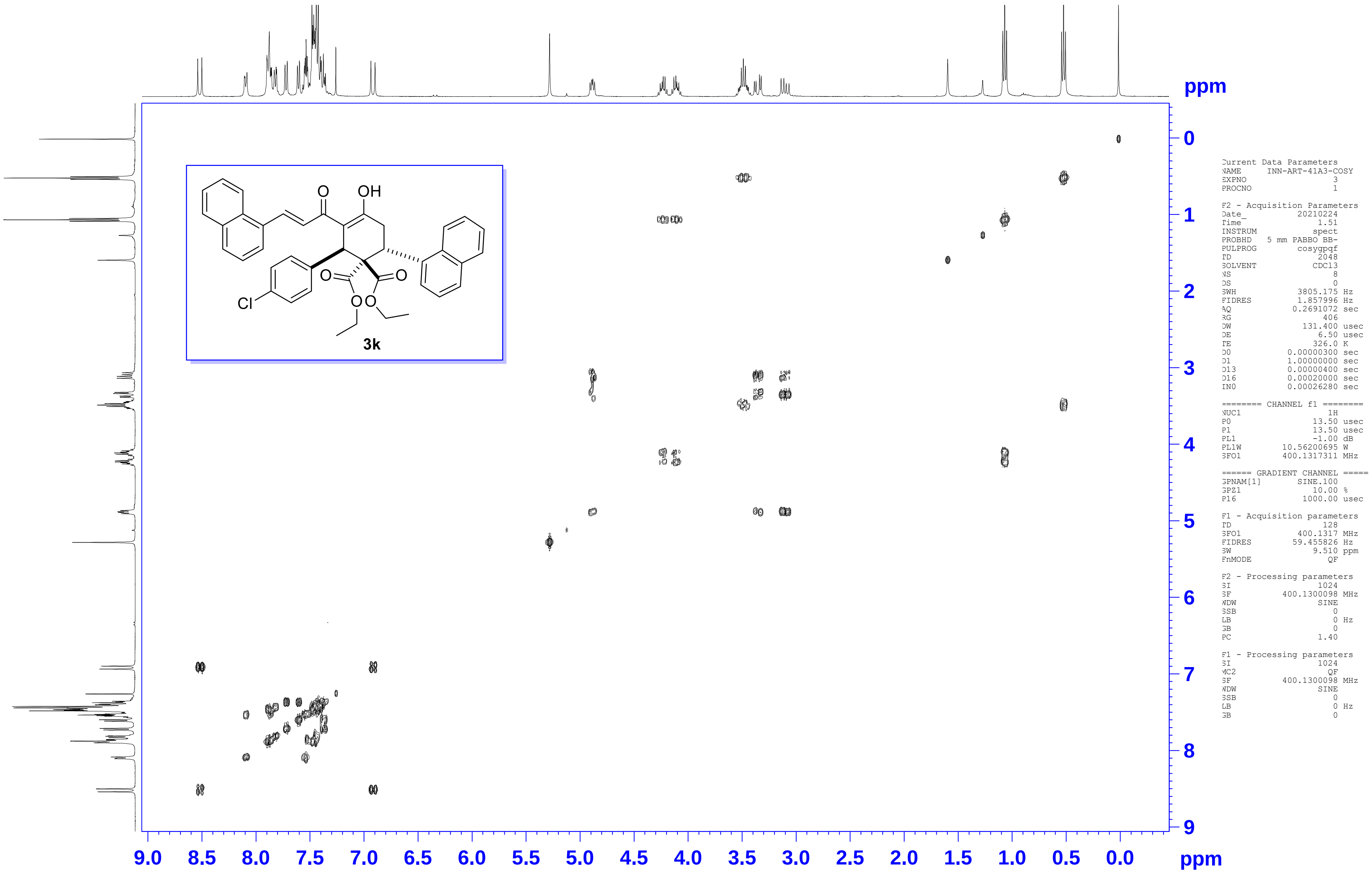

Fig S39. ^{1}H-^{1}H COSY NMR spectrum of 3k

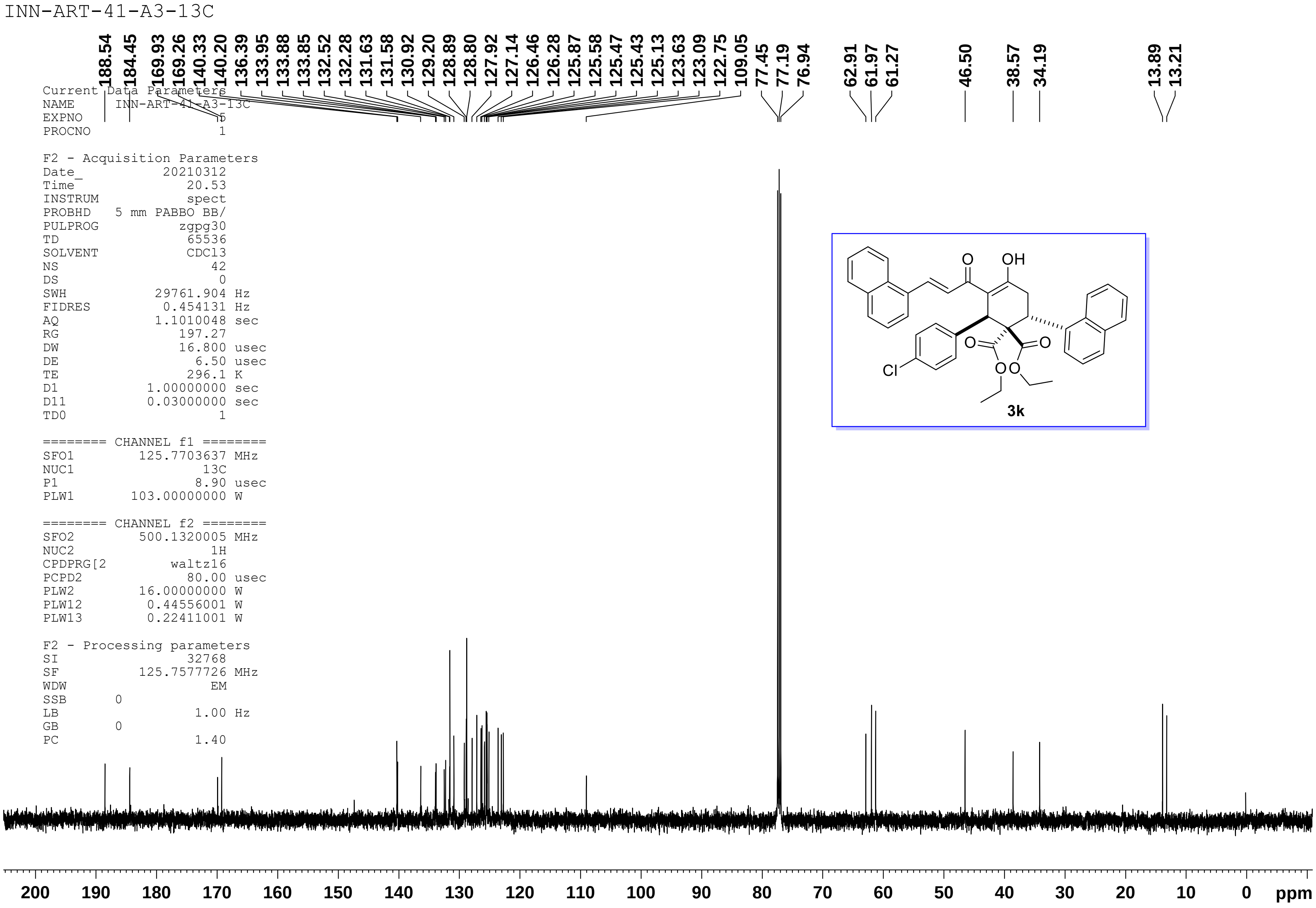

Fig S40. ^{13}**C NMR spectrum of 3k**

INN-ART-41-A3-13C

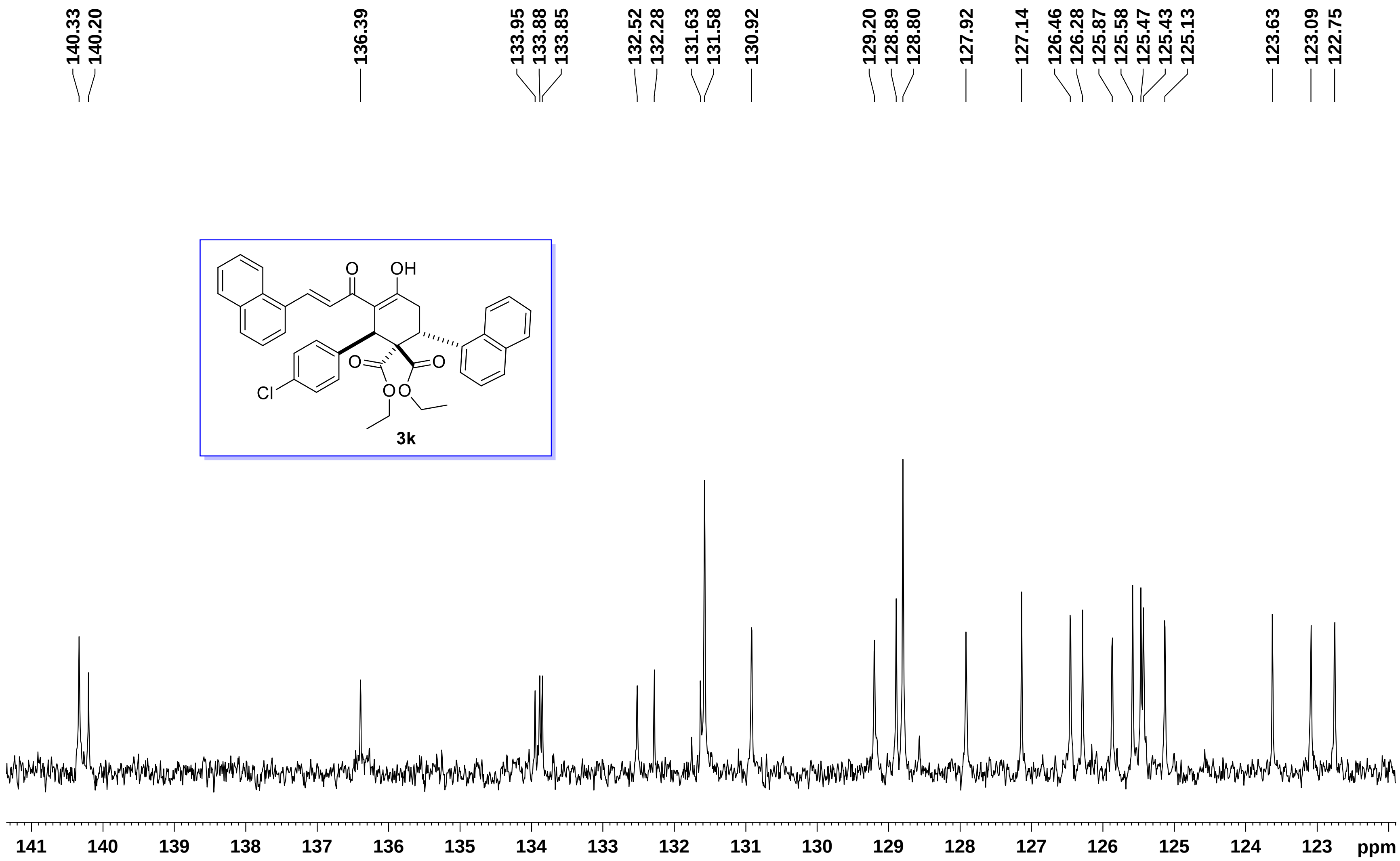

Fig S41. ^{13}C NMR spectrum expansion of 3k

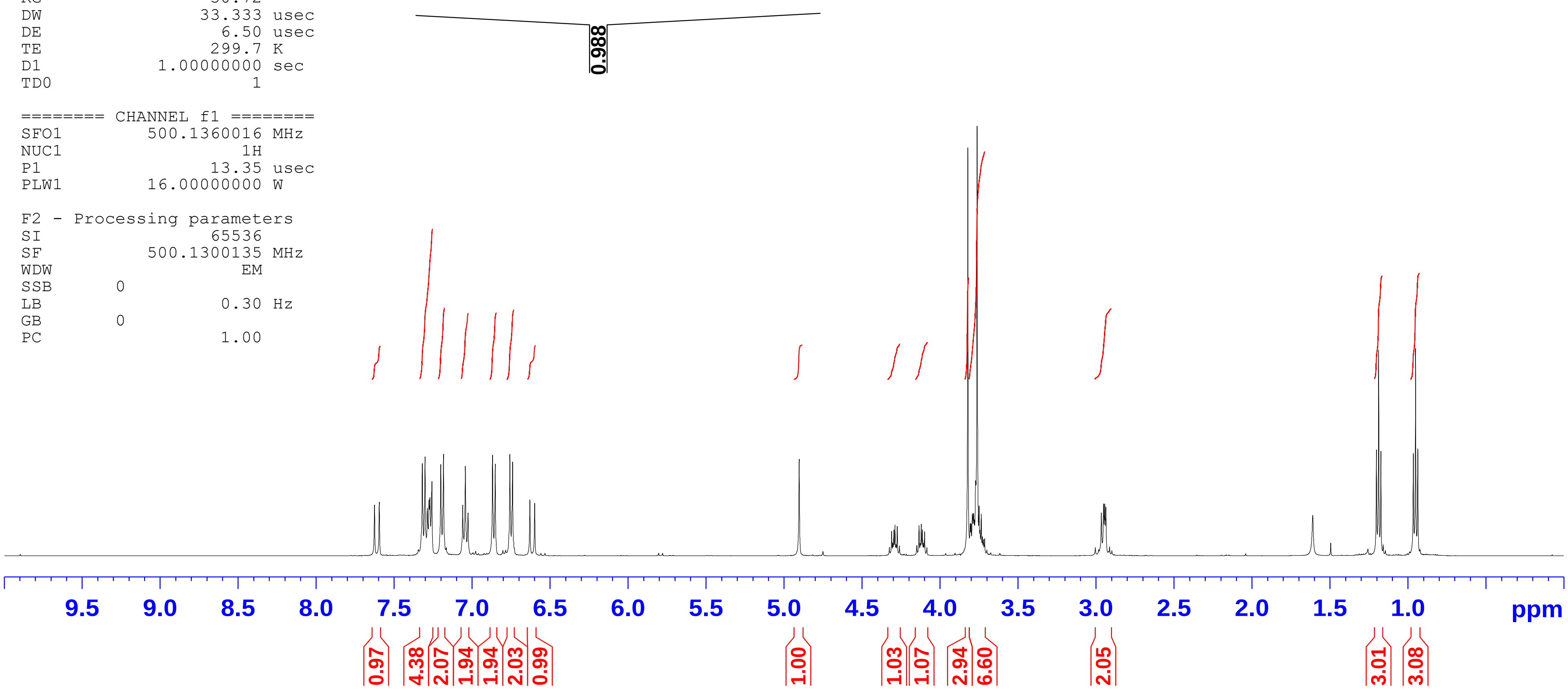

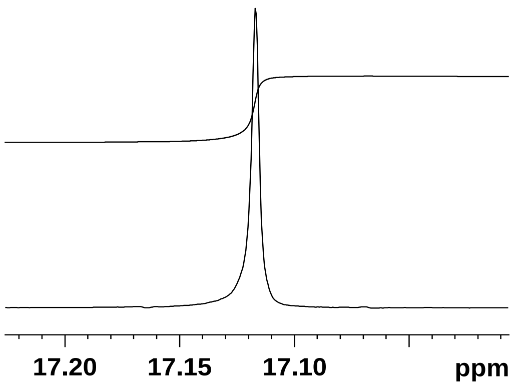

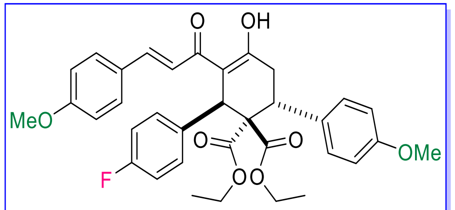

3l

Fig S42. ^{1}H NMR Spectrum of 3l

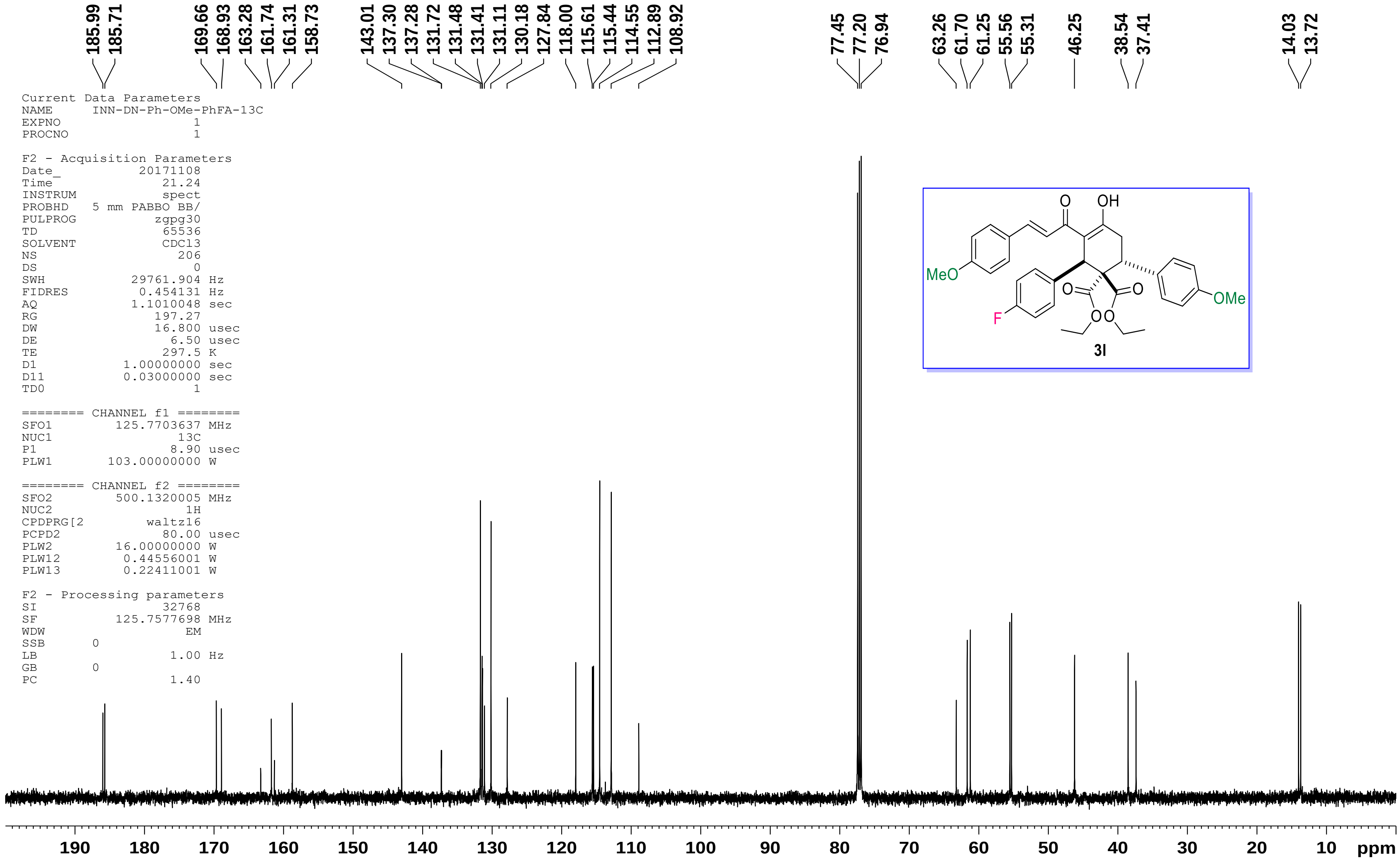

Fig S43. ^{13}C NMR Spectrum of 3l

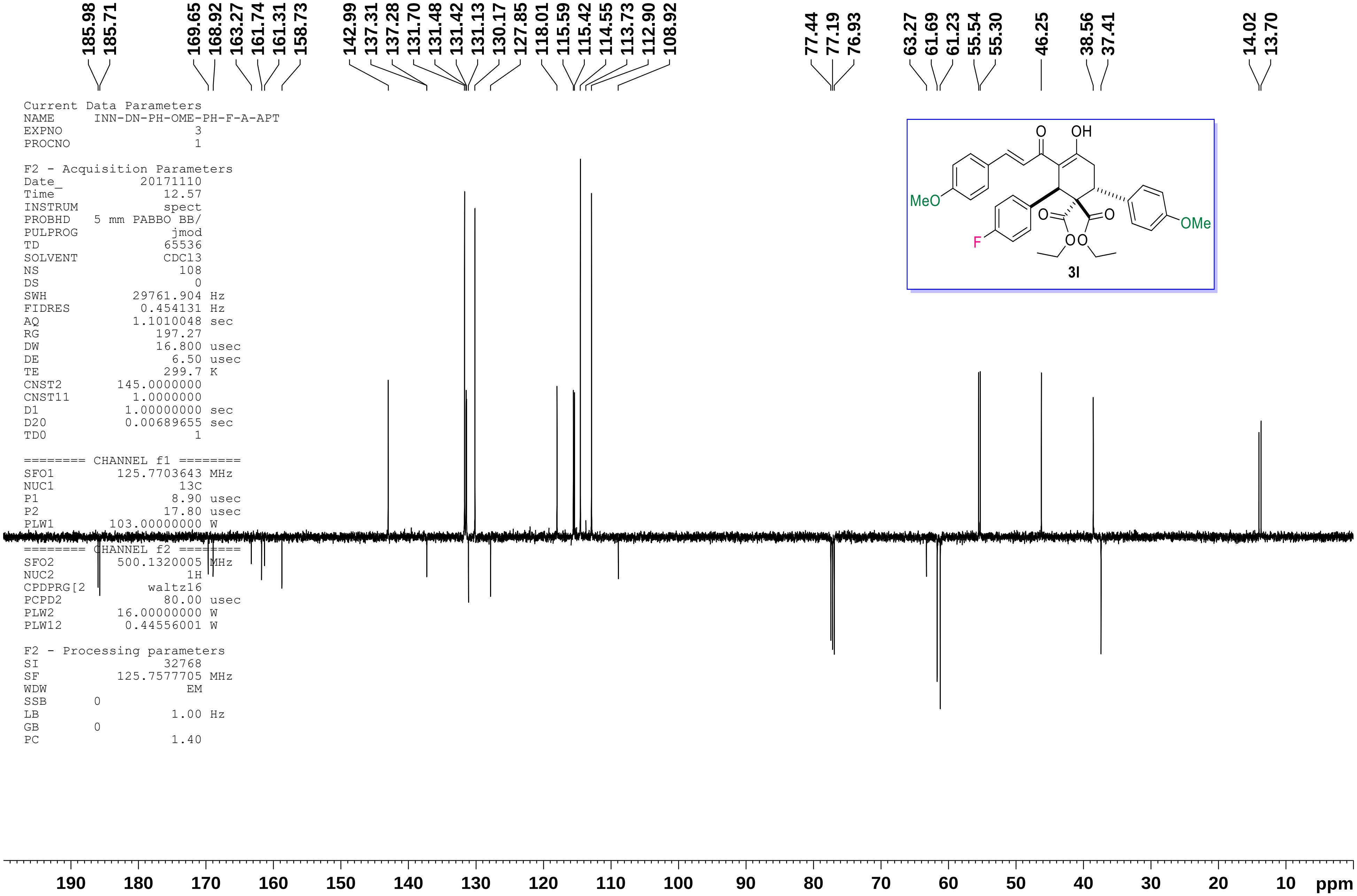

Fig S44. APT spectrum of 3l

```
Current Data Parameters
NAME       INN-ART-45-A1-19F
EXPNO                     4
PROCNO                    1

F2 - Acquisition Parameters
Date_              20210508
Time                   9.46
INSTRUM               spect
PROBHD    5 mm PABBO BB/
PULPROG              zgflqn
TD                   131072
SOLVENT               CDCl3
NS                       30
DS                        0
SWH              113636.367 Hz
FIDRES             0.866977 Hz
AQ                0.5767168 sec
RG                   119.07
DW                    4.400 usec
DE                     6.50 usec
TE                    297.7 K
D1               1.00000000 sec
TD0                       1

======== CHANNEL f1 ========
SFO1            470.5453180 MHz
NUC1                    19F
P1                    19.75 usec
PLW1            55.00000000 W

F2 - Processing parameters
SI                    65536
SF              470.5923770 MHz
WDW                      EM
SSB       0
LB                     0.30 Hz
GB        0
PC                     1.00
```

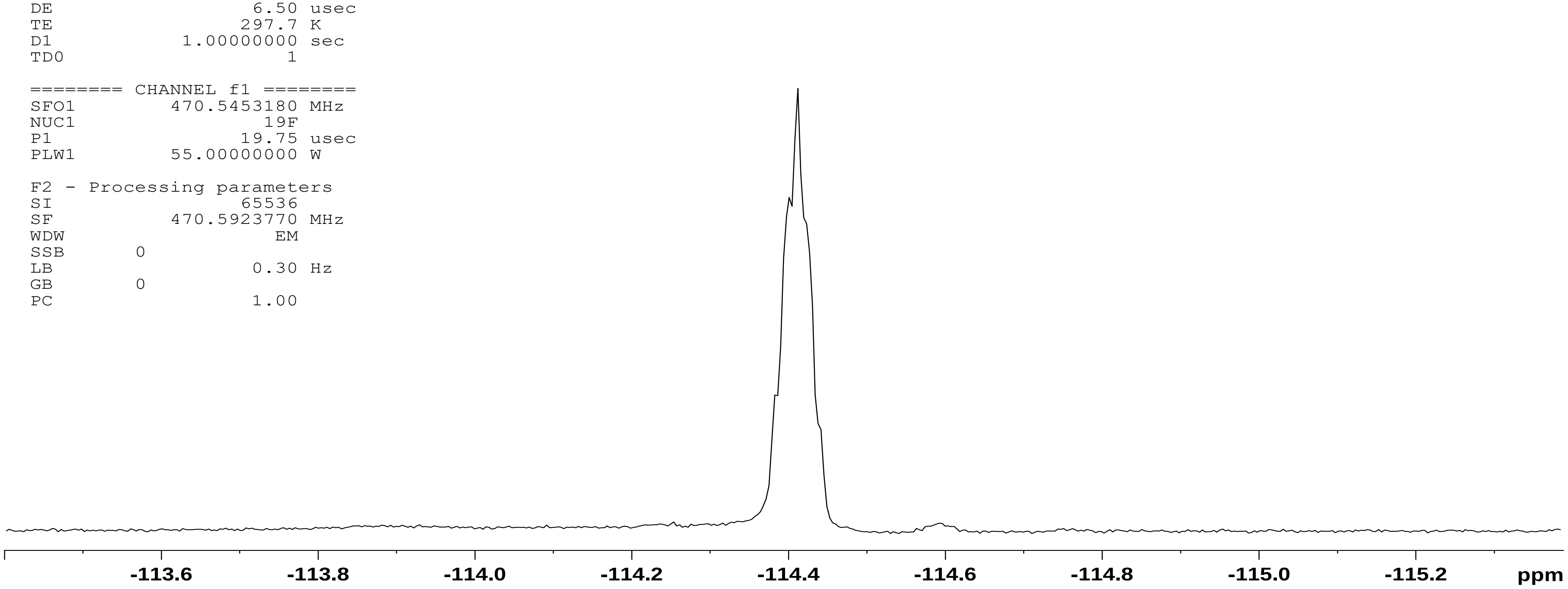

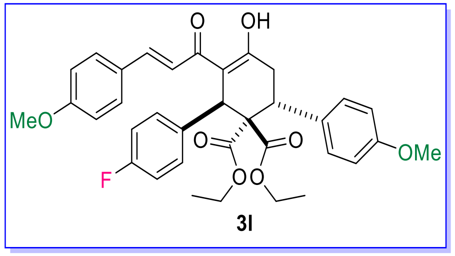

Fig S45. ^{19}F NMR spectrum of 3l

```
Current Data Parameters
NAME        INN-DN-PH-OME-PH-ME-A
EXPNO                          1
PROCNO                         1

F2 - Acquisition Parameters
Date_               20171110
Time                   12.53
INSTRUM                spect
PROBHD    5 mm PABBO BB/
PULPROG                 zg30
TD                     65536
SOLVENT                CDCl3
NS                        11
DS                         0
SWH             15000.000 Hz
FIDRES           0.228882 Hz
AQ              2.1845334 sec
RG                     30.72
DW                  33.333 usec
DE                    6.50 usec
TE                   299.7 K
D1             1.00000000 sec
TD0                        1

======== CHANNEL f1 ========
SFO1         500.1360016 MHz
NUC1                      1H
P1                   13.35 usec
PLW1          16.00000000 W

F2 - Processing parameters
SI                     65536
SF           500.1300130 MHz
WDW                       EM
SSB      0
LB                      0.30 Hz
GB       0
PC                      1.00
```

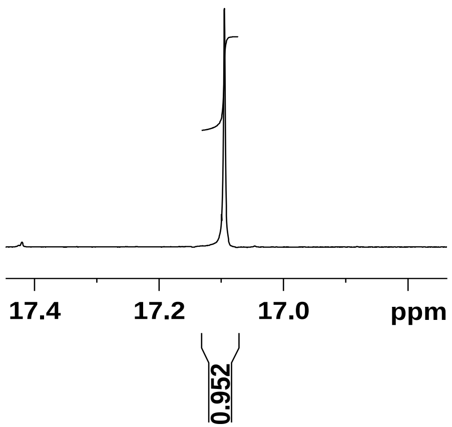

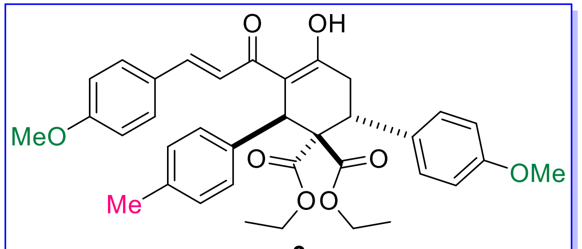

3m

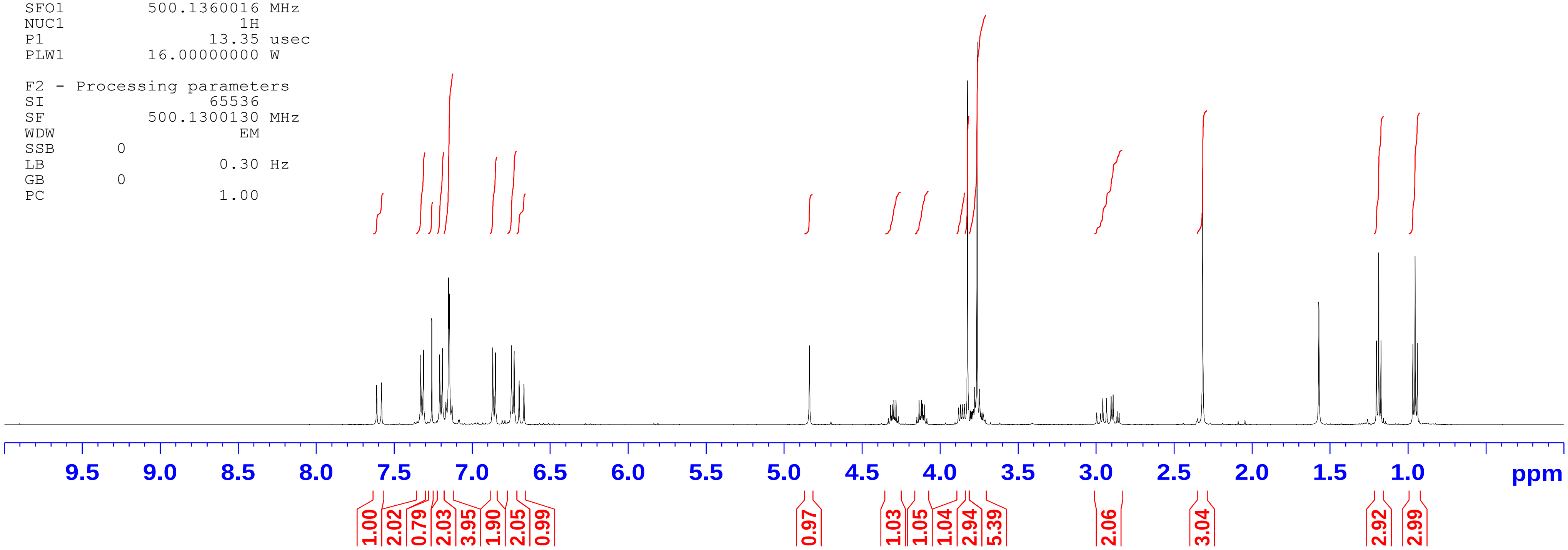

Fig S46. ^{1}H NMR Spectrum of 3m

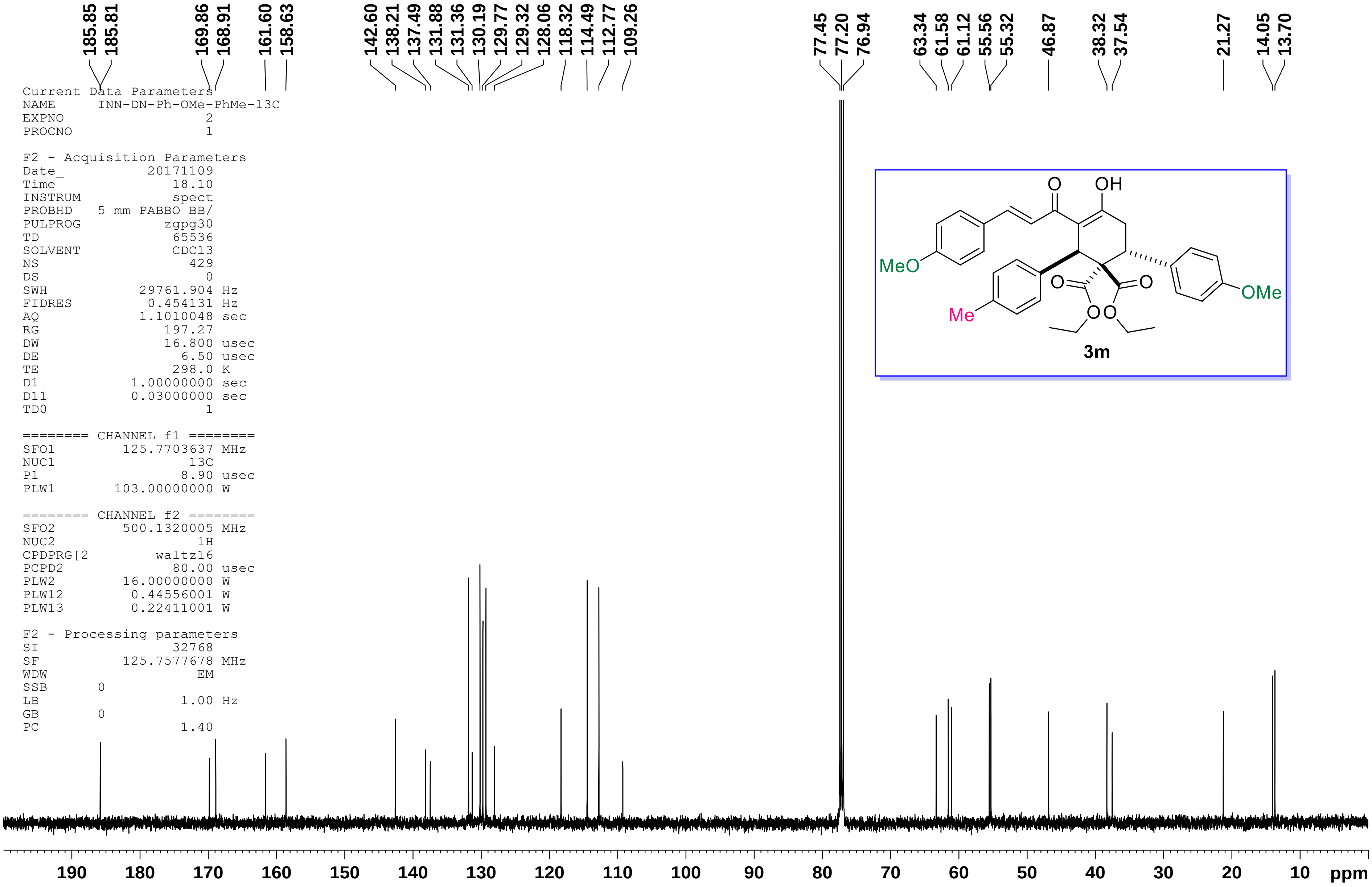

Fig S47. ^{13}C NMR Spectrum of 3m

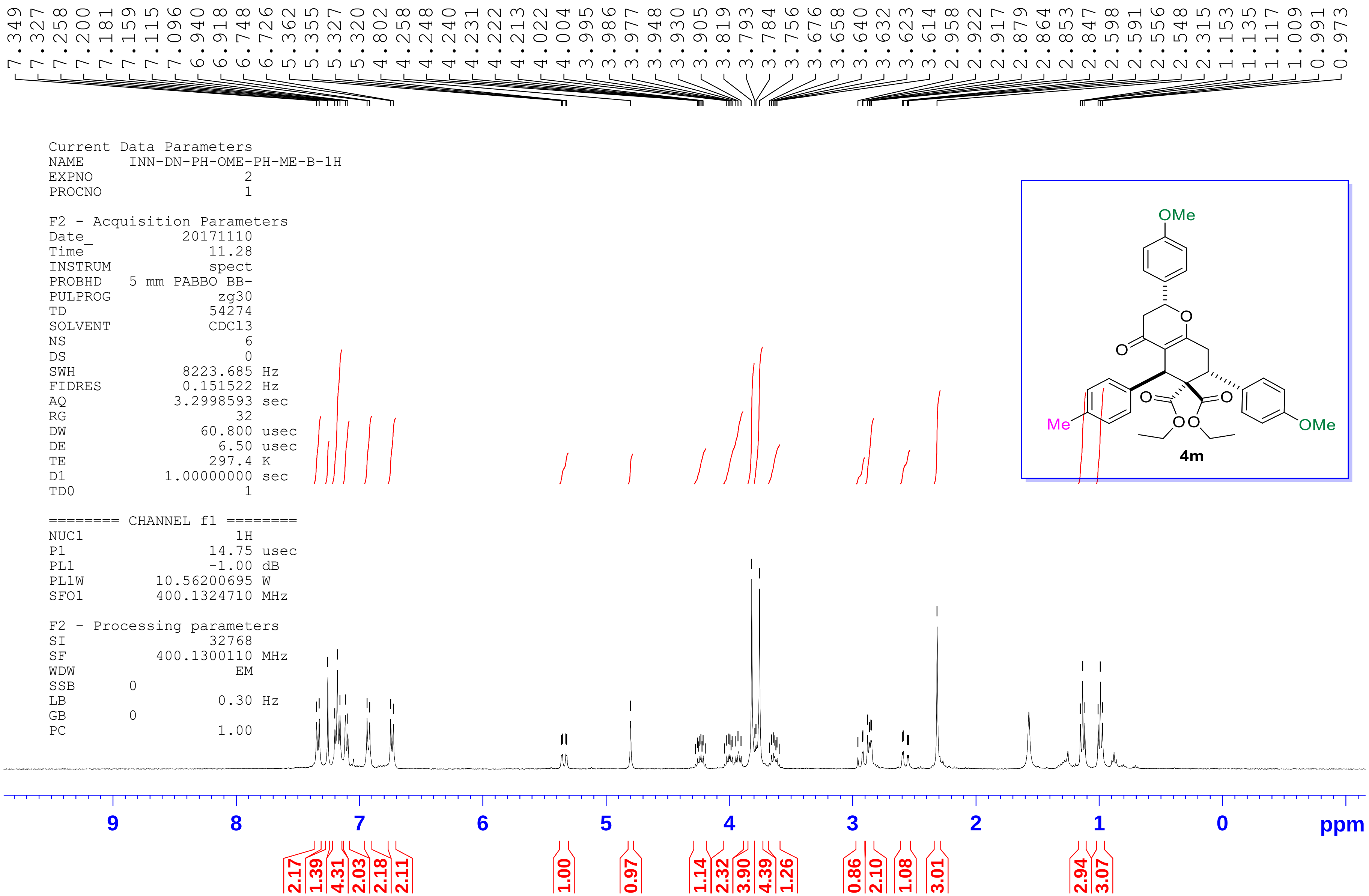

Fig S48. ^{1}H NMR Spectrum of 4m

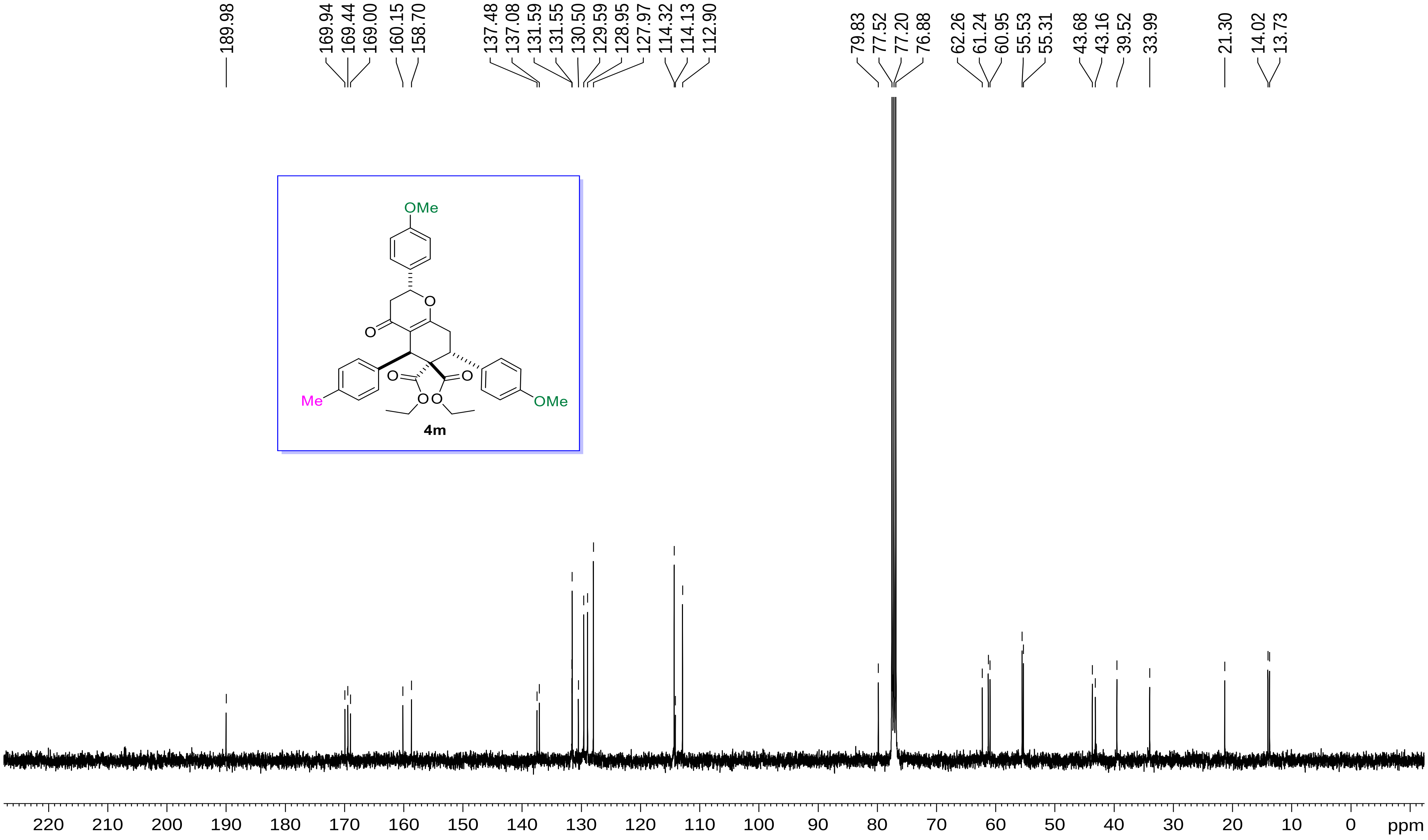

Fig S49. ^{13}C NMR Spectrum of 4m

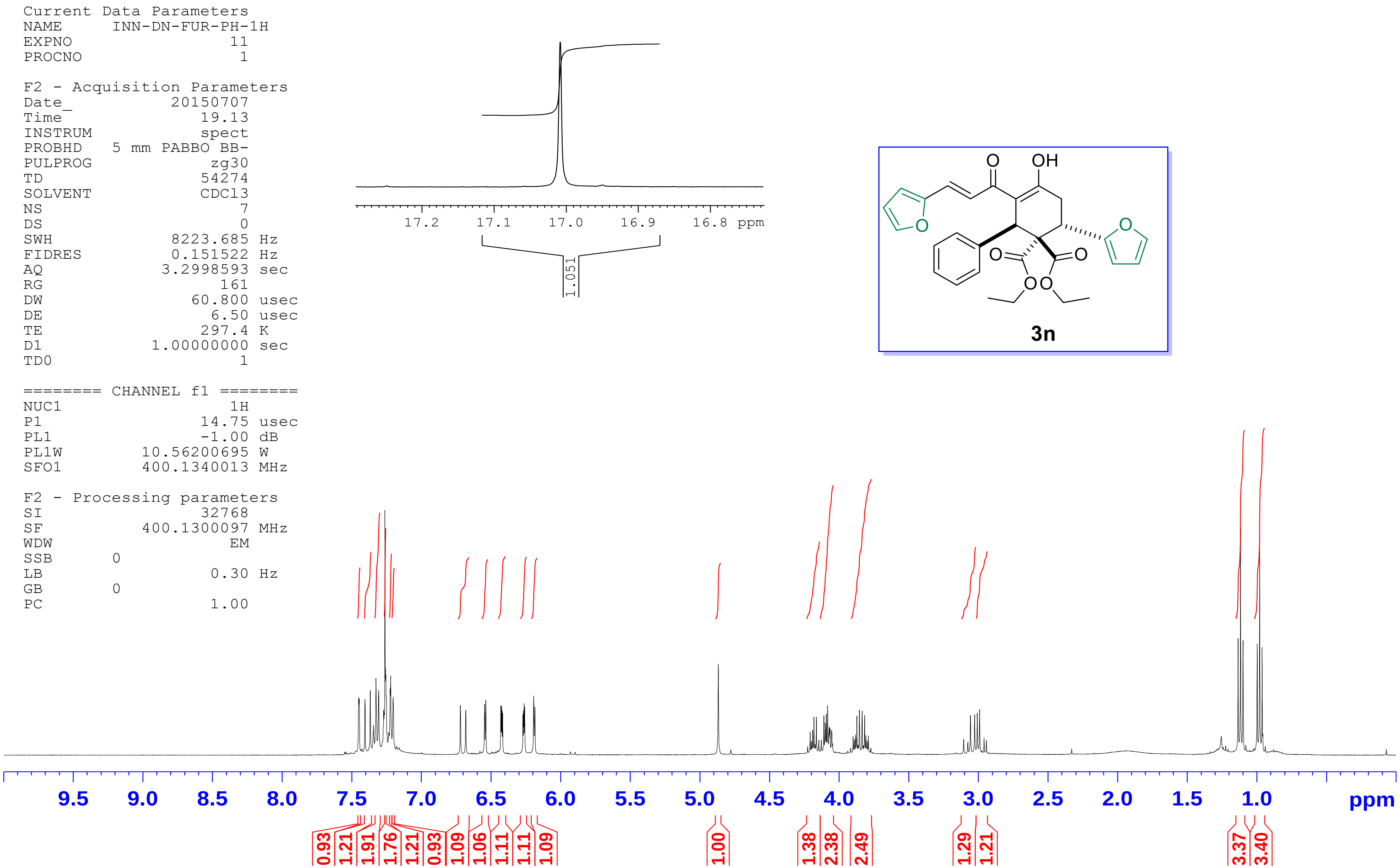

Fig S50. ^{1}H NMR Spectrum of 3n

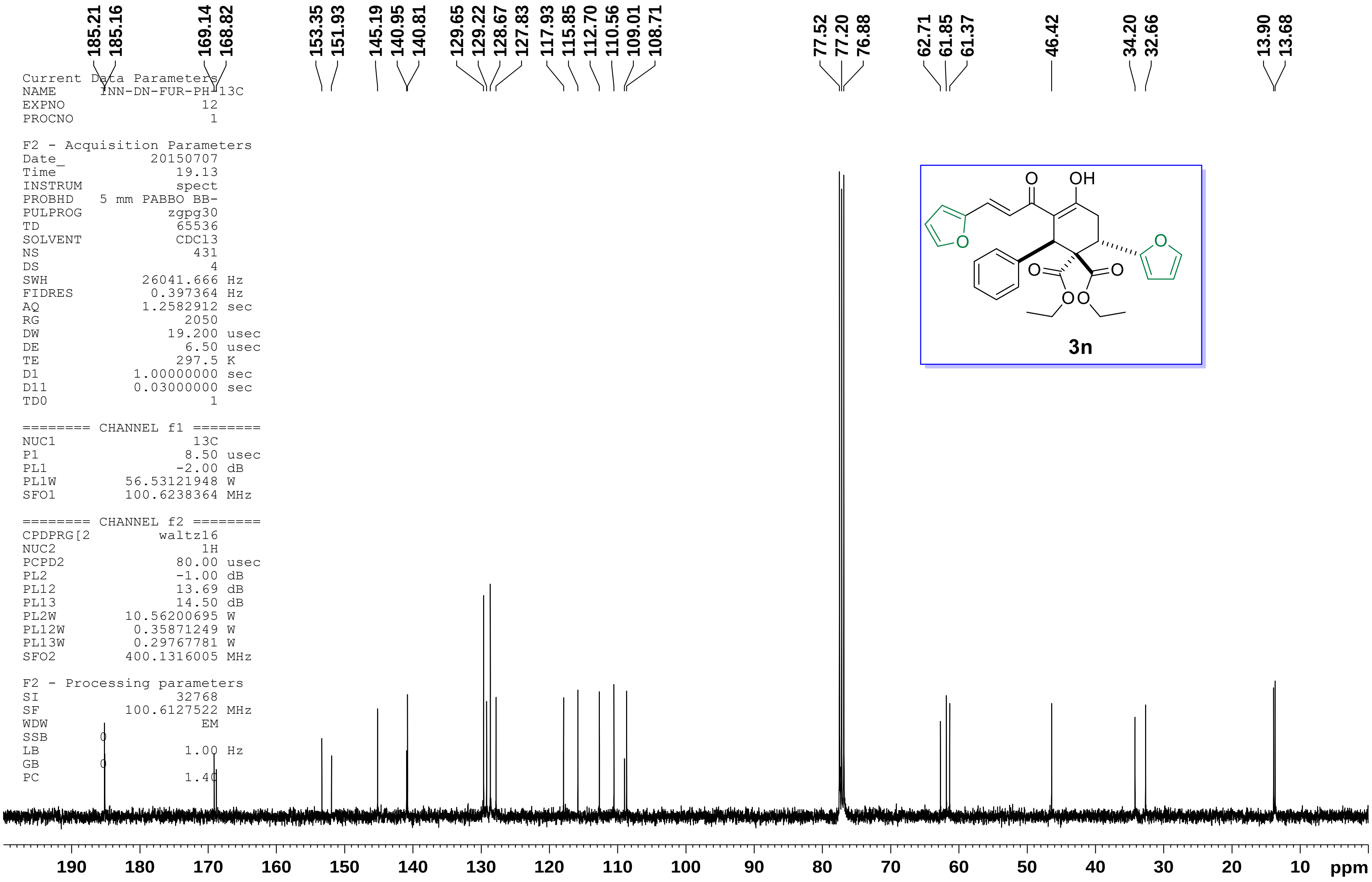

Fig S51. ^{13}C NMR Spectrum of 3n
